# Supplementary material for: The Hydrolytic Activity of Copper(II) Complexes with 1,4,7-Triazacyclononane Derivatives for the Hydrolysis of Phosphate Diesters
Source: Molecules. 2023 Nov 11;28(22):7542. doi: 10.3390/molecules28227542 (PMC10673150; doi:10.3390/molecules28227542)

Electronic Supporting Information

for

Hydrolytic activity of copper(II) complexes with 1,4,7-triazacyclononane derivatives for phosphate ester hydrolysis

Michaela Buziková, Robert Willimetz and Jan Kotek*

Department of Inorganic Chemistry, Charles University, Hlavova 2030, Prague 2, 128 40, Czech Republic. modrej@natur.cuni.cz

Structural formulas of respective ligands

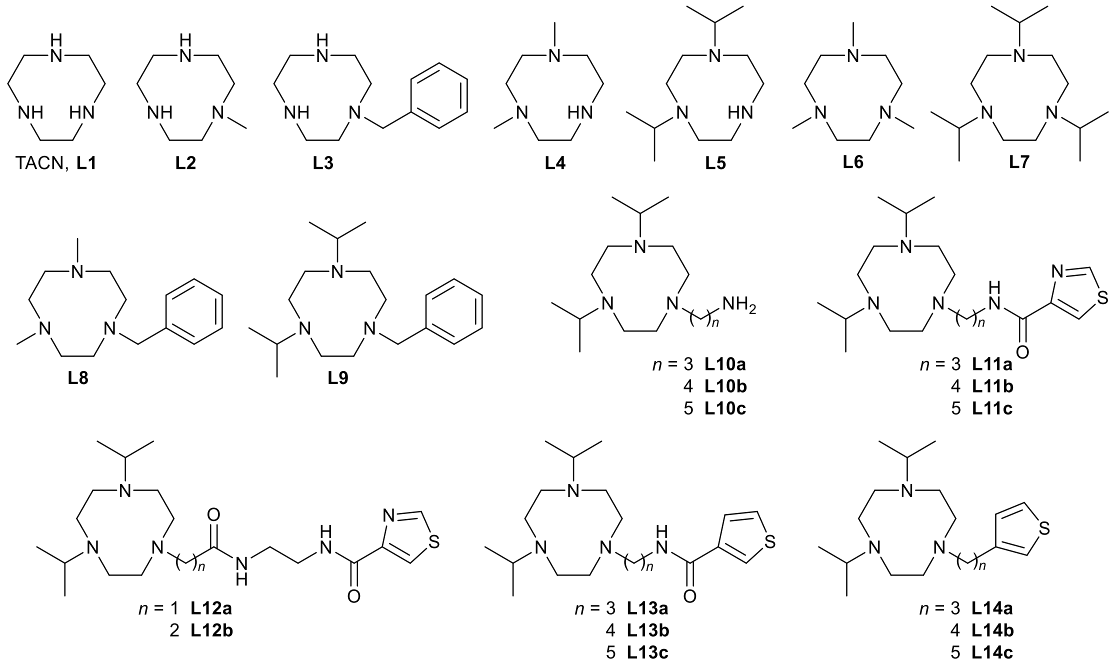

UV-Vis characterization of the studied complexes

Table S1: Absorption maxima of prepared copper(II) complexes and their respective absorption extinction coefficients.

Ligand	λ_{max} [nm]	ε_λ [$dm^3 \cdot mol^{-1} \cdot cm^{-1}$]	Ligand	λ_{max} [nm]	ε_λ [$dm^3 \cdot mol^{-1} \cdot cm^{-1}$]
**L1**	637	67.3	**L11a**	675	23.4
**L2**	636	60.3	**L11b**	641	134
**L3**	629	61.5	**L11c**	642	32.5
**L4**	627	42.1	**L12a**	695	20.8
**L5**	670	101	**L12b**	657	91.0
**L6**	640	67.6	**L13a**	660	41.8
**L8**	634	56.3	**L13b**	640	62.5
**L9**	676	29.7	**L13c**	661	42.8
**L10a**	625	114	**L14a**	656	67.7
**L10b**	640	104	**L14b**	682	61.3
**L10c**	683	72.6	**L14c**	664	45.5

MS characterization of the studied complexes

Table S2: Mass-to-charge ratios of prepared copper(II) complexes found by mass spectrometry.

Cu(II)**L**	*m/z*	Cu(II)**L**	*m/z*
**L1**	[Cu**L1**+TFA]$^+$: 305.1	**L11a**	[Cu**L11a**–H]$^+$: 443.3
**L2**	[Cu**L2**+TFA]$^+$: 319.1	**L11b**	[Cu**L11b**+TFA]$^+$: 585.4
**L3**	[Cu**L3**+TFA]$^+$: 395.3	**L11c**	[Cu**L11c**+TFA]$^+$: 571.4
**L4**	[Cu**L4**+TFA]$^+$: 333.1	**L12a**	[Cu**L12a**+TFA]$^+$: 600.3
**L5**	[Cu**L5**+TFA]$^+$: 389.2	**L12b**	[Cu**L12b**+TFA]$^+$: 614.5
**L6**	[Cu**L6**+TFA]$^+$: 347.2	**L13a**	[Cu**L13a**+TFA]$^+$: 556.3
**L7**	[Cu**L7**+TFA]$^+$: 431.4	**L13b**	[Cu**L13b**+TFA]$^+$: 570.3
**L8**	[Cu**L8**+Cl]$^+$: 345.2	**L13c**	[Cu**L13c**+TFA]$^+$: 584.4
**L9**	[Cu**L9**+TFA]$^+$: 479.3	**L14a**	[Cu**L14a**+TFA]$^+$: 513.3
**L10a**	[Cu**L10a**+TFA]$^+$: 446.3	**L14b**	[Cu**L14b**+TFA]$^+$: 527.3
**L10b**	[Cu**L10b**+TFA]$^+$: 460.3	**L14c**	[Cu**L14c**+TFA]$^+$: 541.3
**L10c**	[Cu**L10c**+TFA]$^+$: 474.4		

Note: TFA = $CF_3CO_2^-$ from trifluoroacetic acid used as a modificator of a mobile phase used for dilution of the sample.

NMR and MS spectra of novel compounds

2a

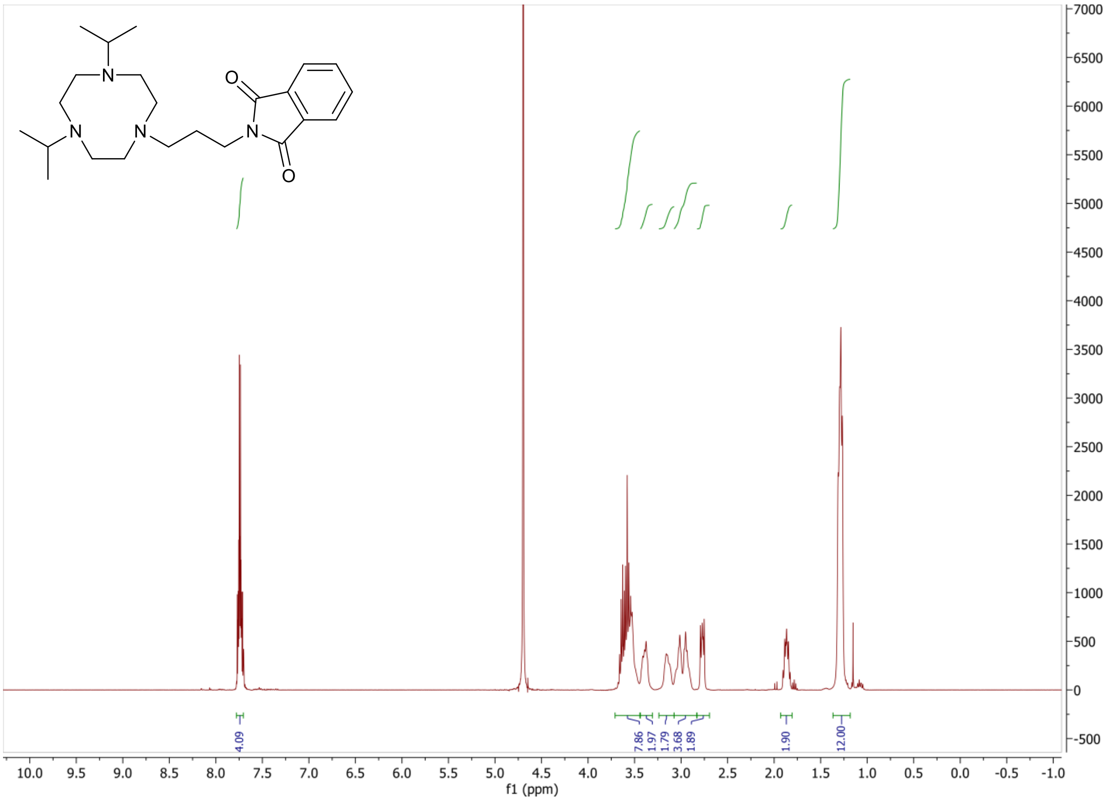

^{1}H (D_2O)

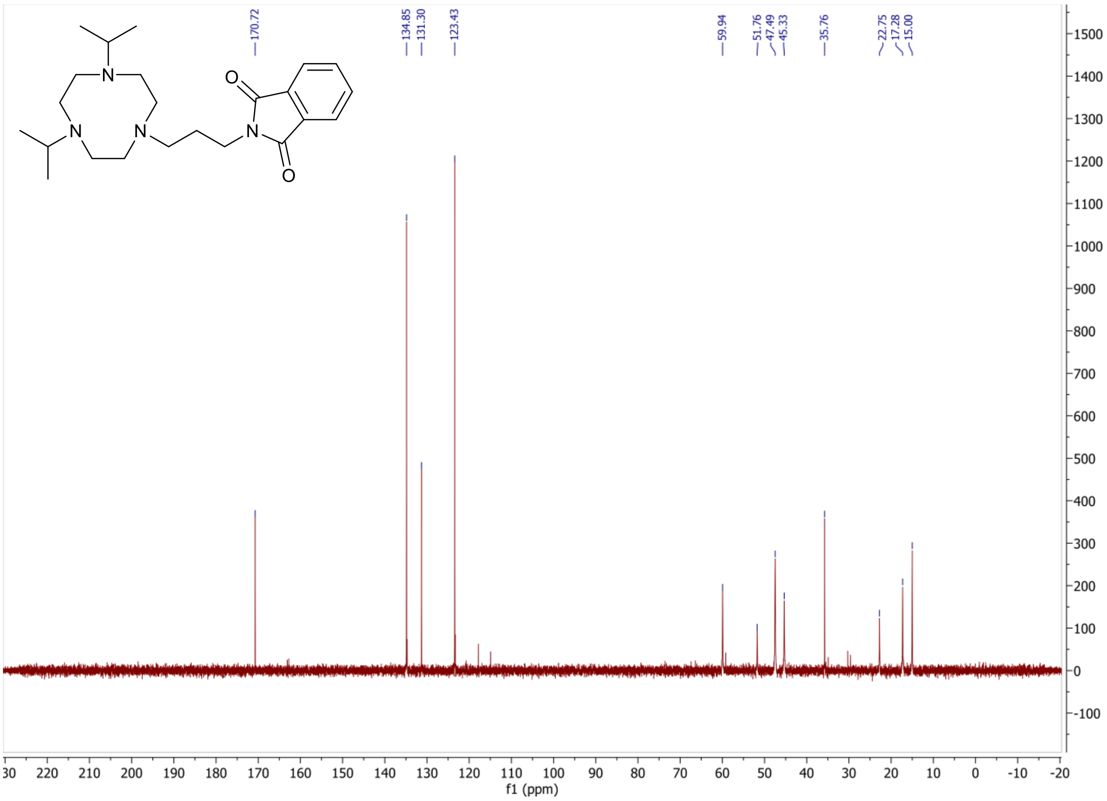

$^{13}C\{^{1}H\}$ (D_2O)

2b

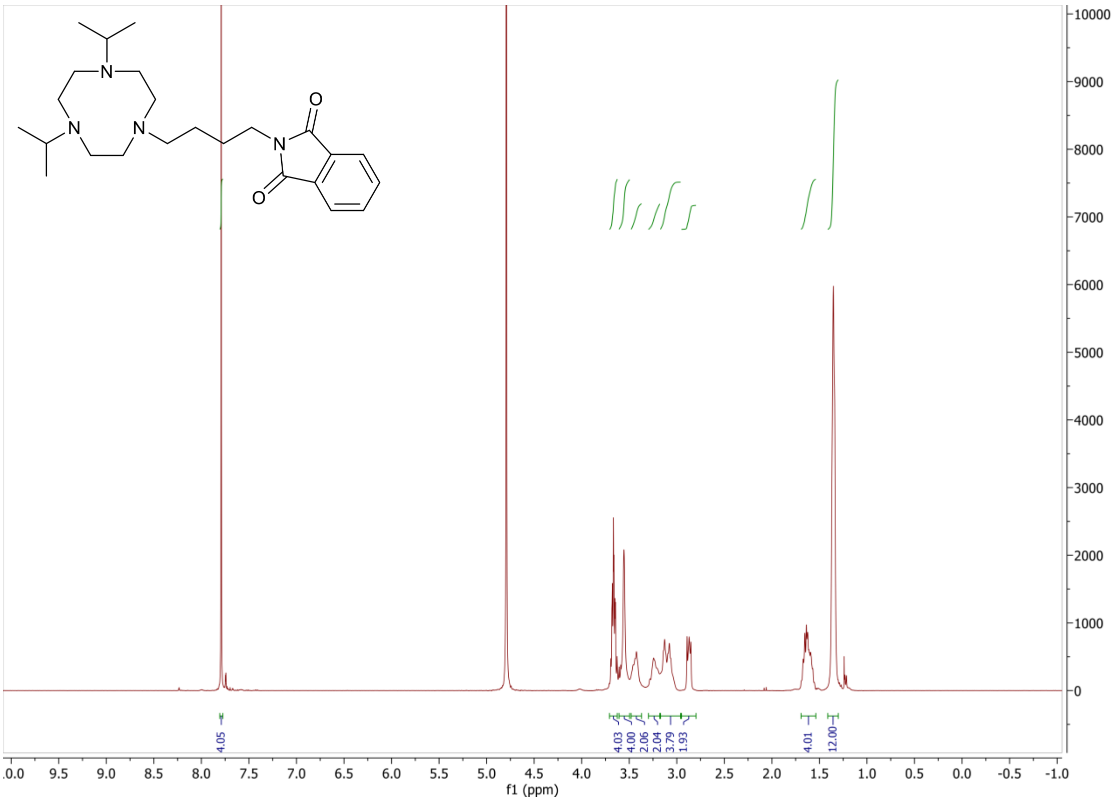

^1H (D_2O)

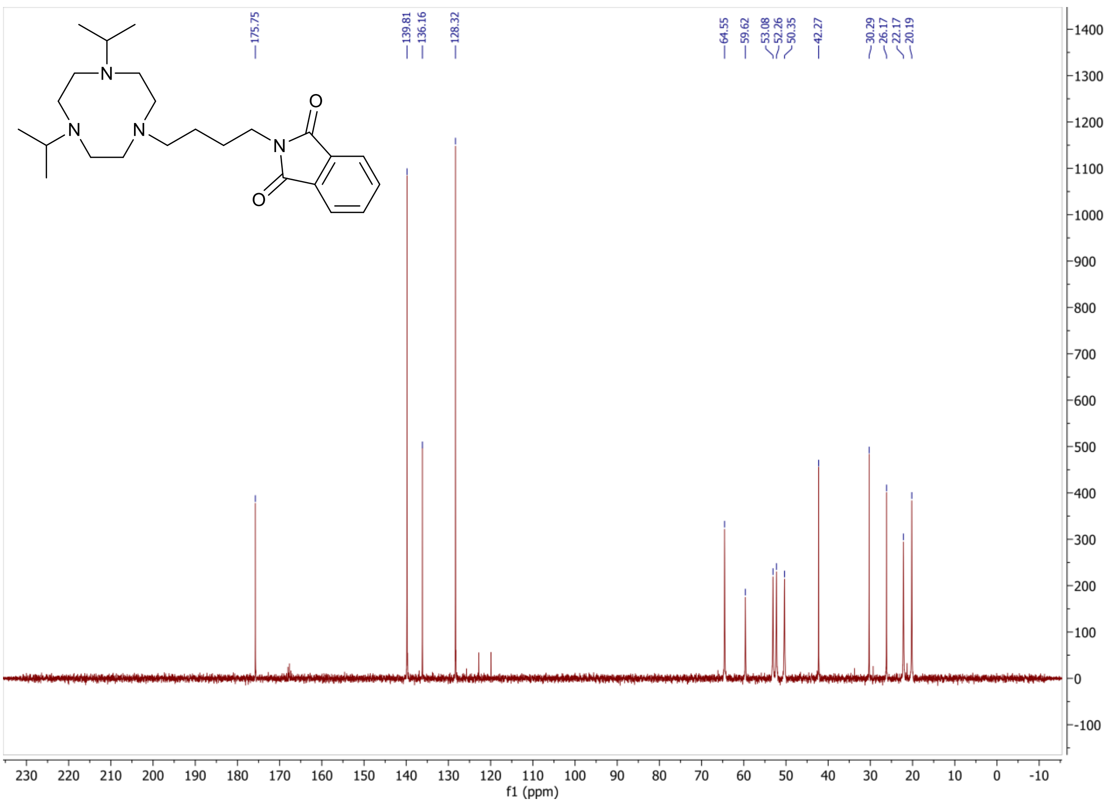

$^{13}C\{^1H\}$ (D_2O)

2c

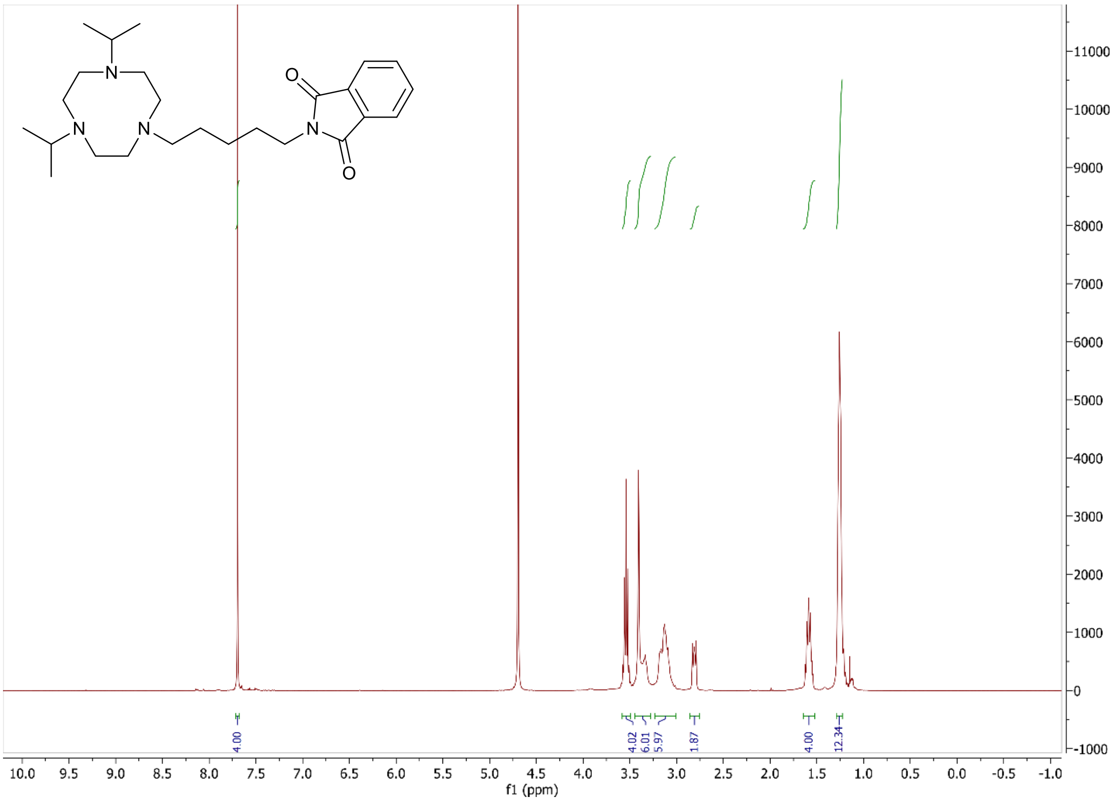

^{1}H (D_2O)

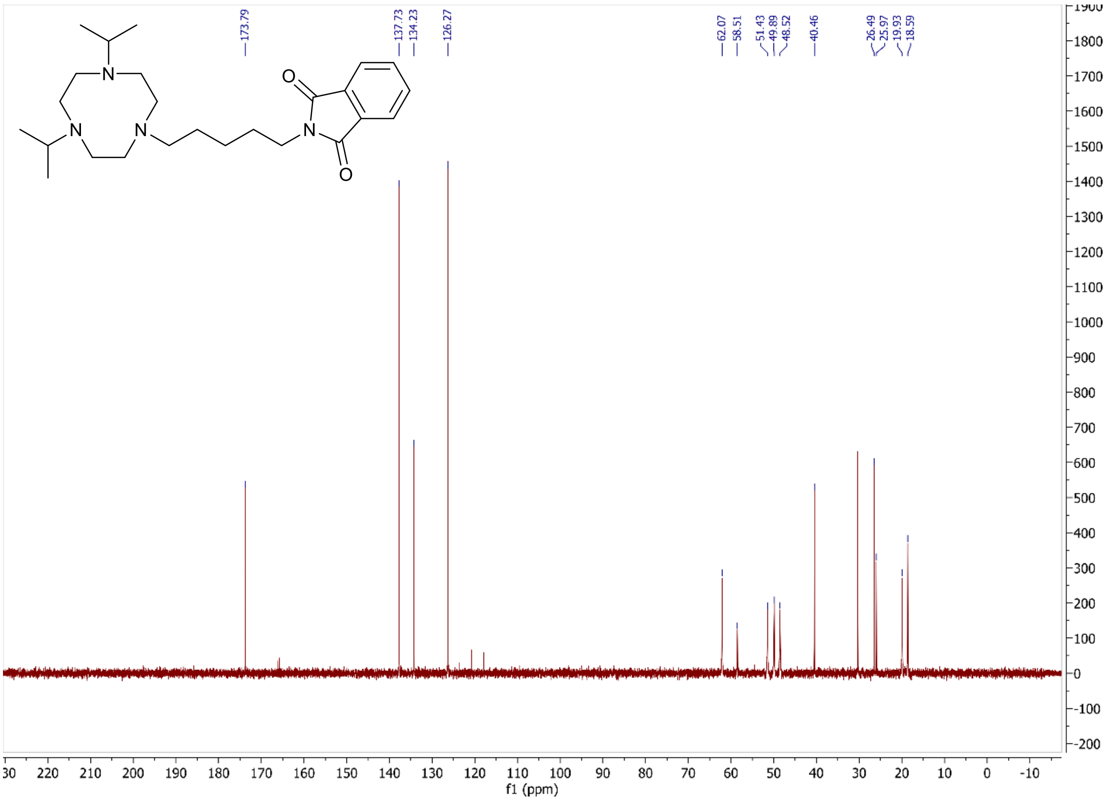

$^{13}C\{^{1}H\}$ (D_2O)

2a

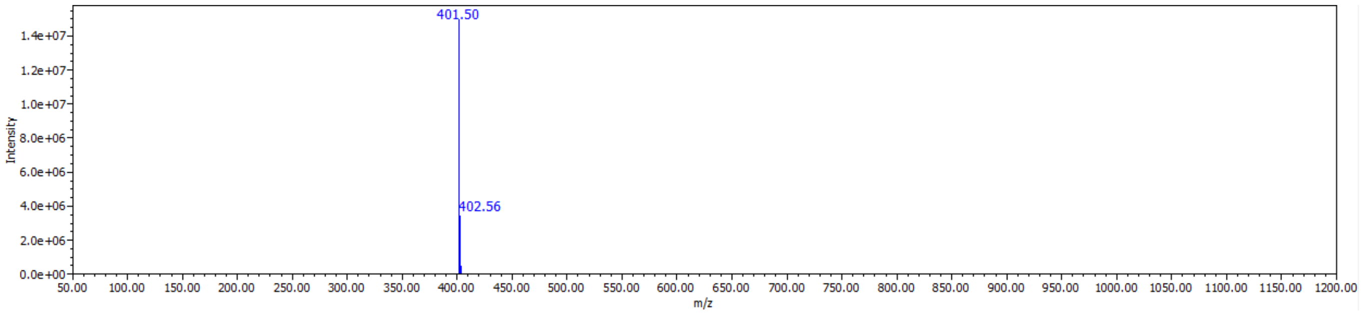

MS-ESI

2b

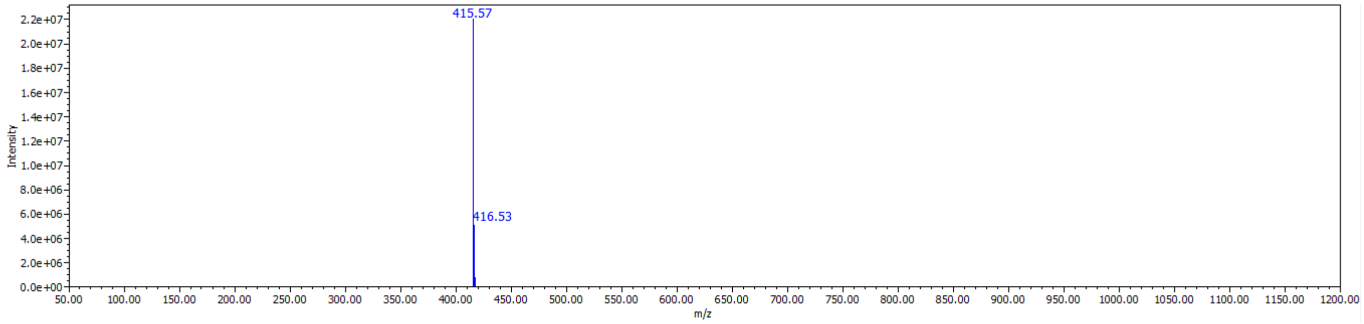

MS-ESI

2c

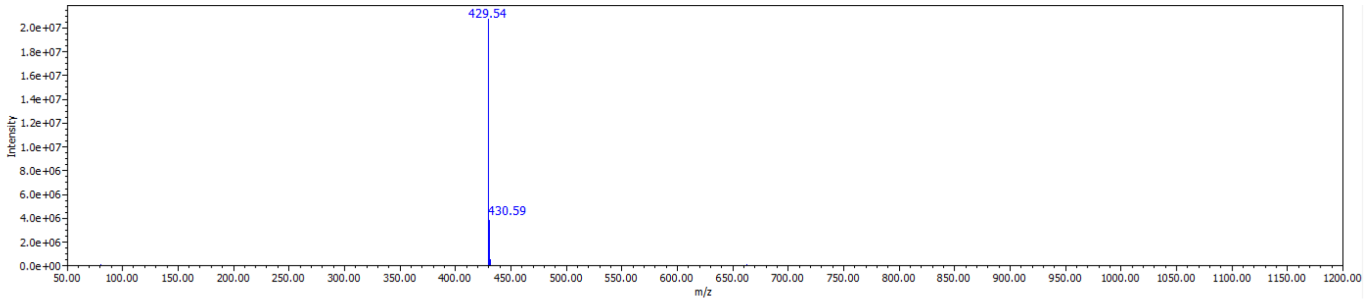

MS-ESI

L10a

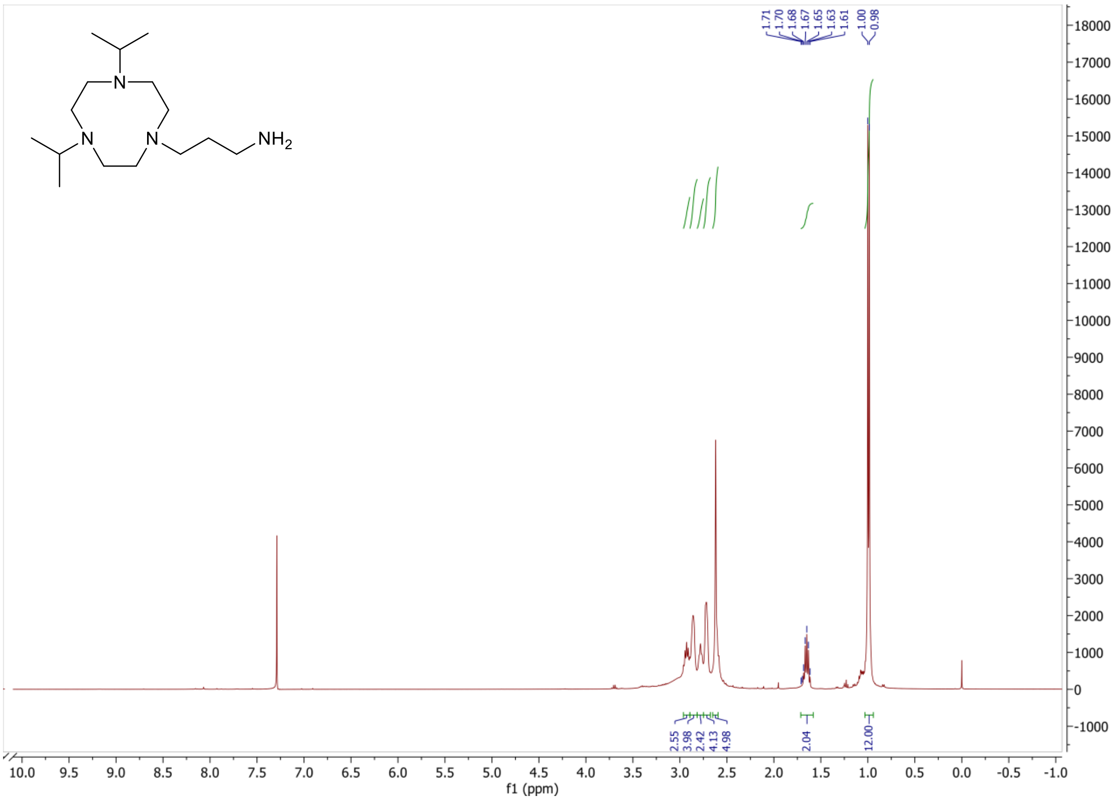

^{1}H ($CDCl_3$)

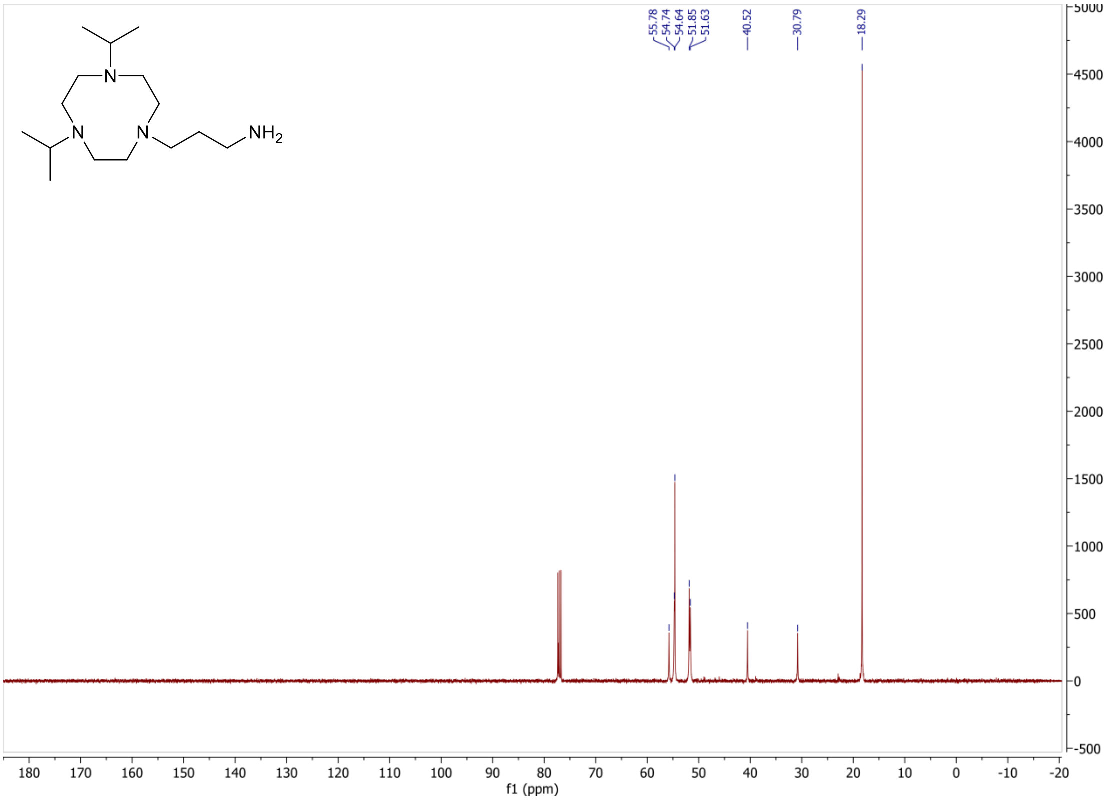

$^{13}C\{^{1}H\}$ ($CDCl_3$)

L10b

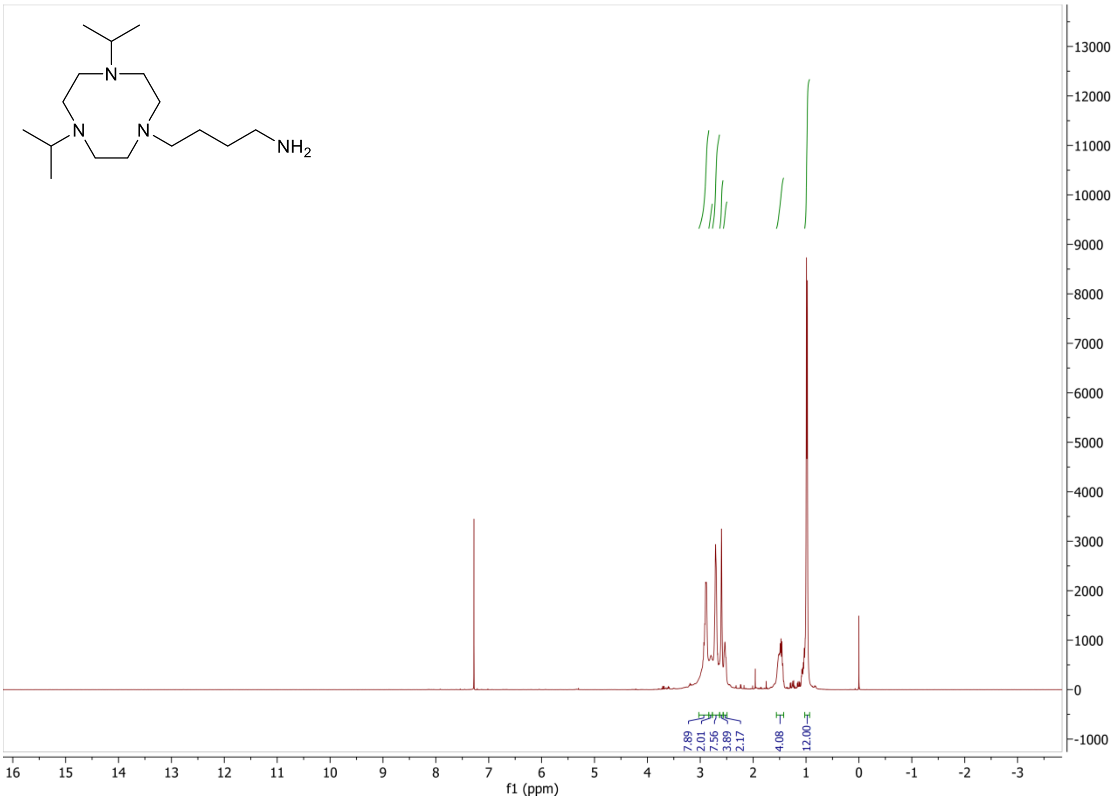

^{1}H ($CDCl_3$)

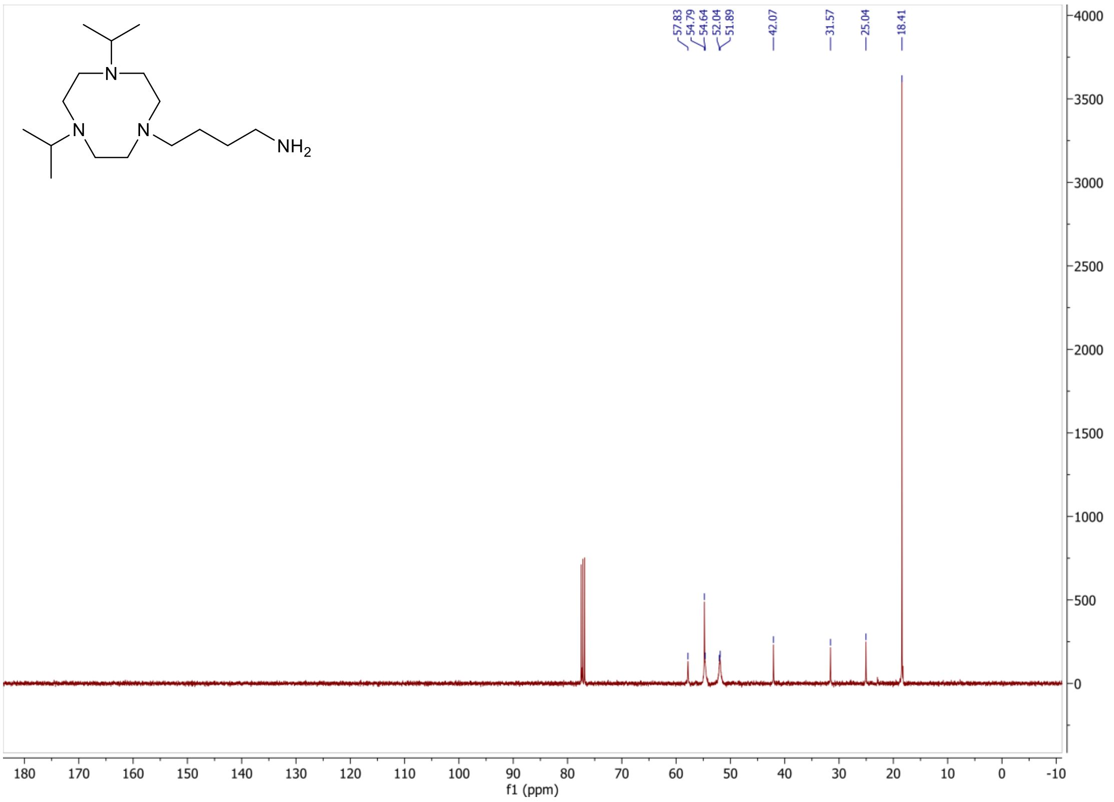

$^{13}C\{^{1}H\}$ ($CDCl_3$)

L10c

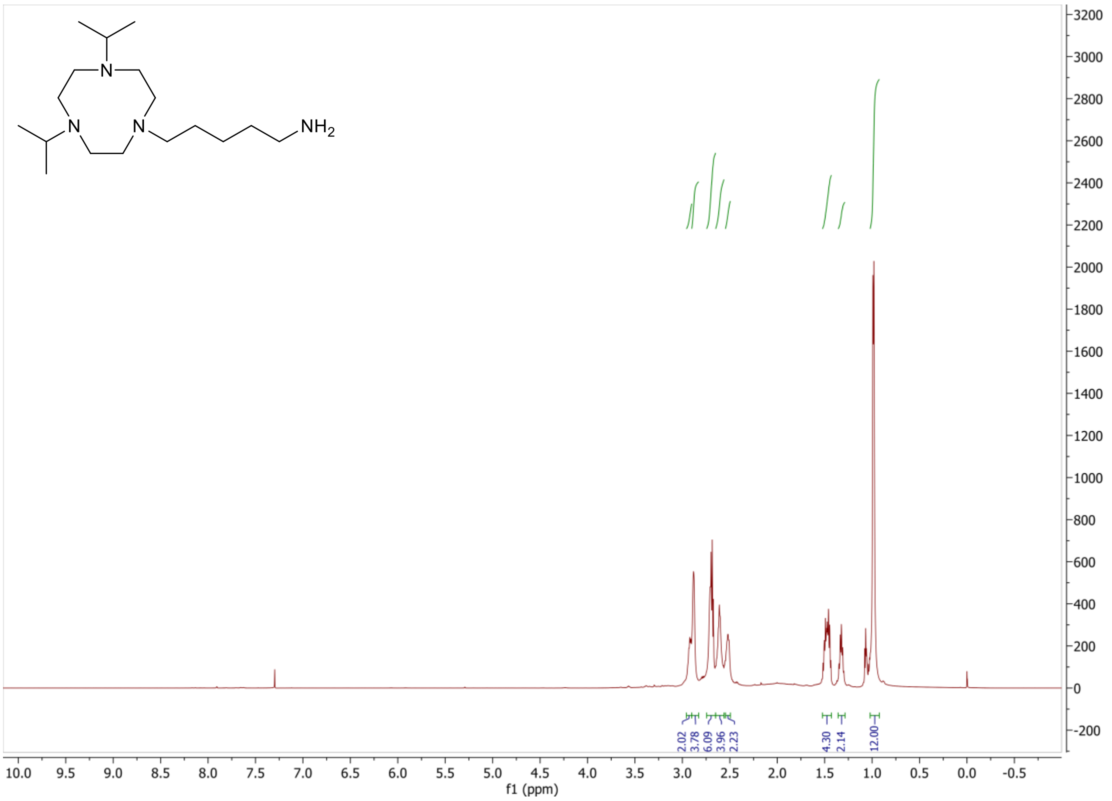

^1H ($CDCl_3$)

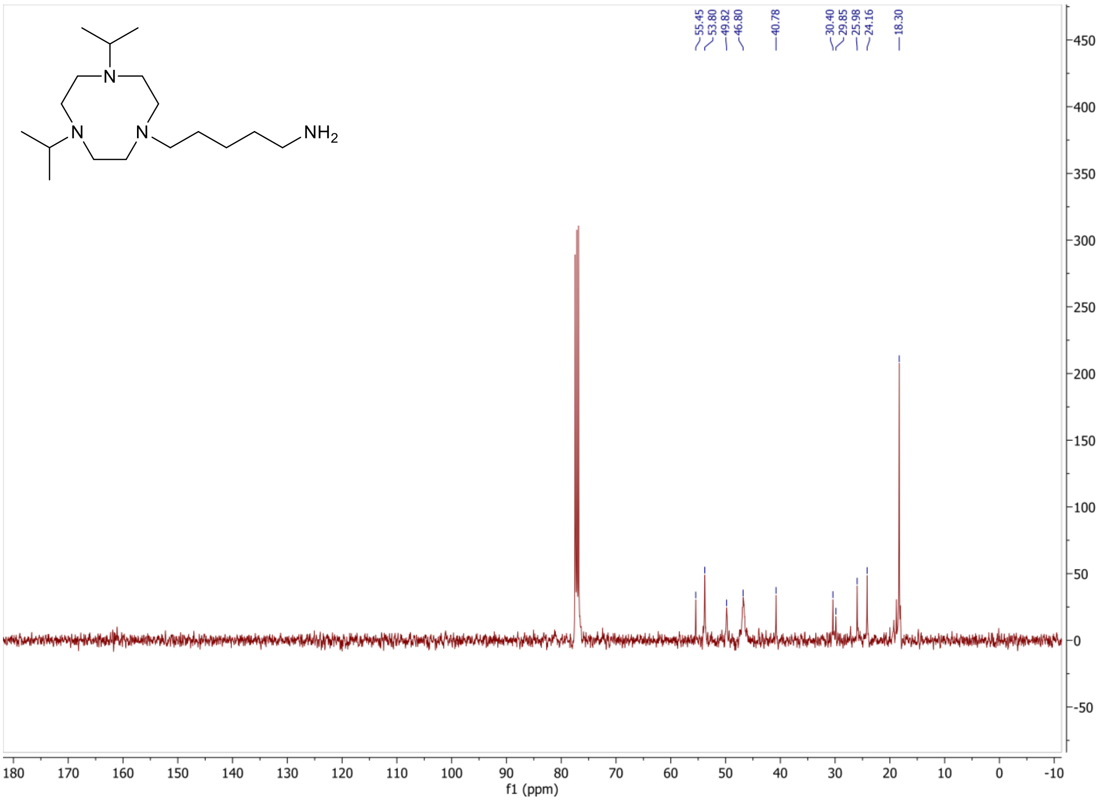

$^{13}C\{^1H\}$ ($CDCl_3$)

L10a

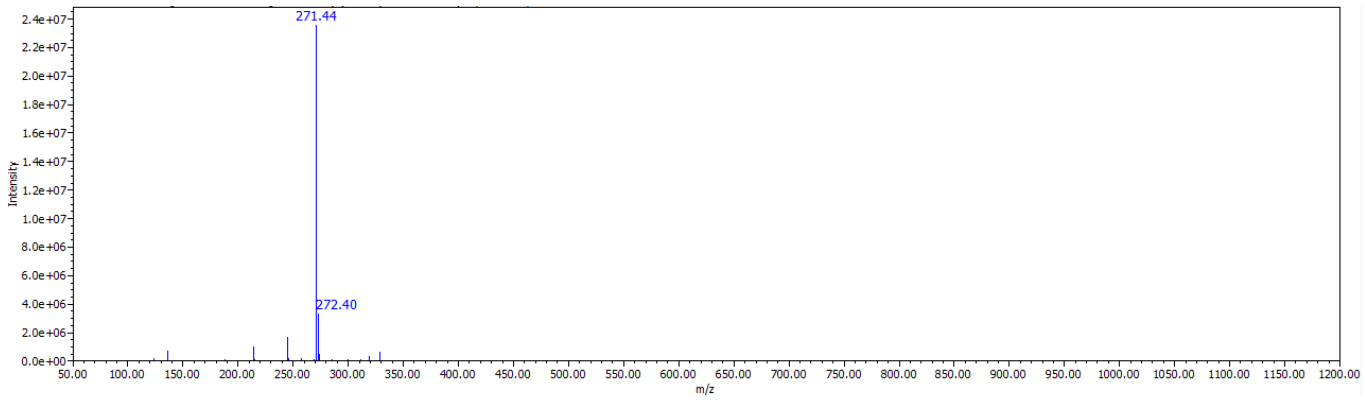

MS-ESI

L10b

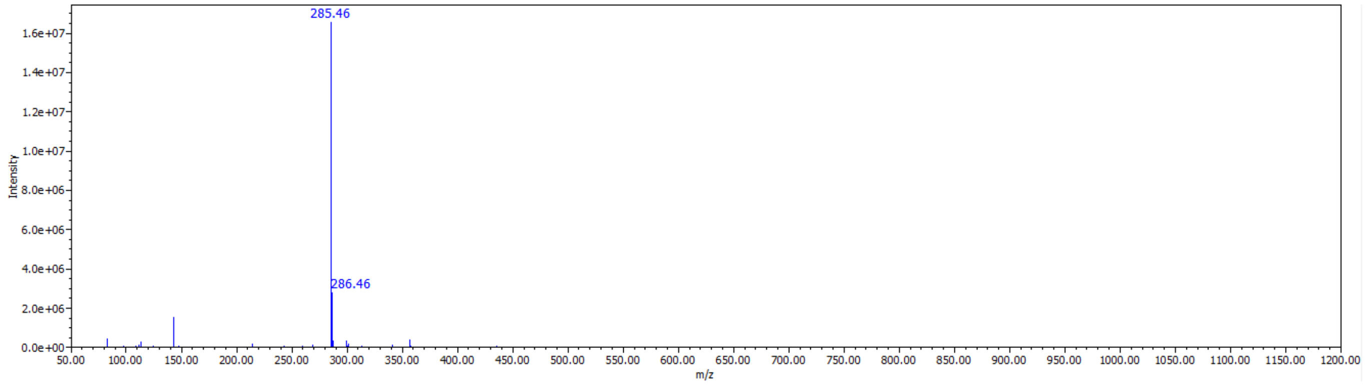

MS-ESI

L10c

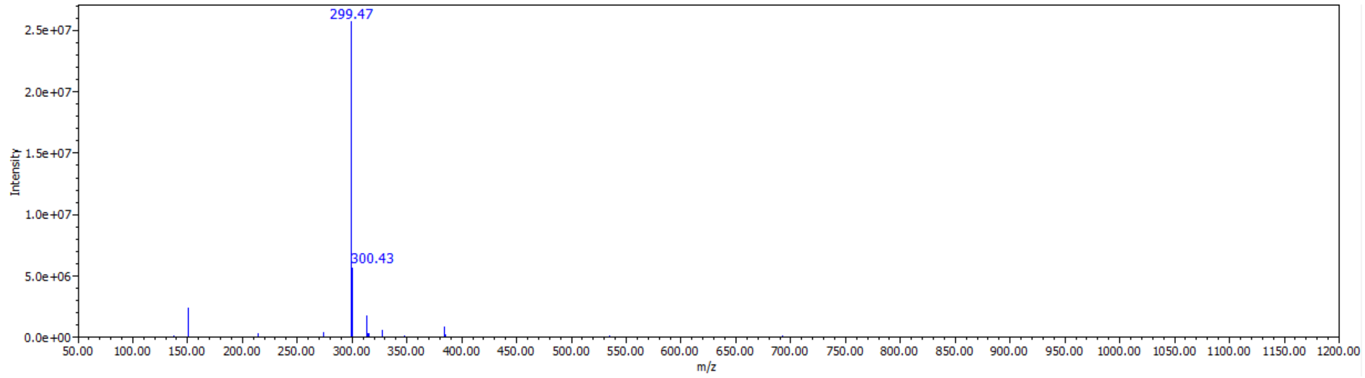

MS-ESI

L11a

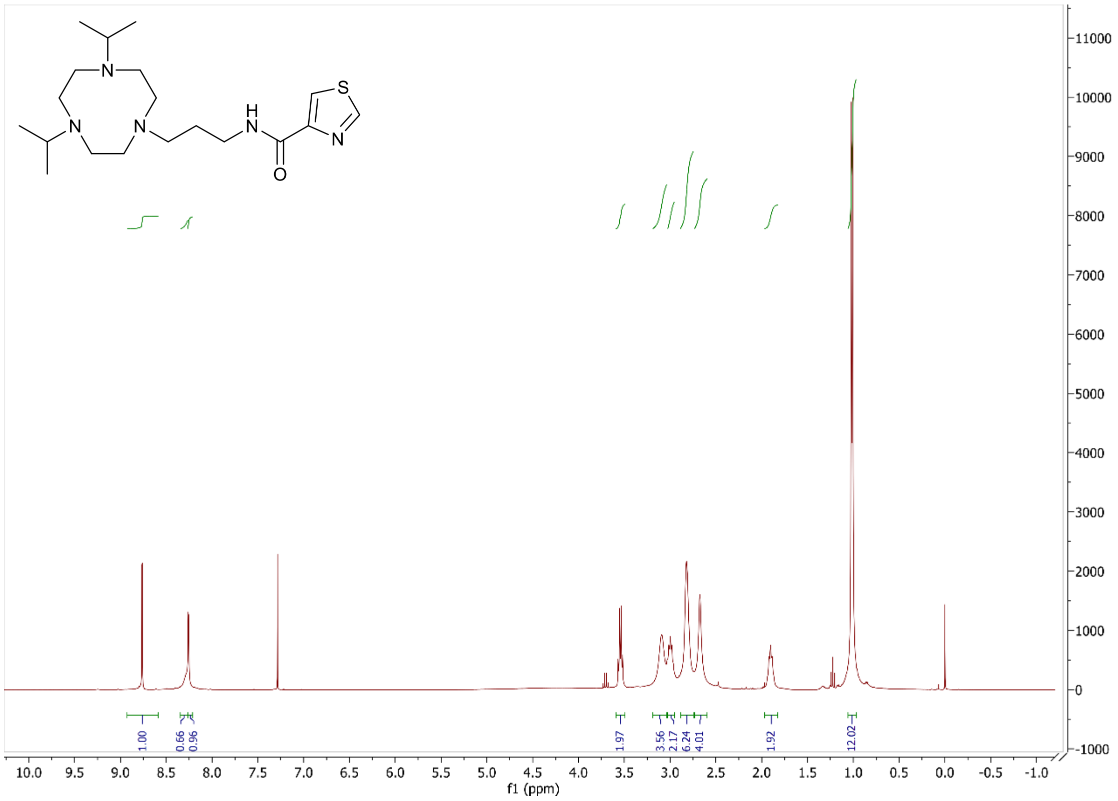

^{1}H ($CDCl_3$)

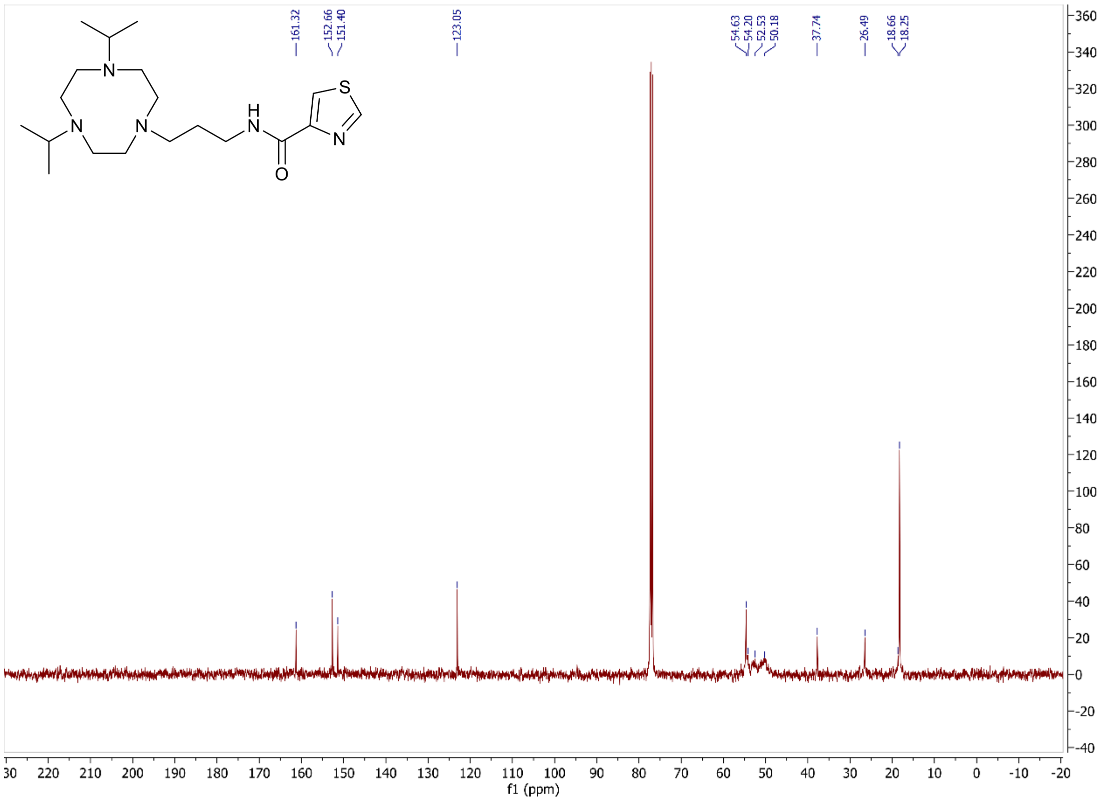

$^{13}C\{^{1}H\}$ ($CDCl_3$)

L11b

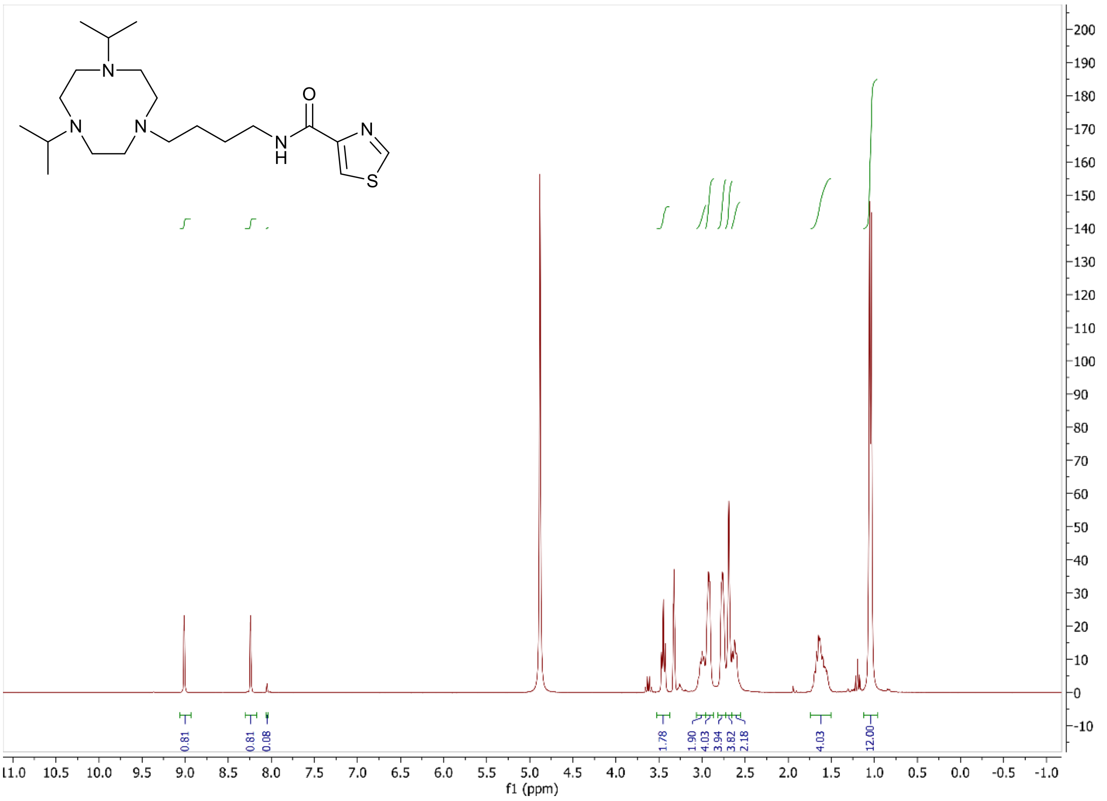

^{1}H (CD_3OD)

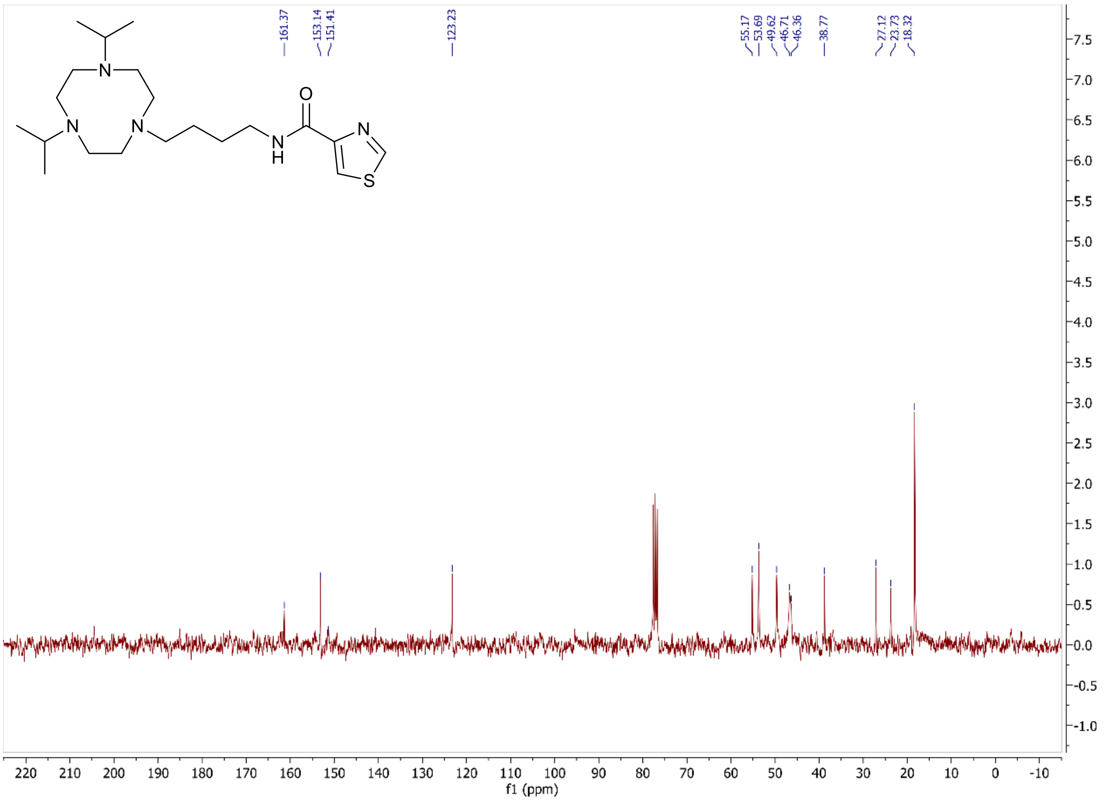

$^{13}C\{^{1}H\}$ ($CDCl_3$)

L11c

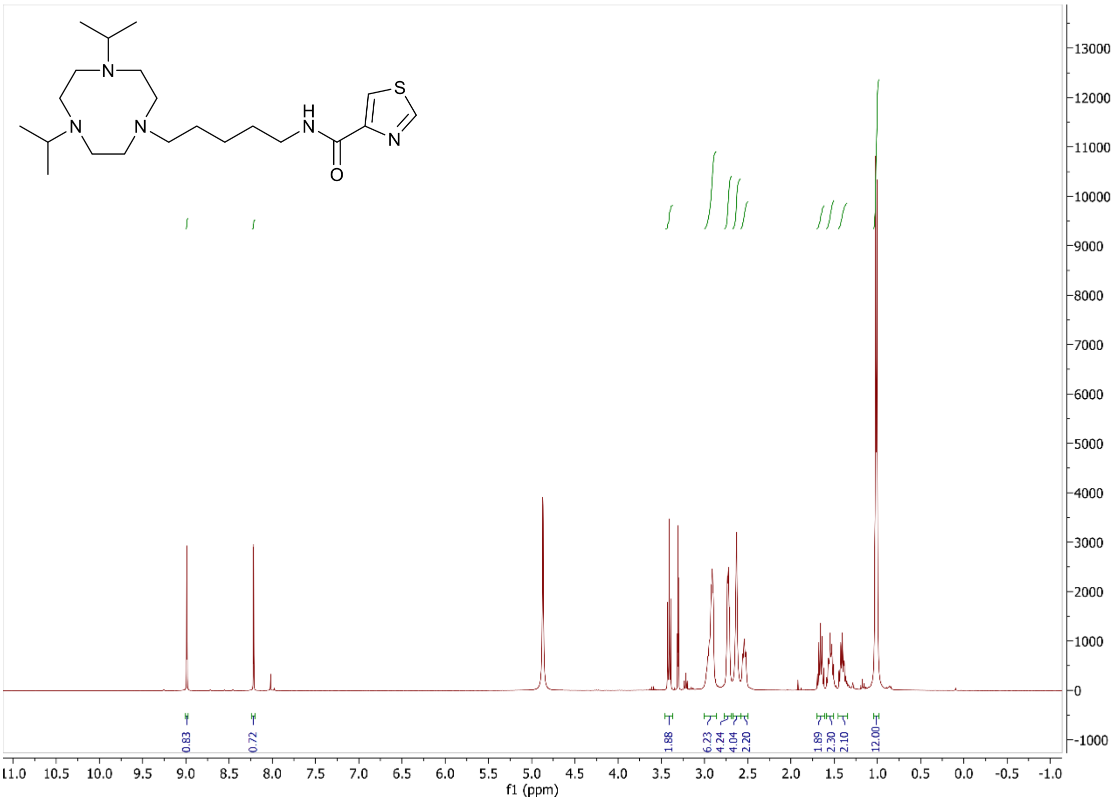

[1]H (CD_3OD)

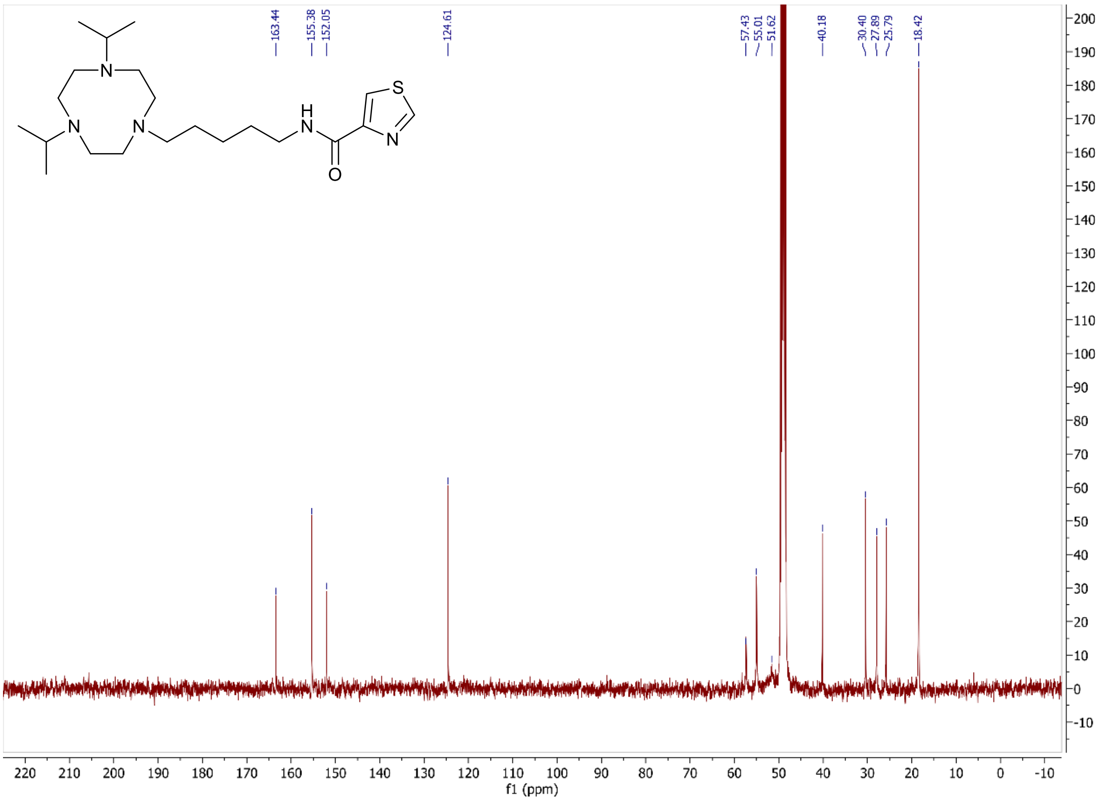

[13]C{[1]H} (CD_3OD)

L11a

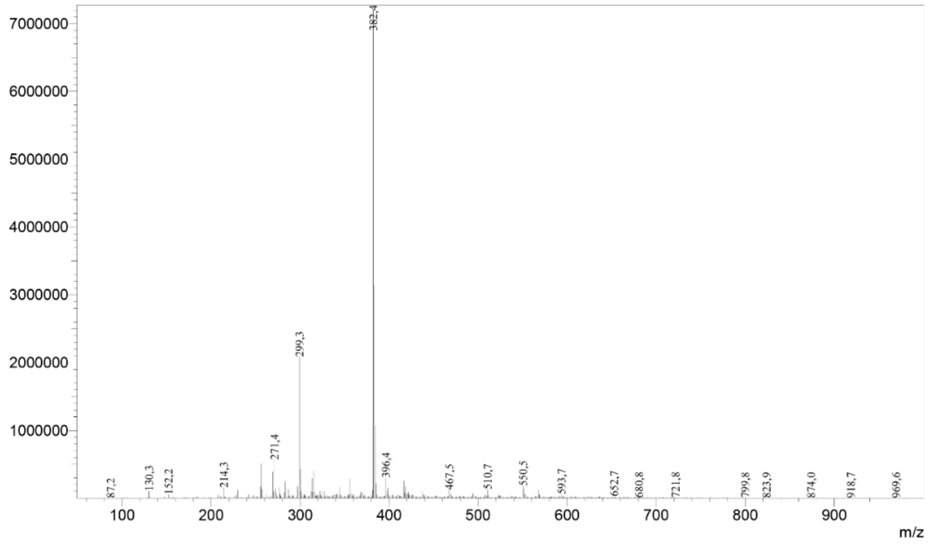

MS-ESI

L11b

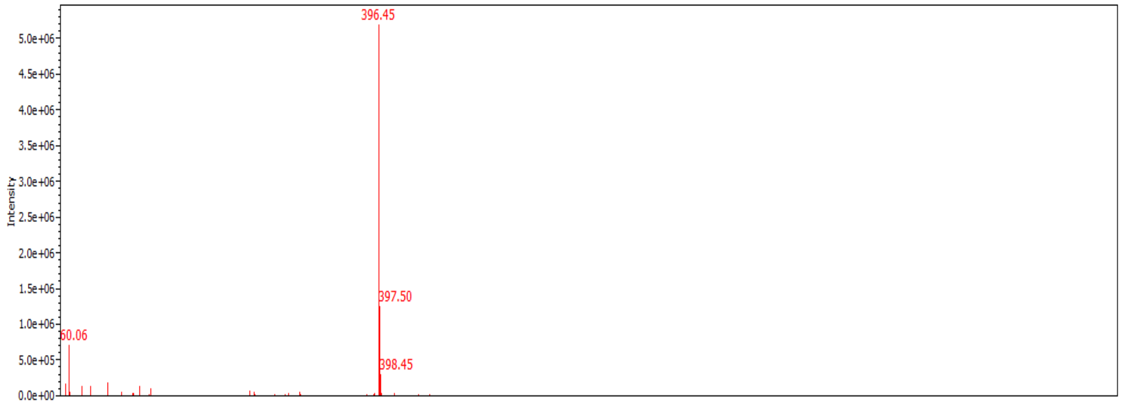

MS-ESI

L11c

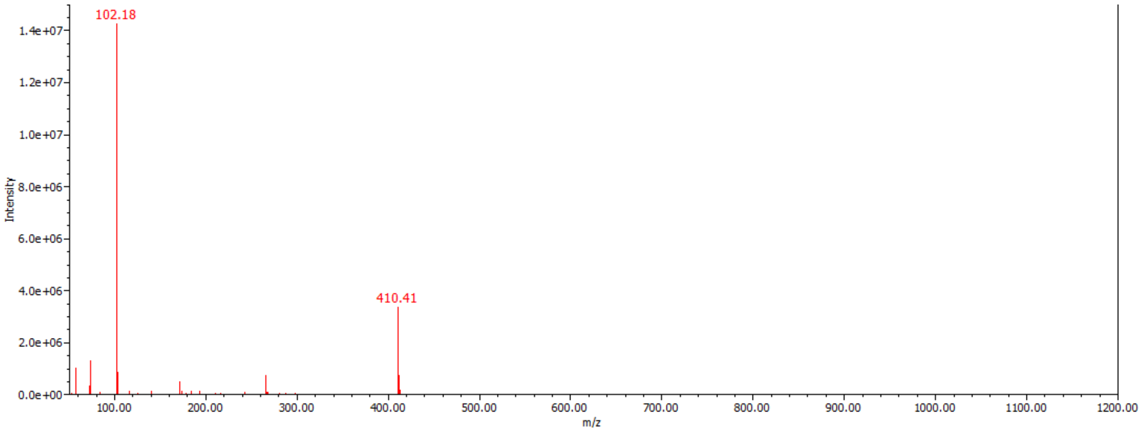

MS-ESI

5

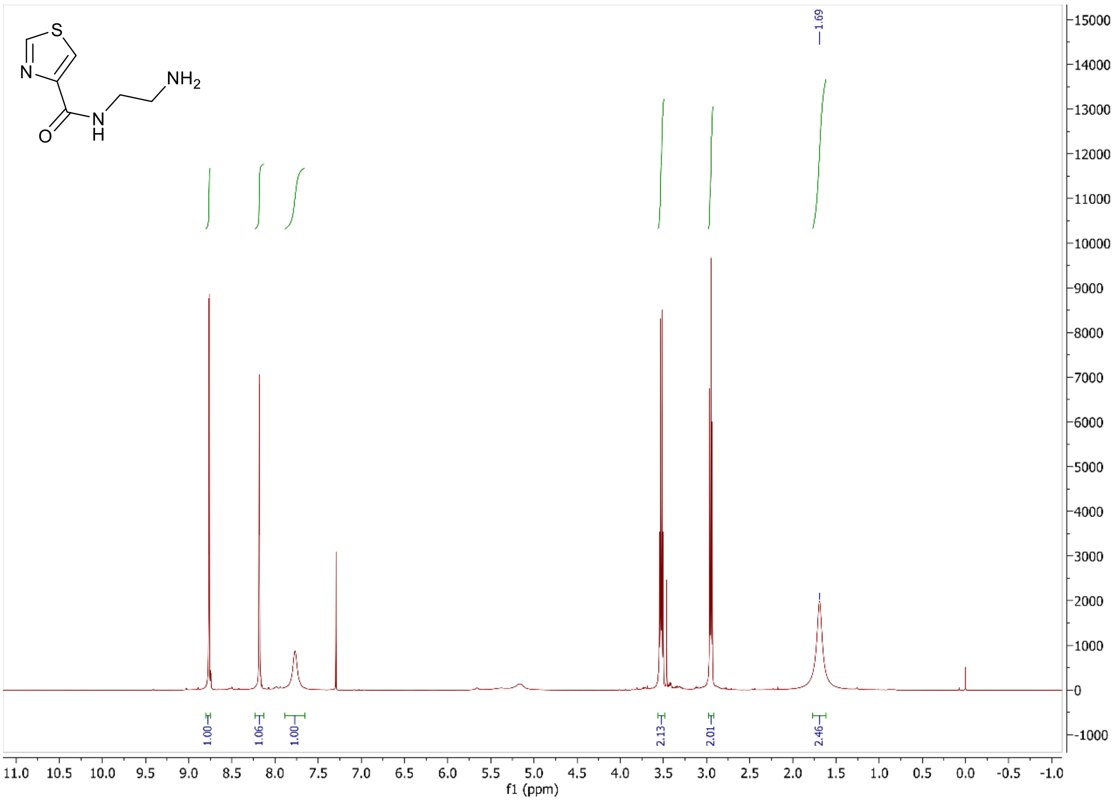

^{1}H (CDCl$_3$)

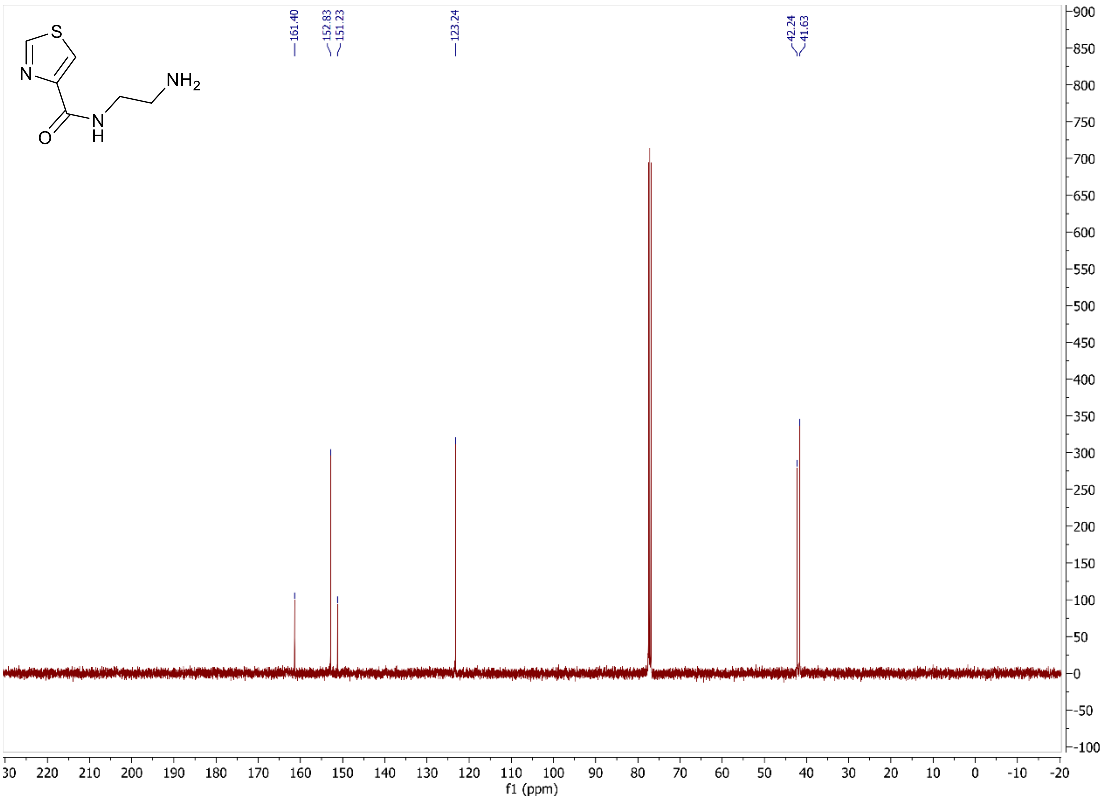

$^{13}C\{^{1}H\}$ (CDCl$_3$)

5

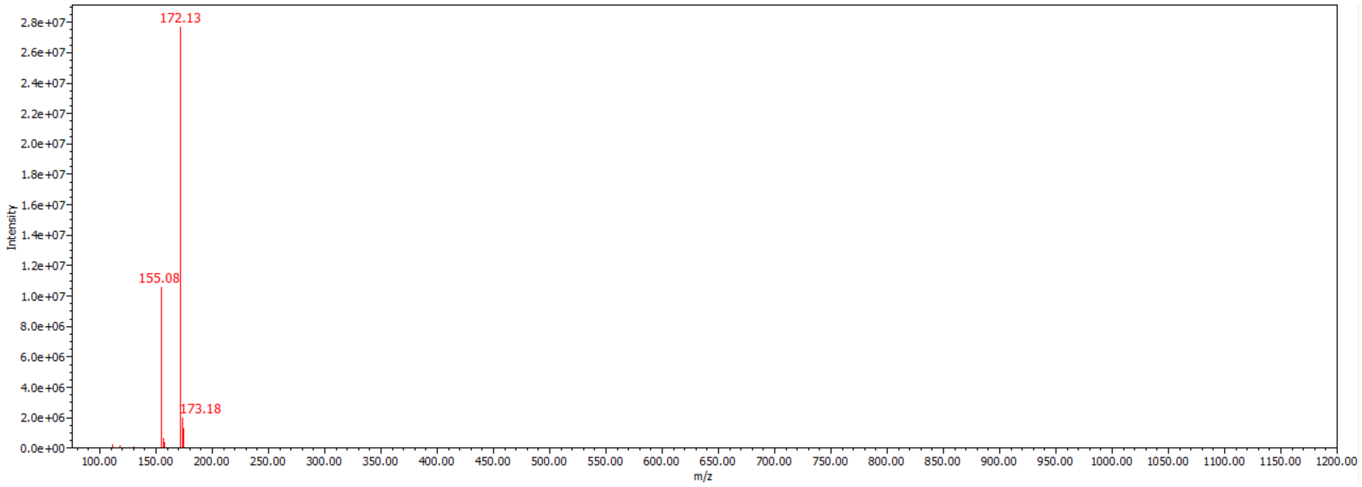

MS-ESI

6a

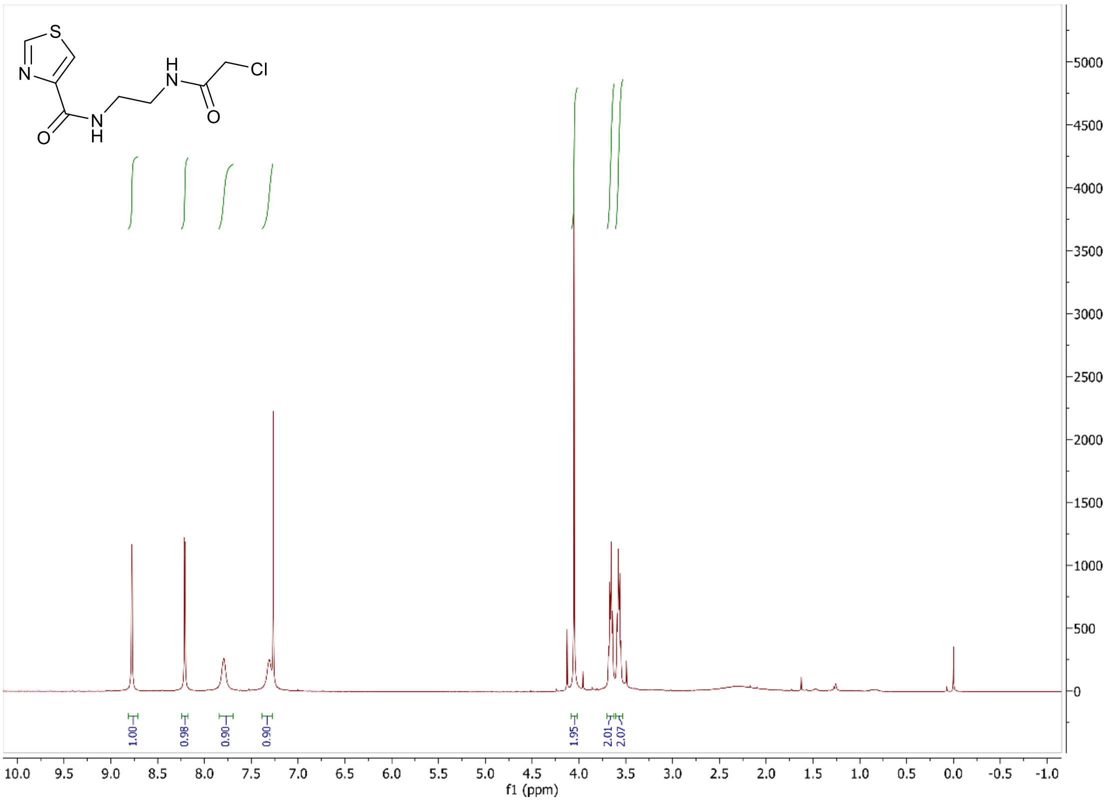

^{1}H (CDCl$_3$)

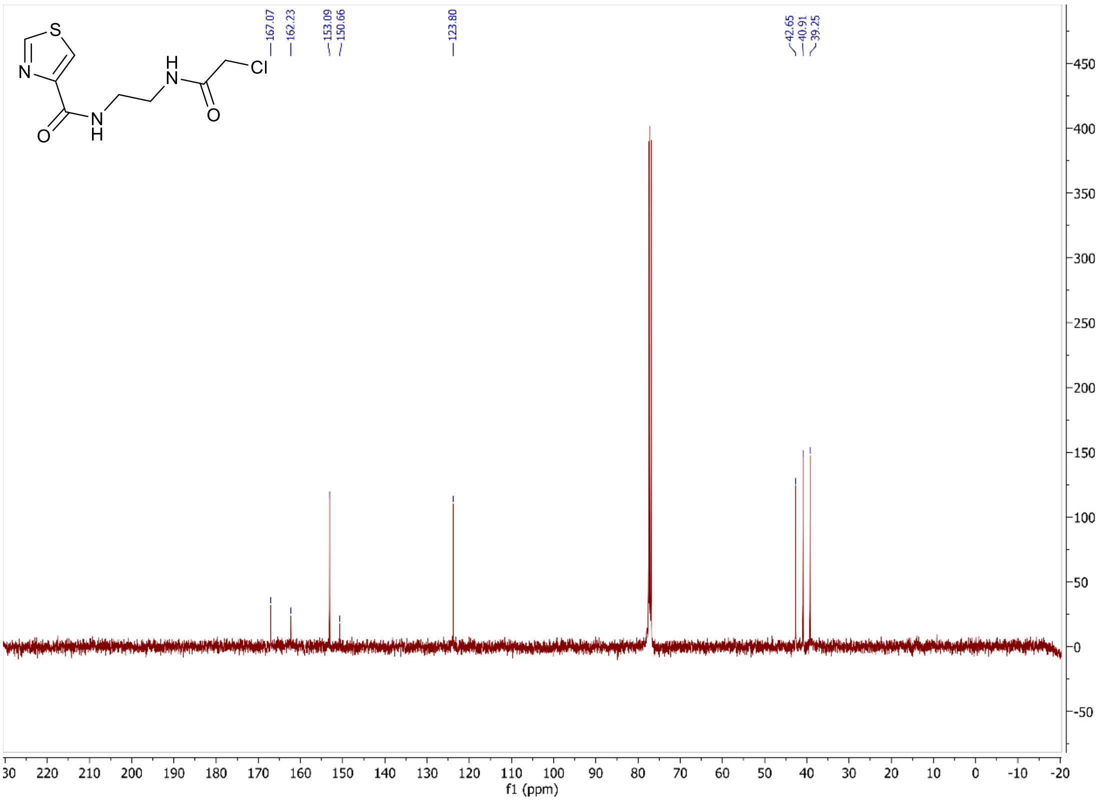

$^{13}C\{^{1}H\}$ (CDCl$_3$)

6b

70% purity due to formation of vinyl species; used in this state for next reaction step, as the impurity does not react as an alkylating reagent.

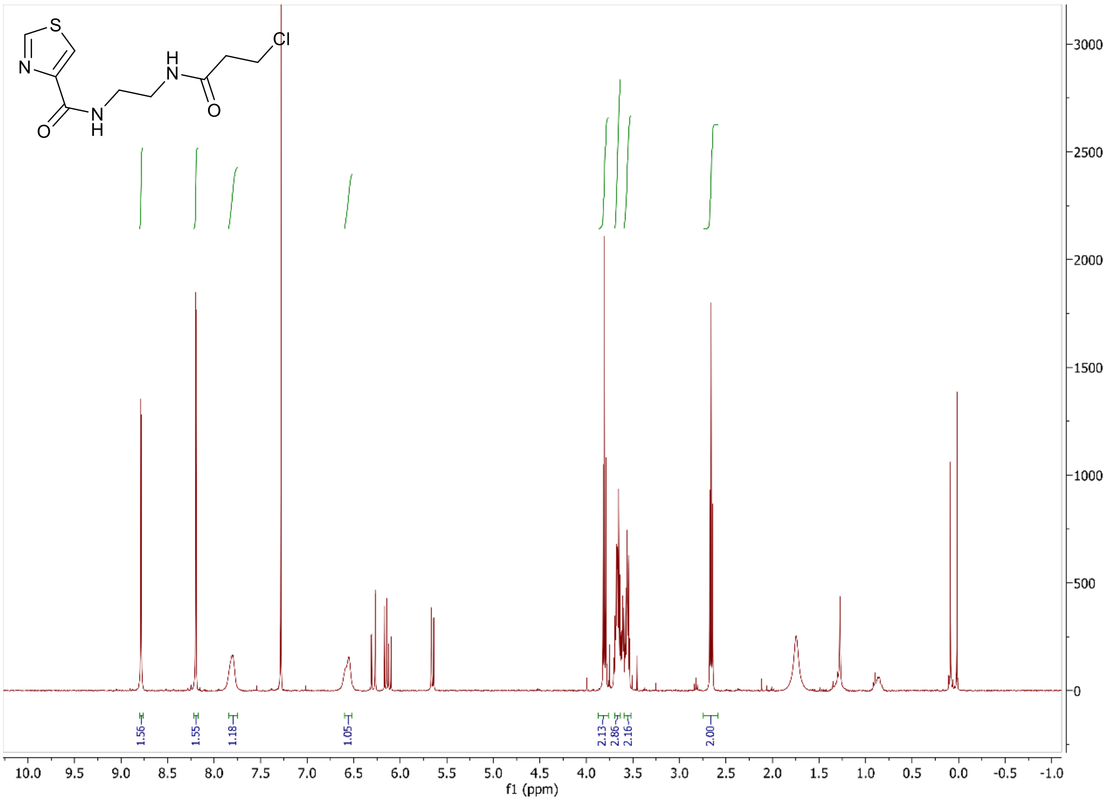

^{1}H (CDCl$_3$)

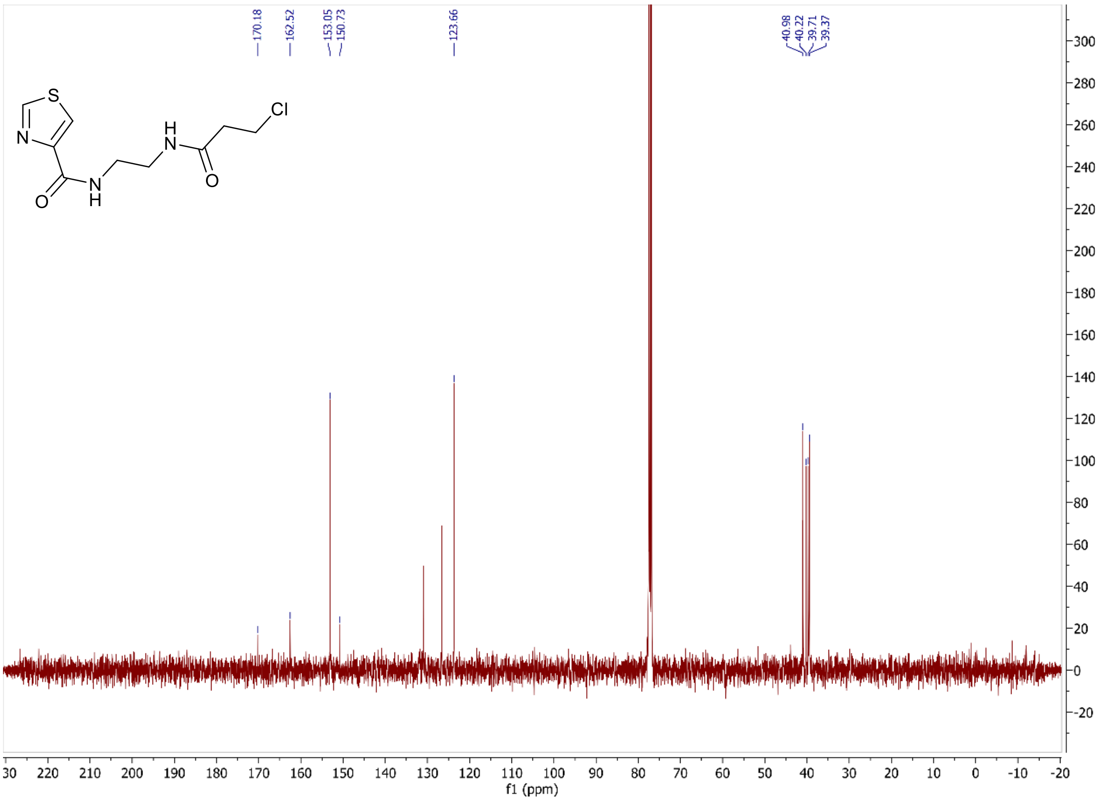

$^{13}C\{^{1}H\}$ (CDCl$_3$)

L12a

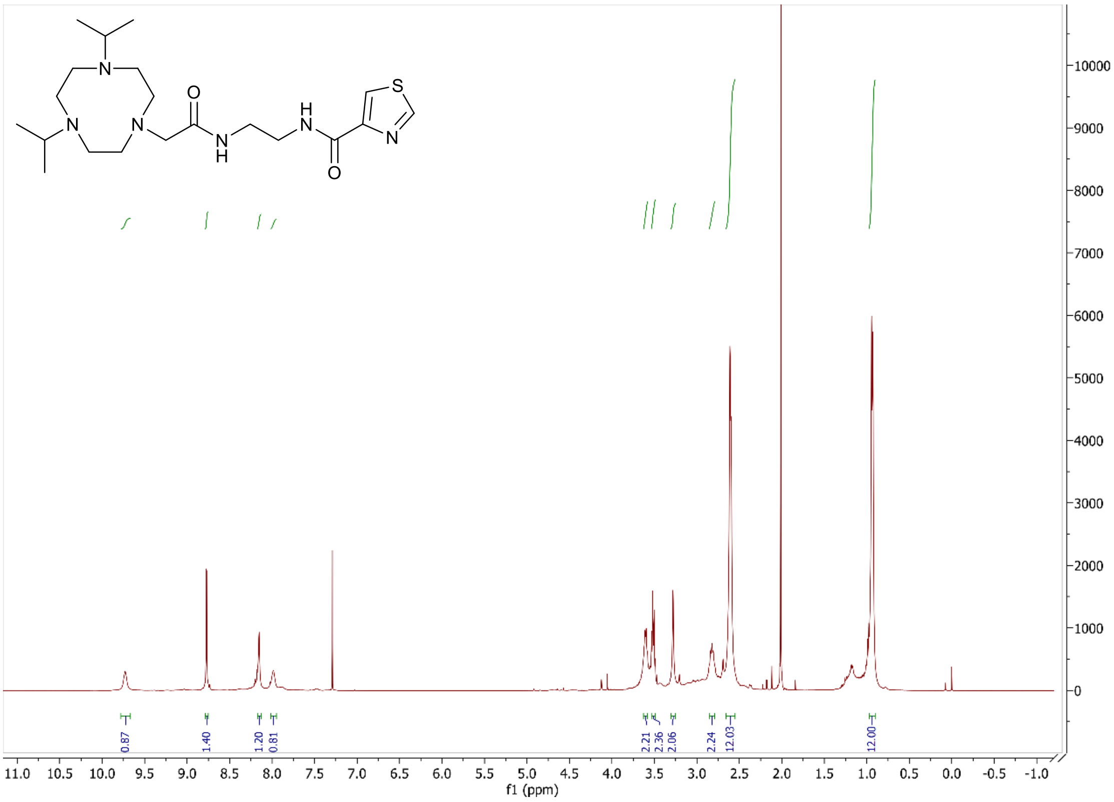

^{1}H ($CDCl_3$)

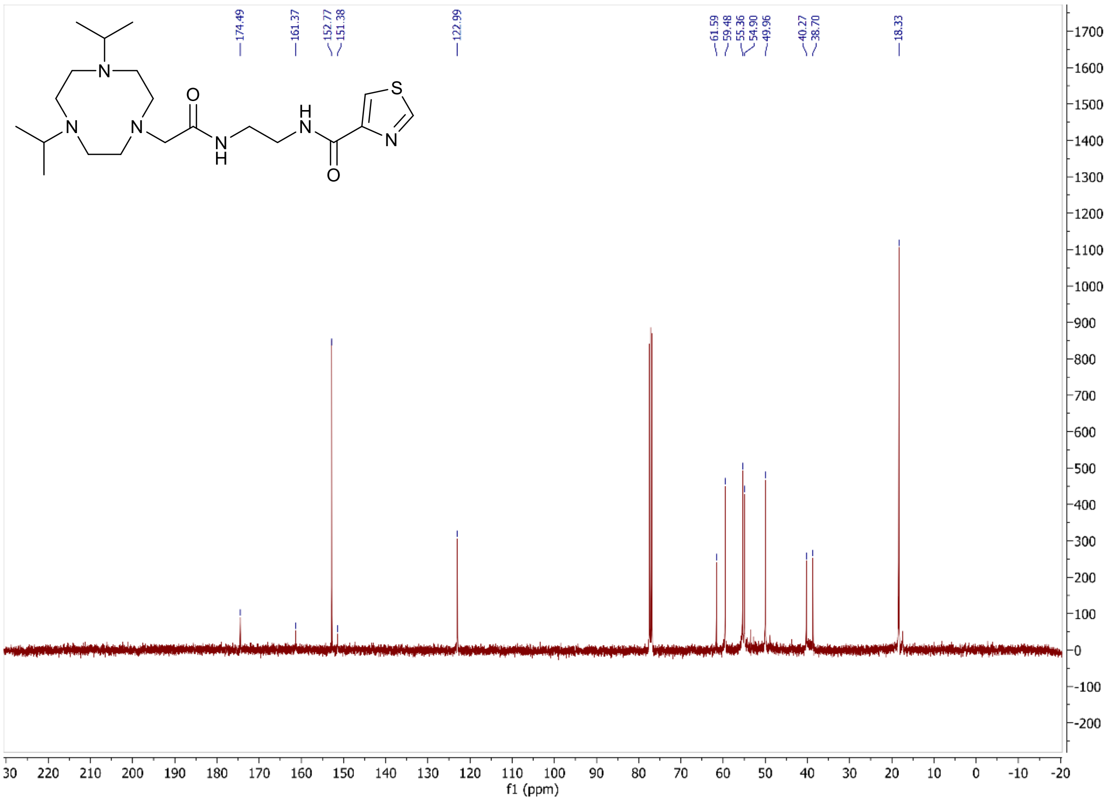

$^{13}C\{^{1}H\}$ ($CDCl_3$)

L12b

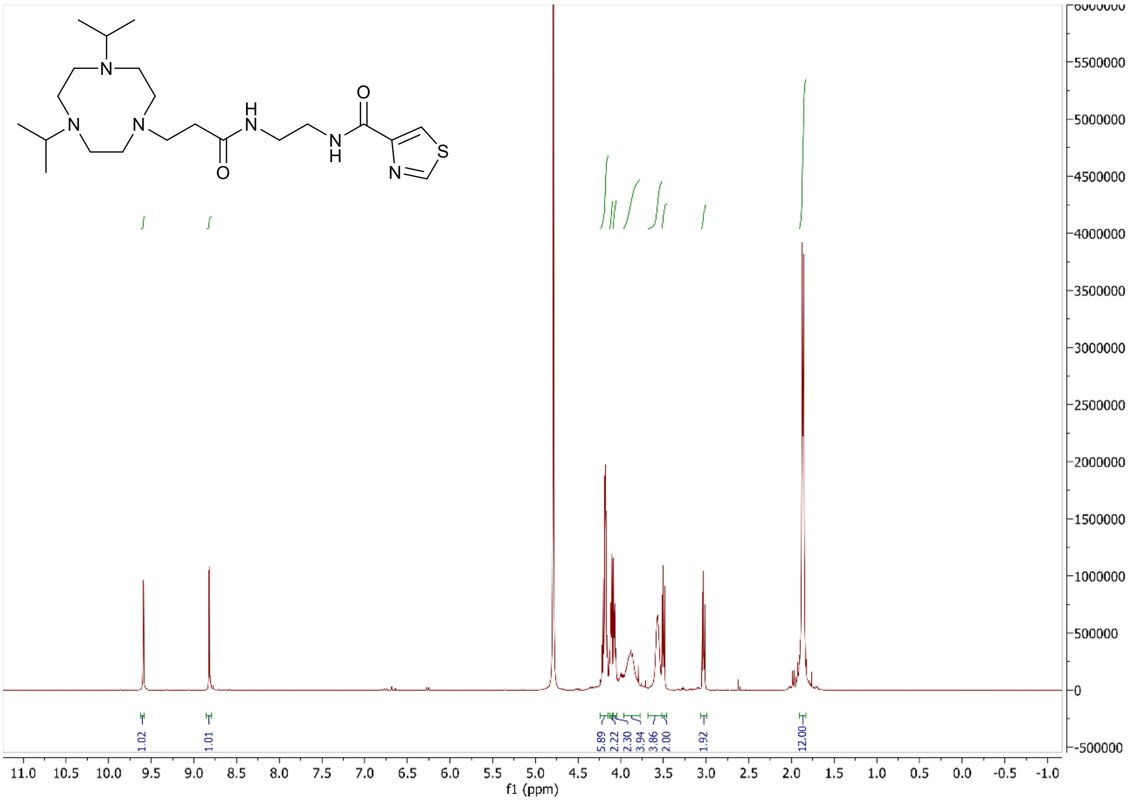

^{1}H (D_2O, 80 °C); spectra obtained at 80 °C because of the amide group being in a frozen conformation at 25 °C.

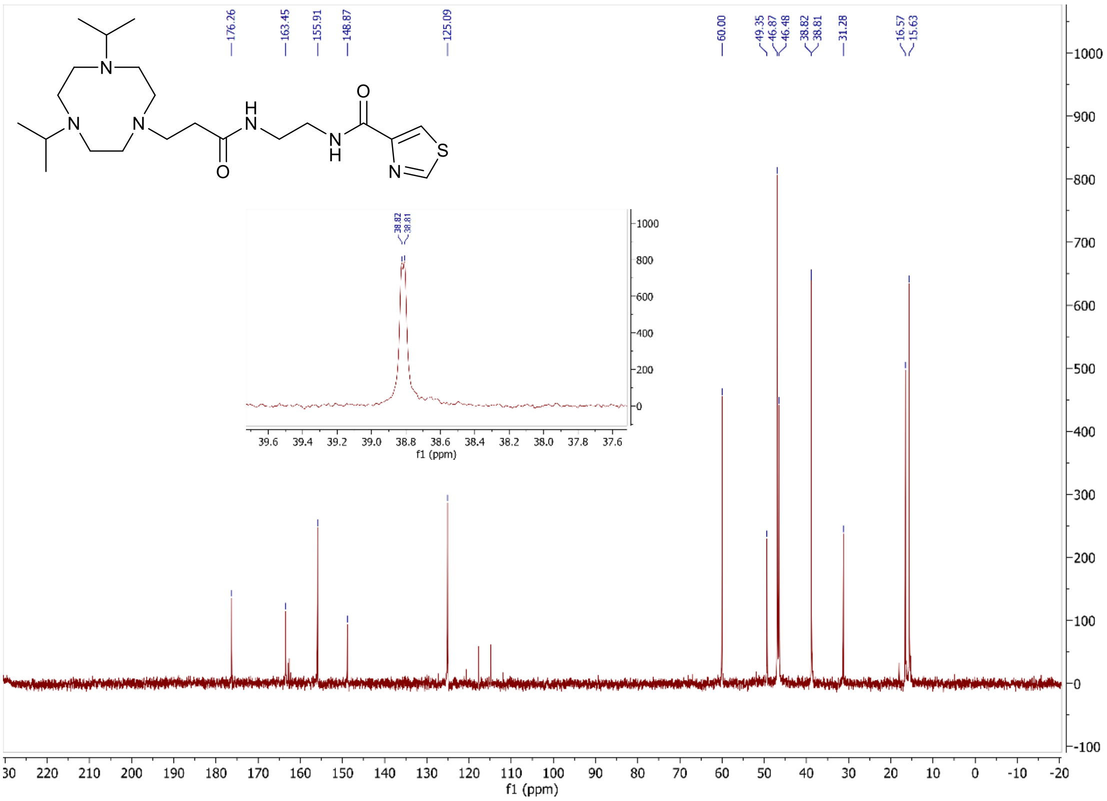

[13]C{[1]H} (D_2O).

L12a

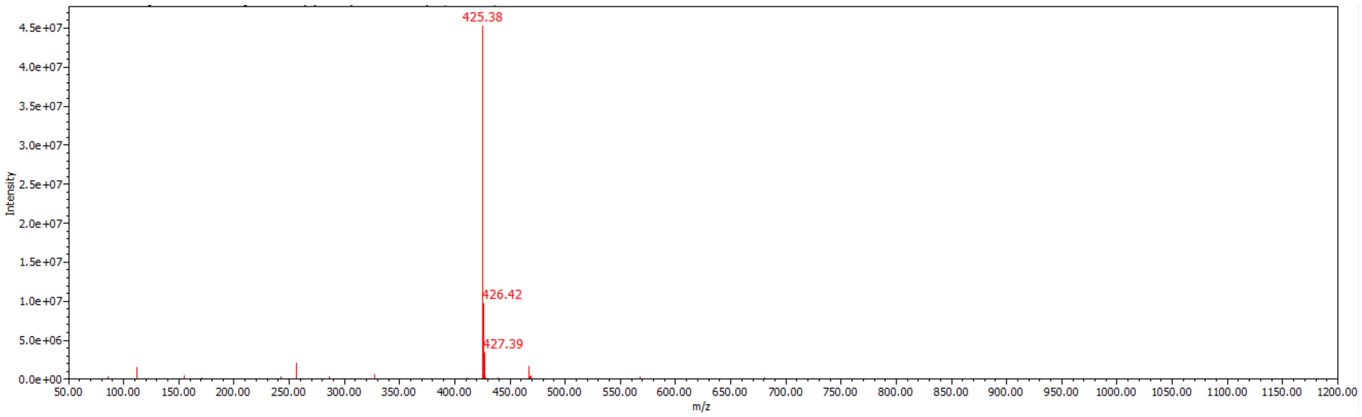

MS-ESI

L12b

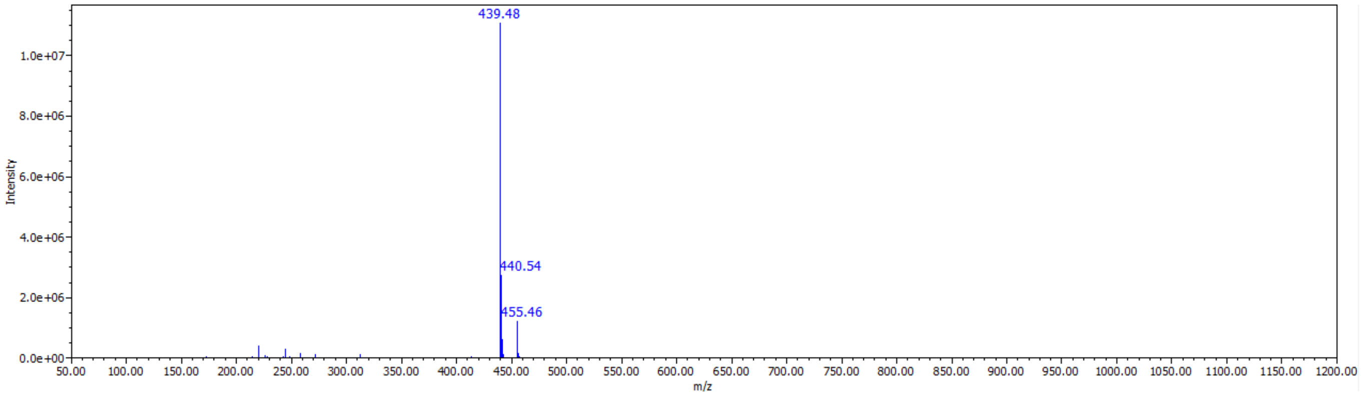

MS-ESI

L13a

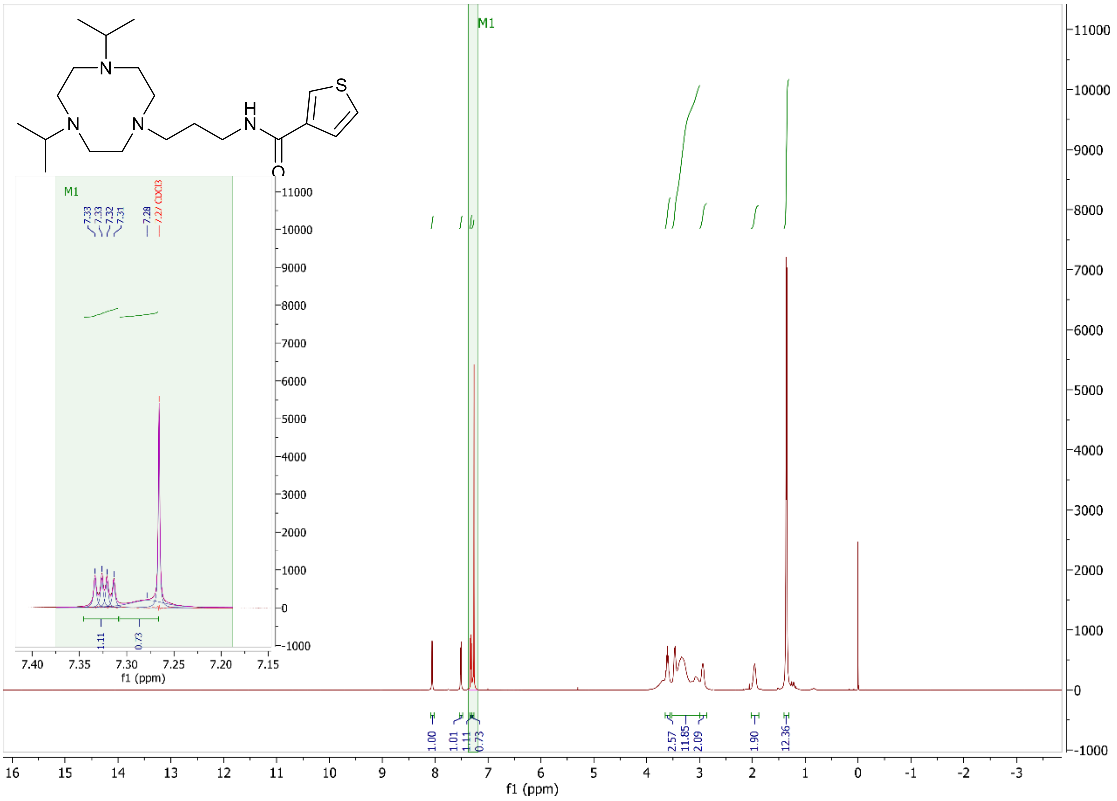

^{1}H (CDCl$_3$)

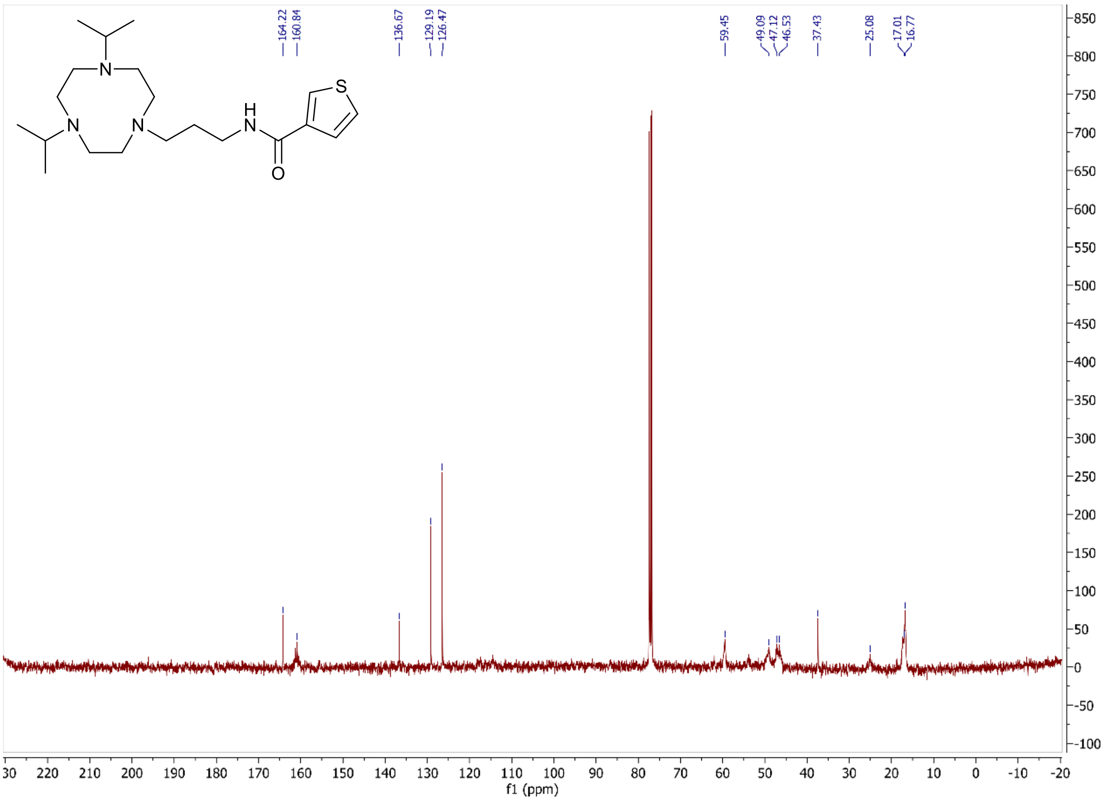

$^{13}C\{^{1}H\}$ (CDCl$_3$)

L13b

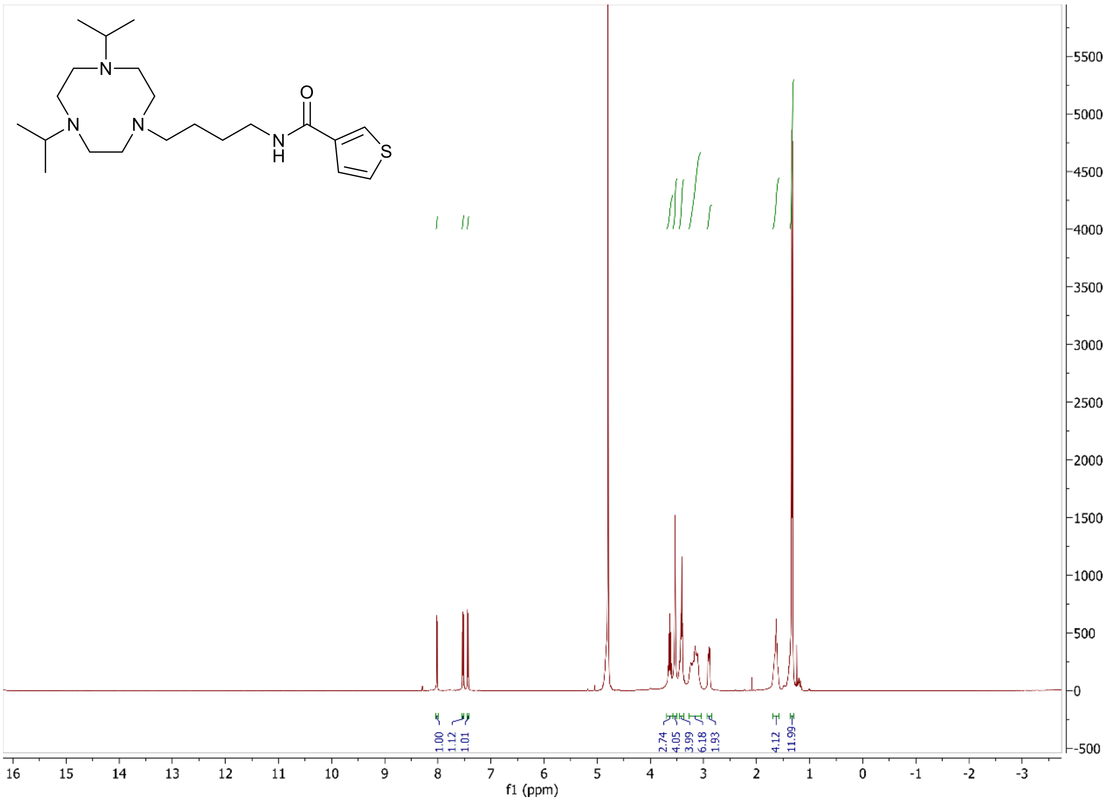

^{1}H (D_2O)

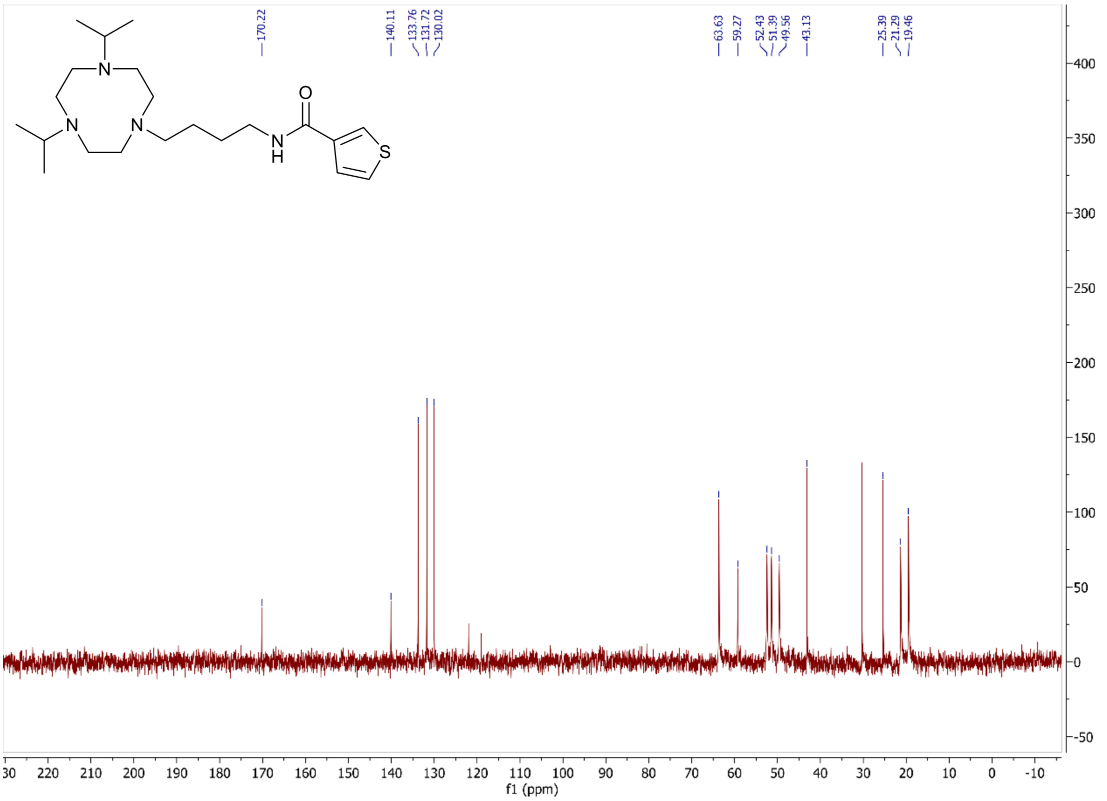

$^{13}C\{^{1}H\}$ (D_2O)

L13c

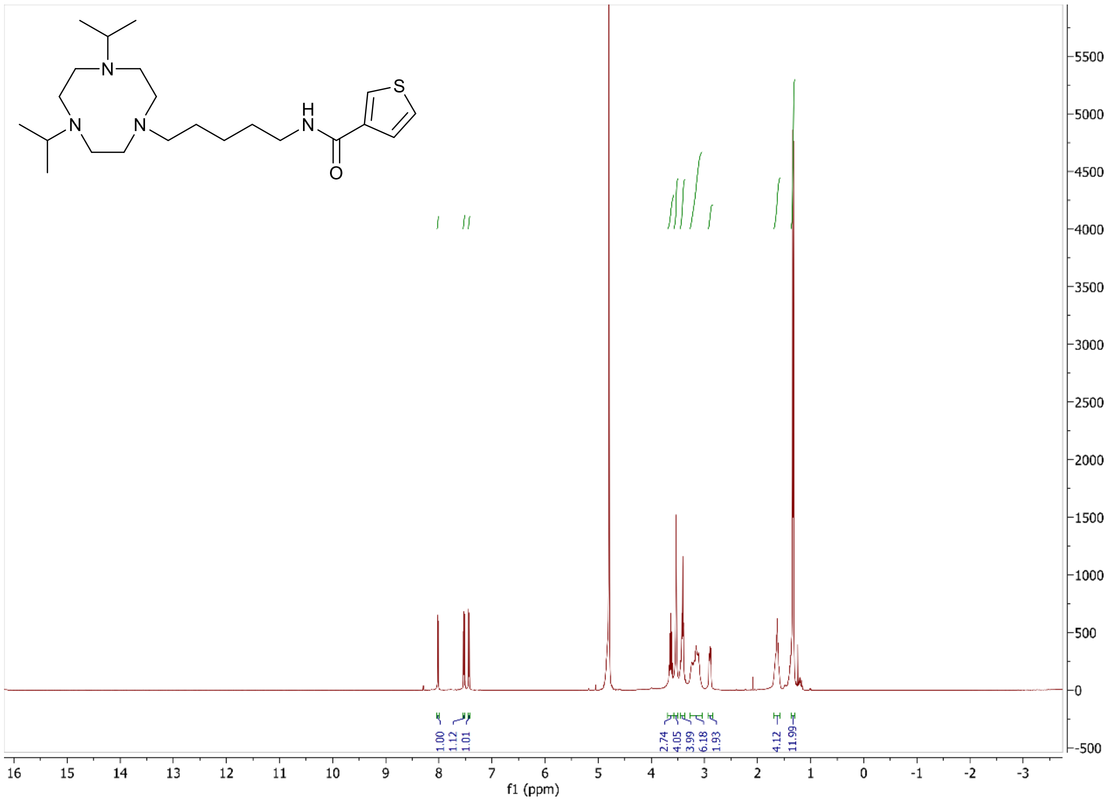

^{1}H (D_2O)

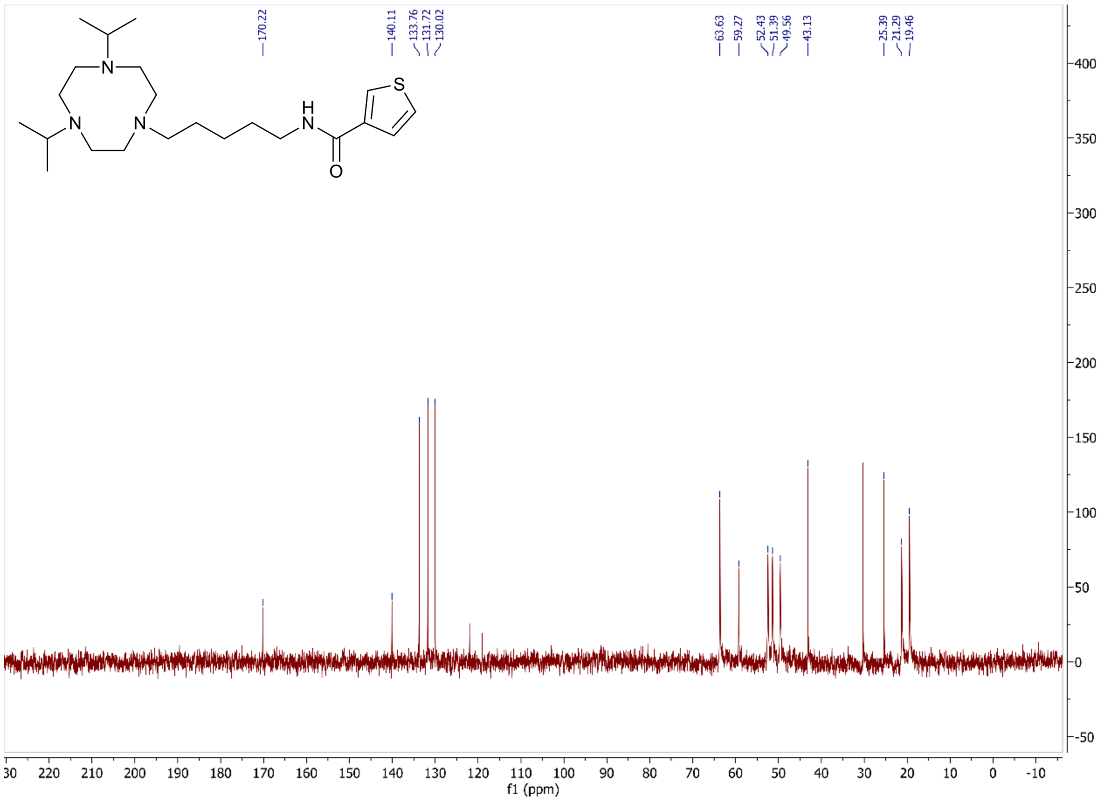

$^{13}C\{^{1}H\}$ (D_2O)

L13a

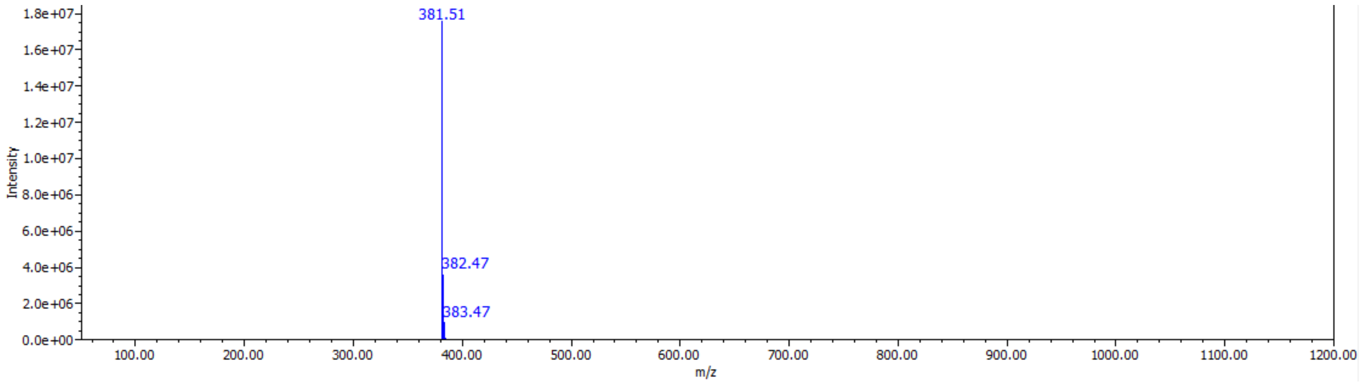

MS-ESI

L13b

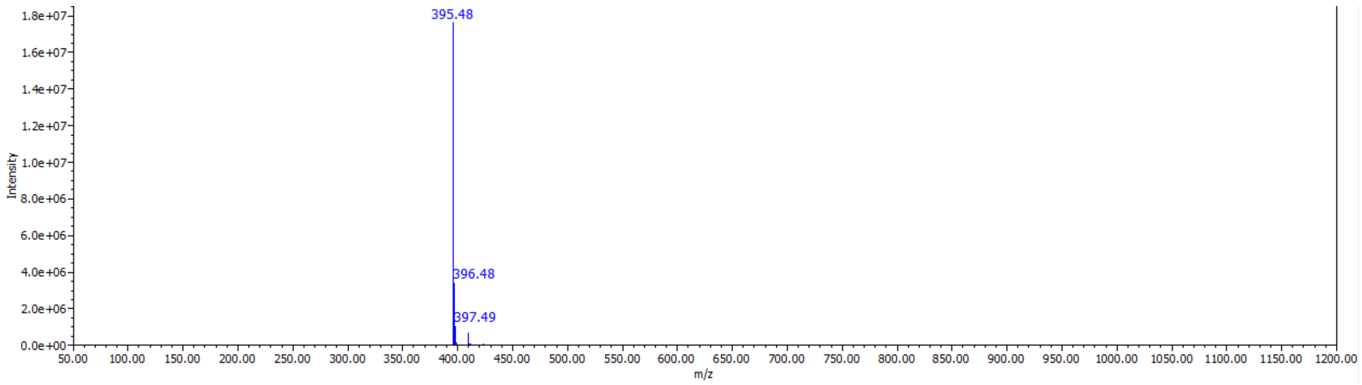

MS-ESI

L13c

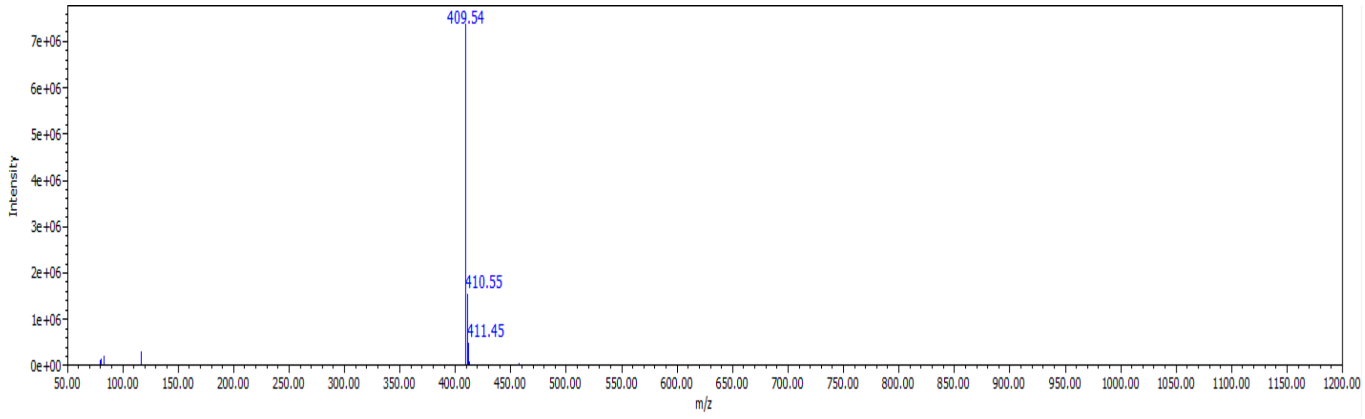

MS-ESI

9a

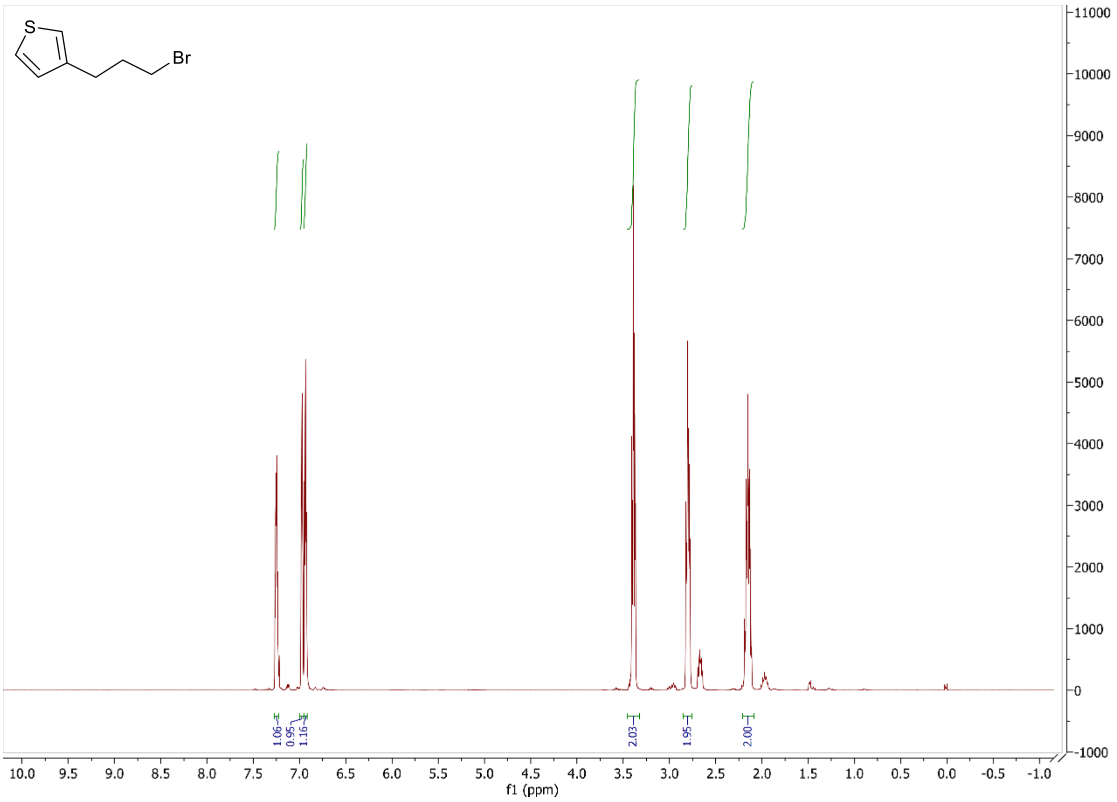

^{1}H (CDCl$_3$)

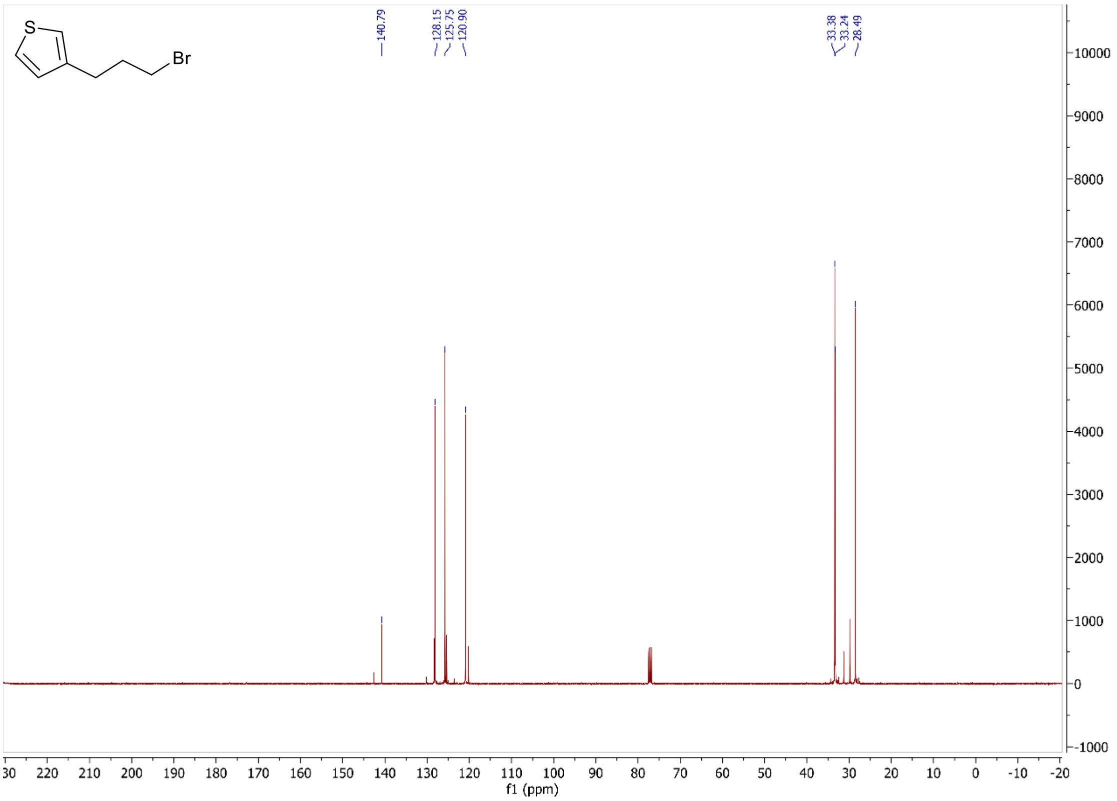

$^{13}C\{^{1}H\}$ (CDCl$_3$)

9c

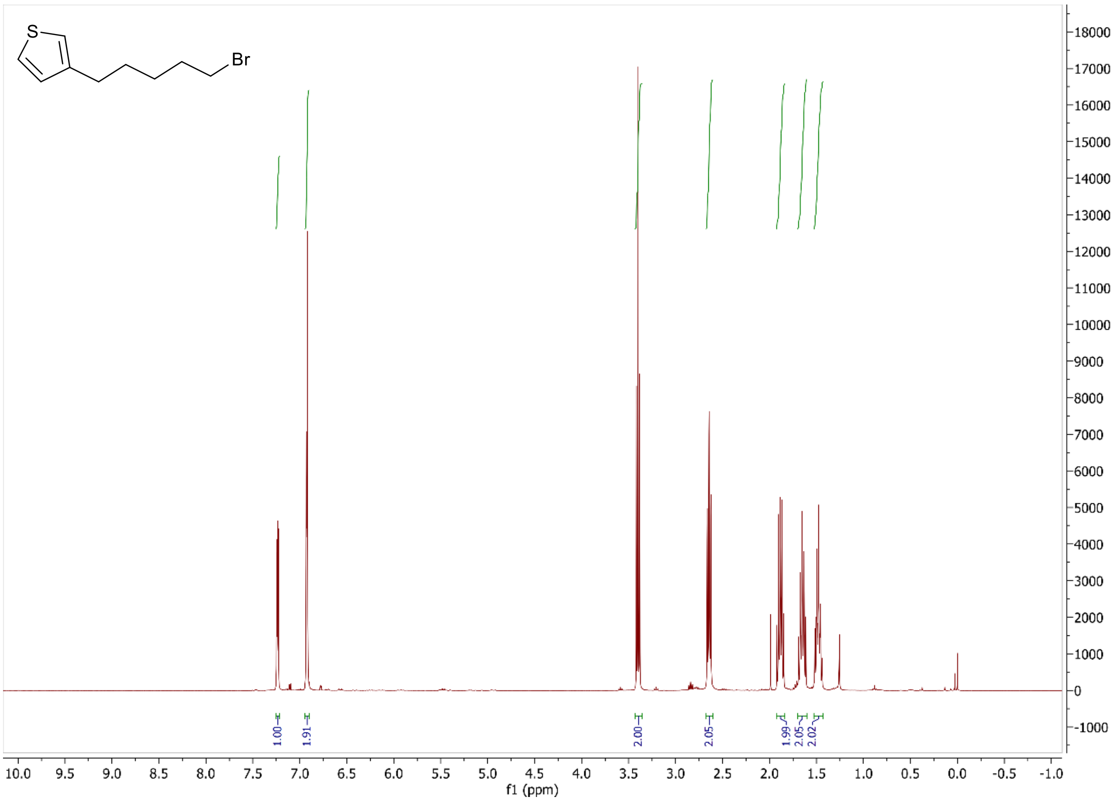

^{1}H ($CDCl_3$)

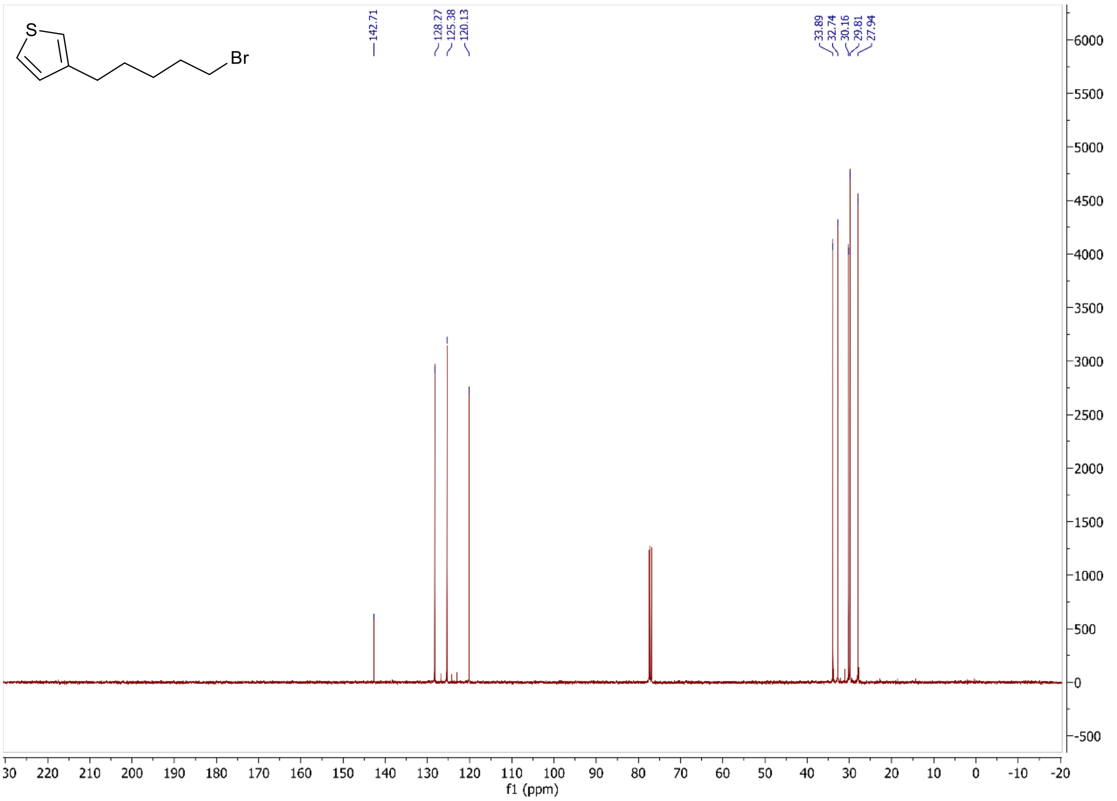

$^{13}C\{^{1}H\}$ ($CDCl_3$)

10a

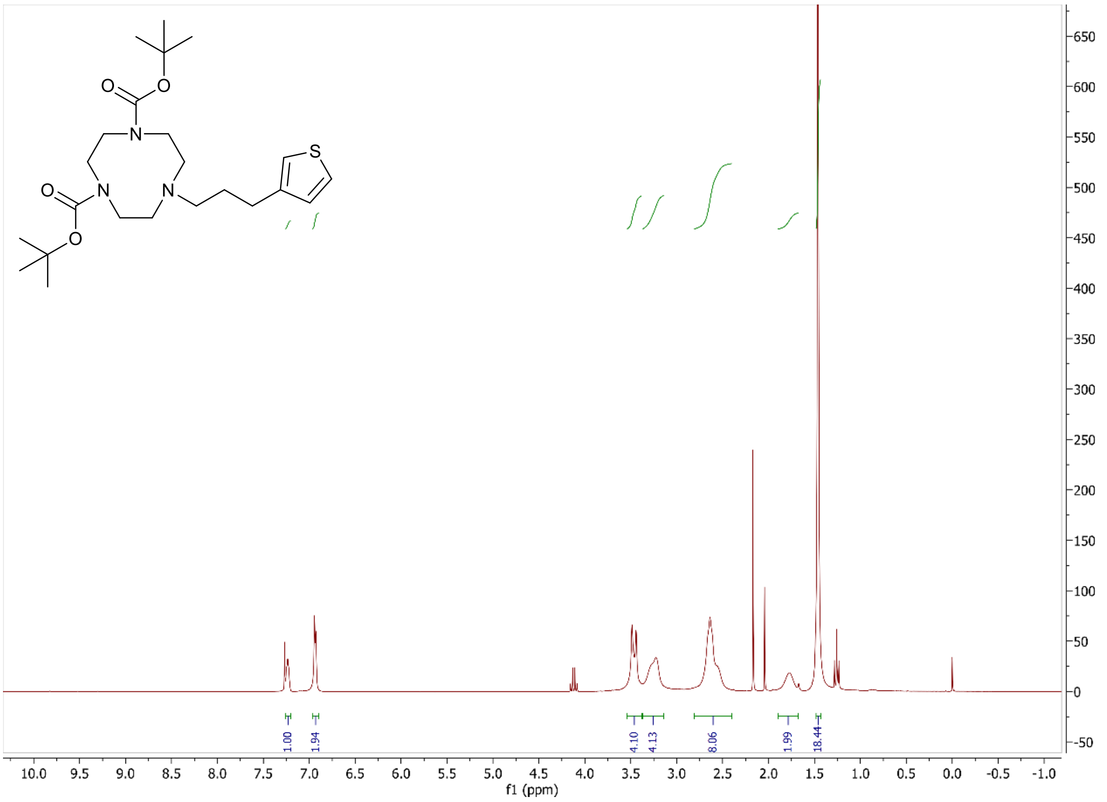

[1]H ($CDCl_3$)

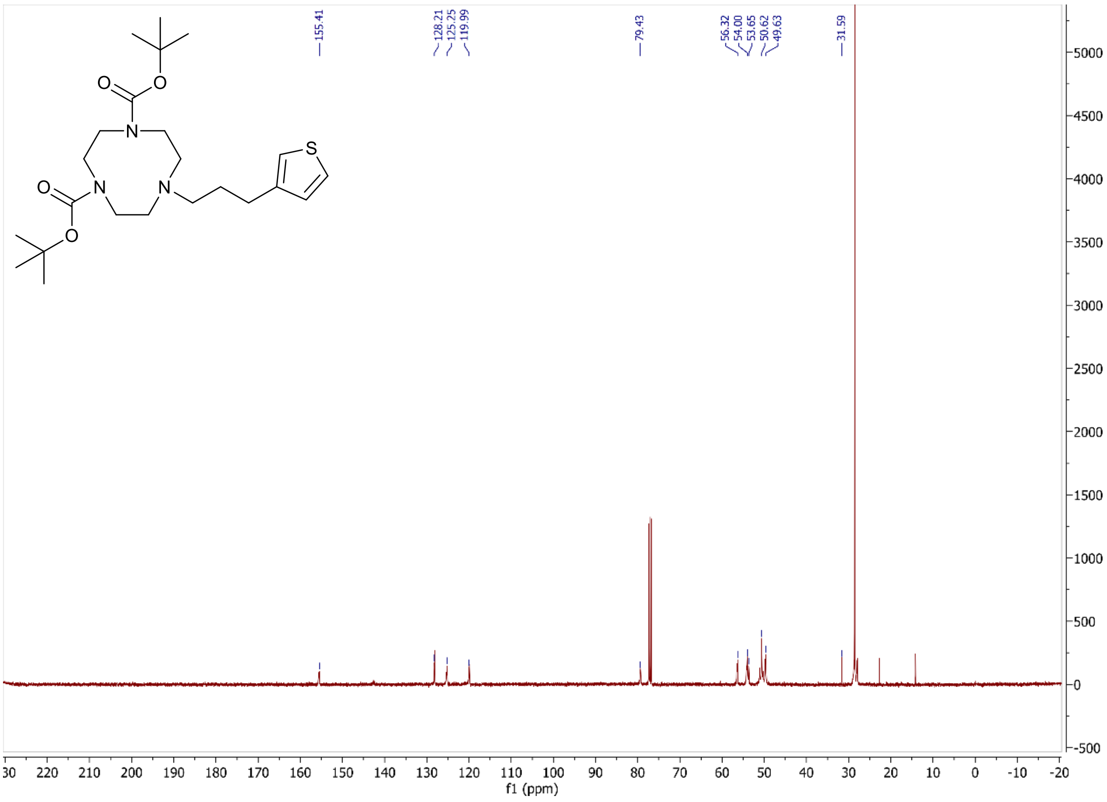

[13]C{[1]H} ($CDCl_3$)

10b

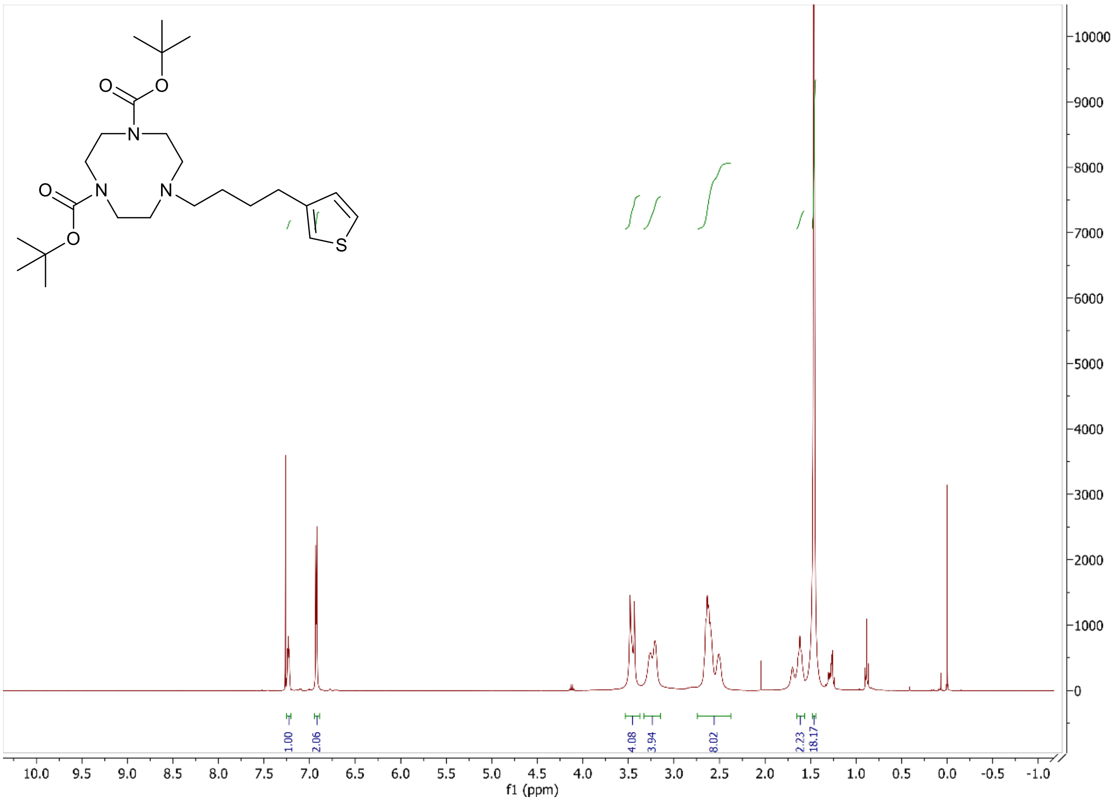

^{1}H ($CDCl_3$)

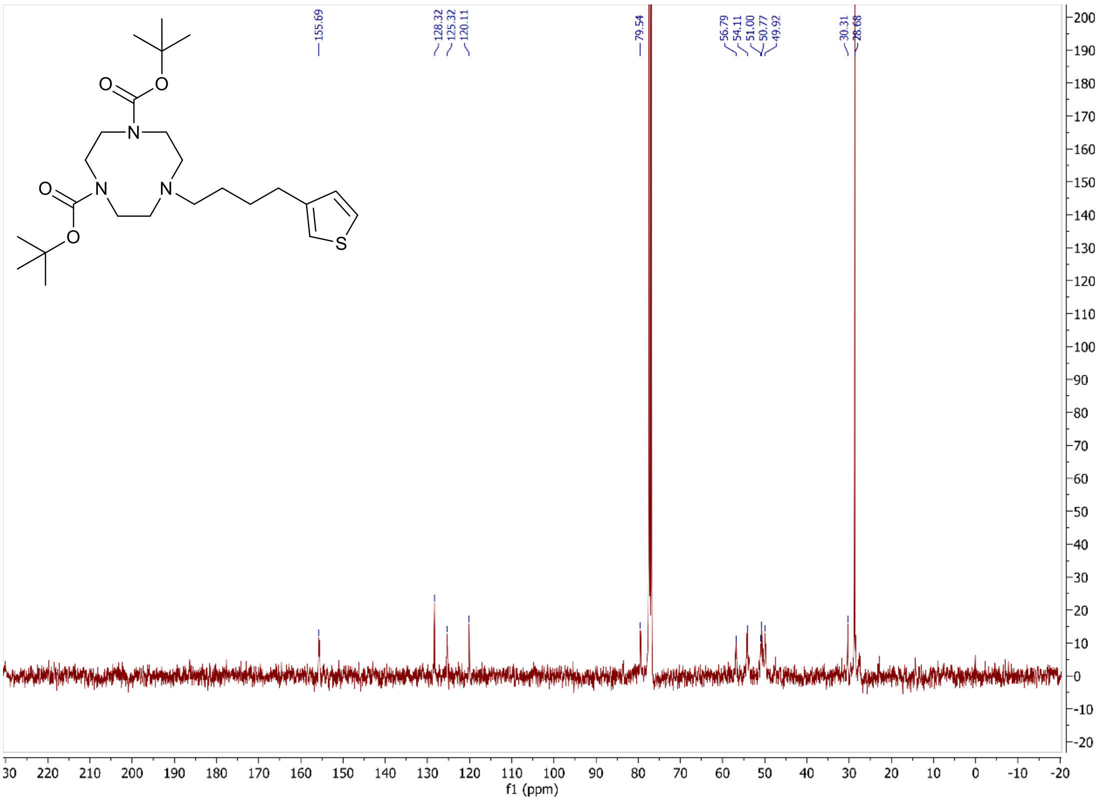

$^{13}C\{^{1}H\}$ ($CDCl_3$)

10c

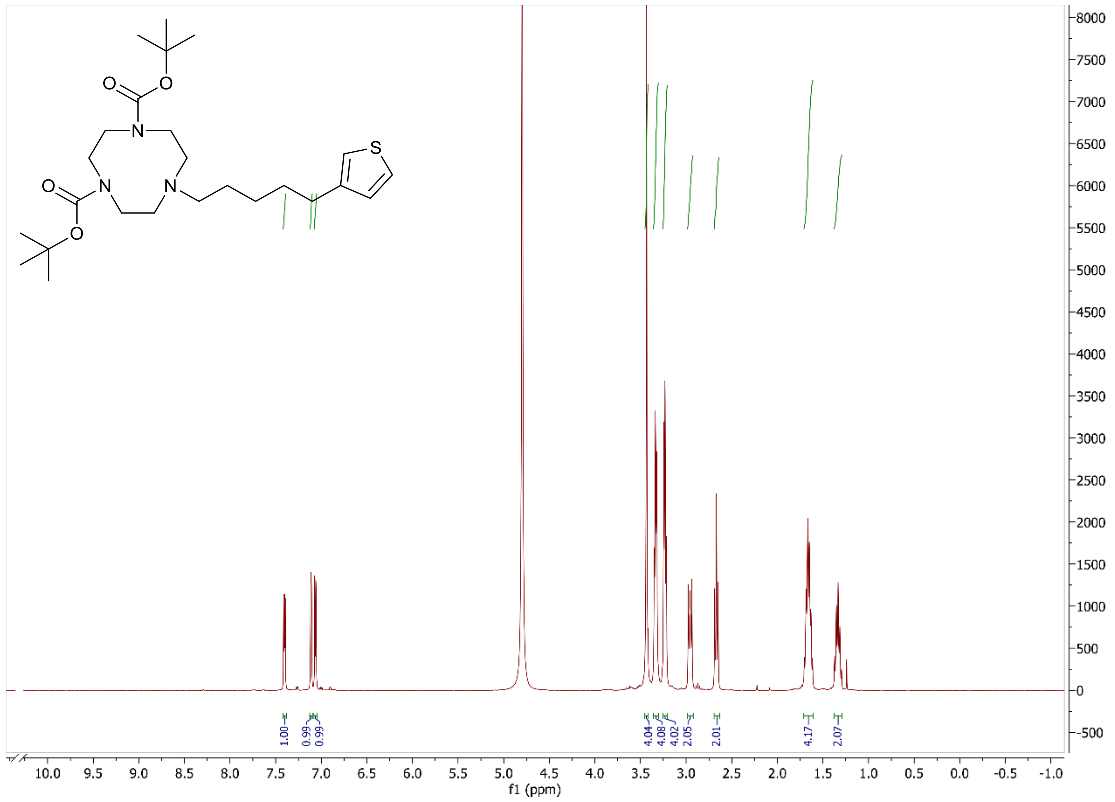

^{1}H ($CDCl_3$)

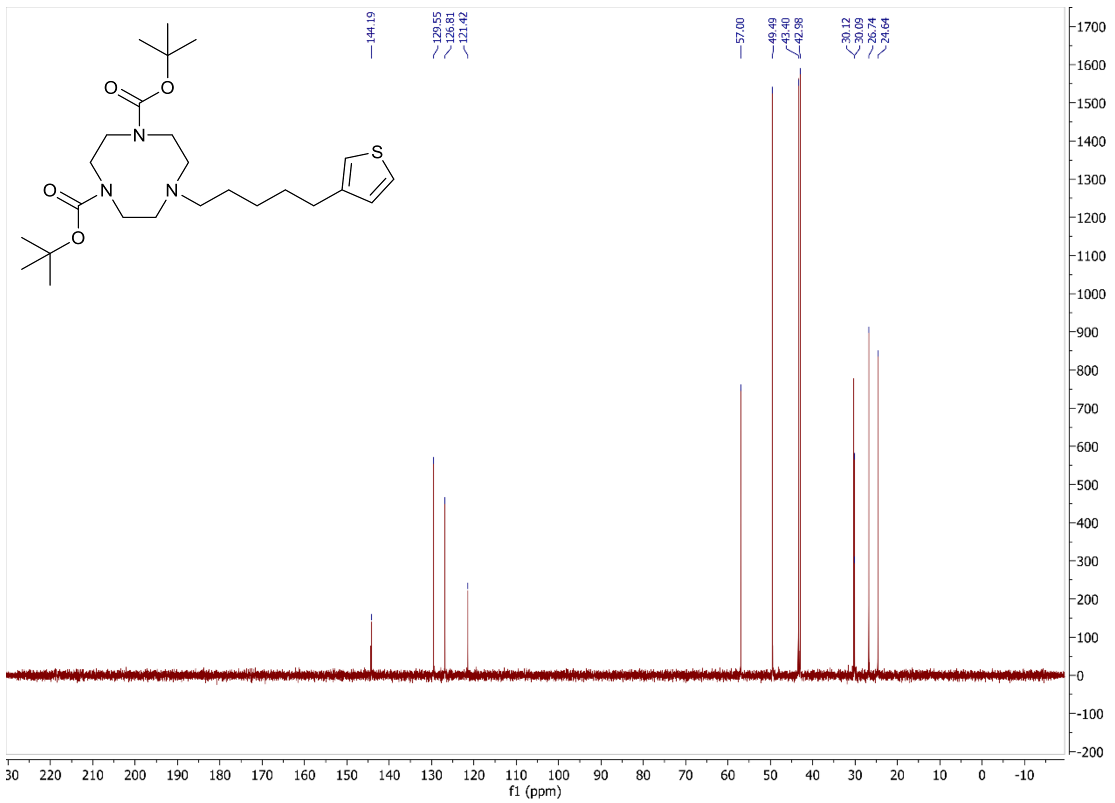

$^{13}C\{^{1}H\}$ ($CDCl_3$)

10a

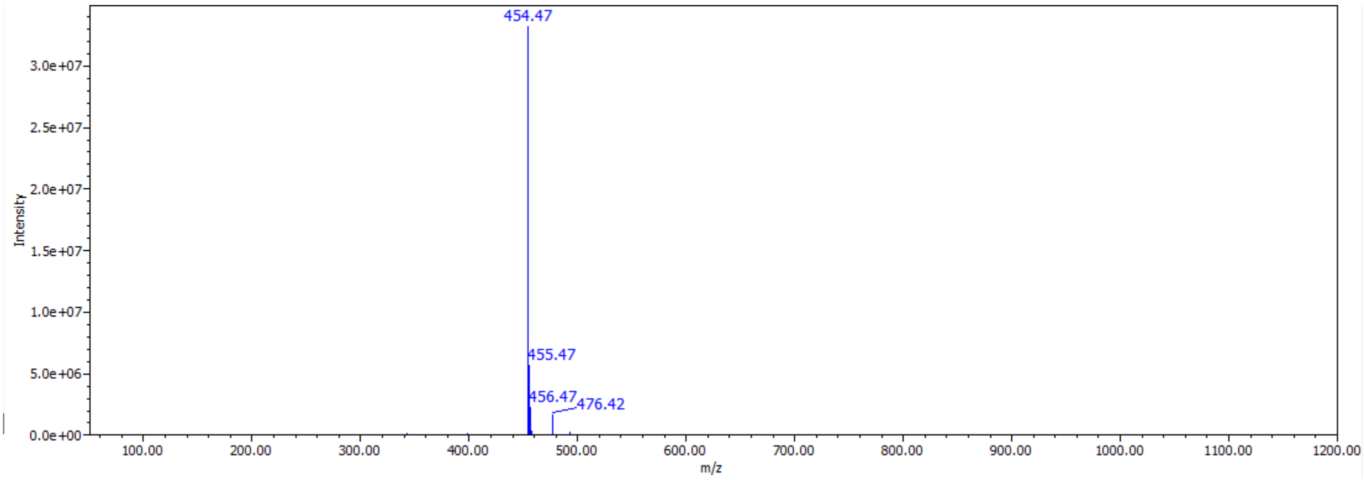

MS-ESI

10b

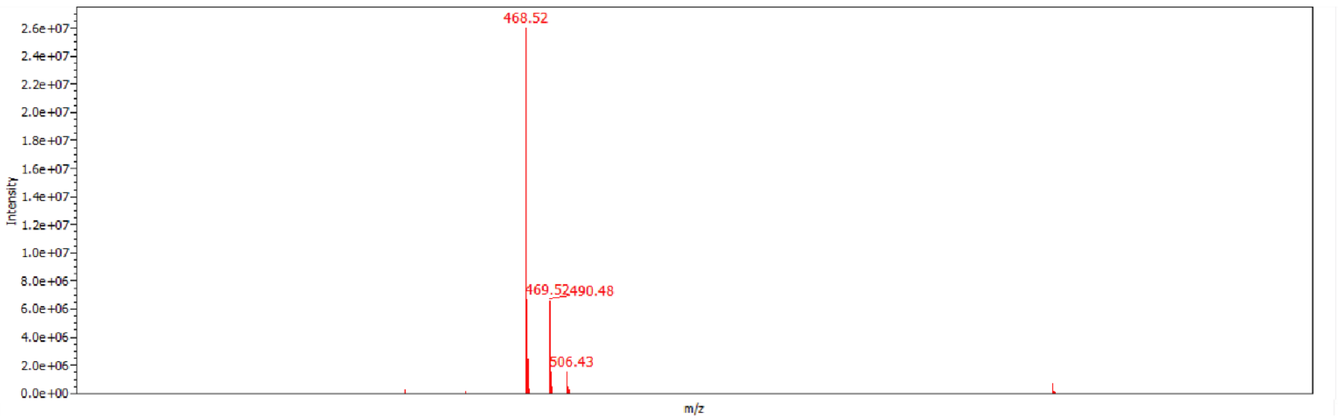

MS-ESI

10c

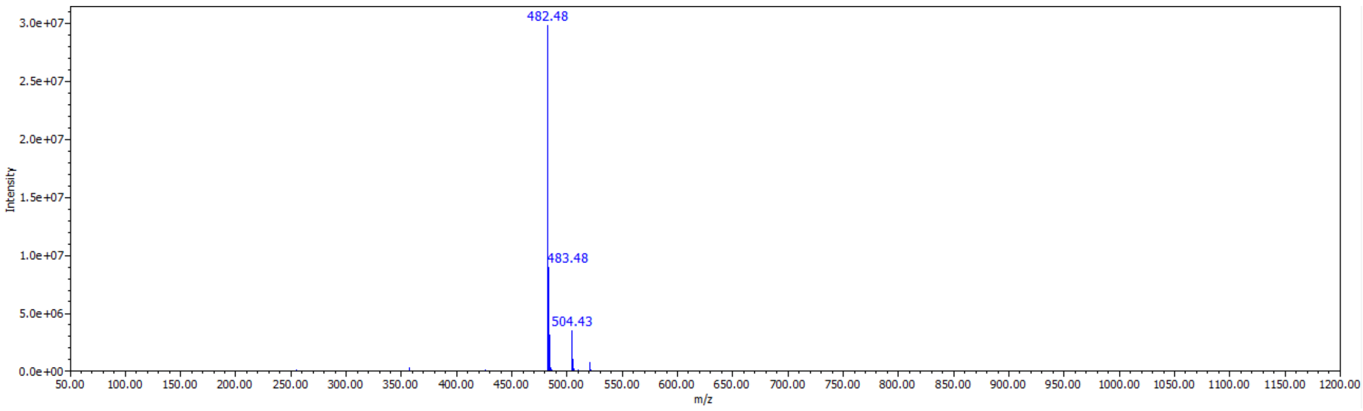

MS-ESI

11a

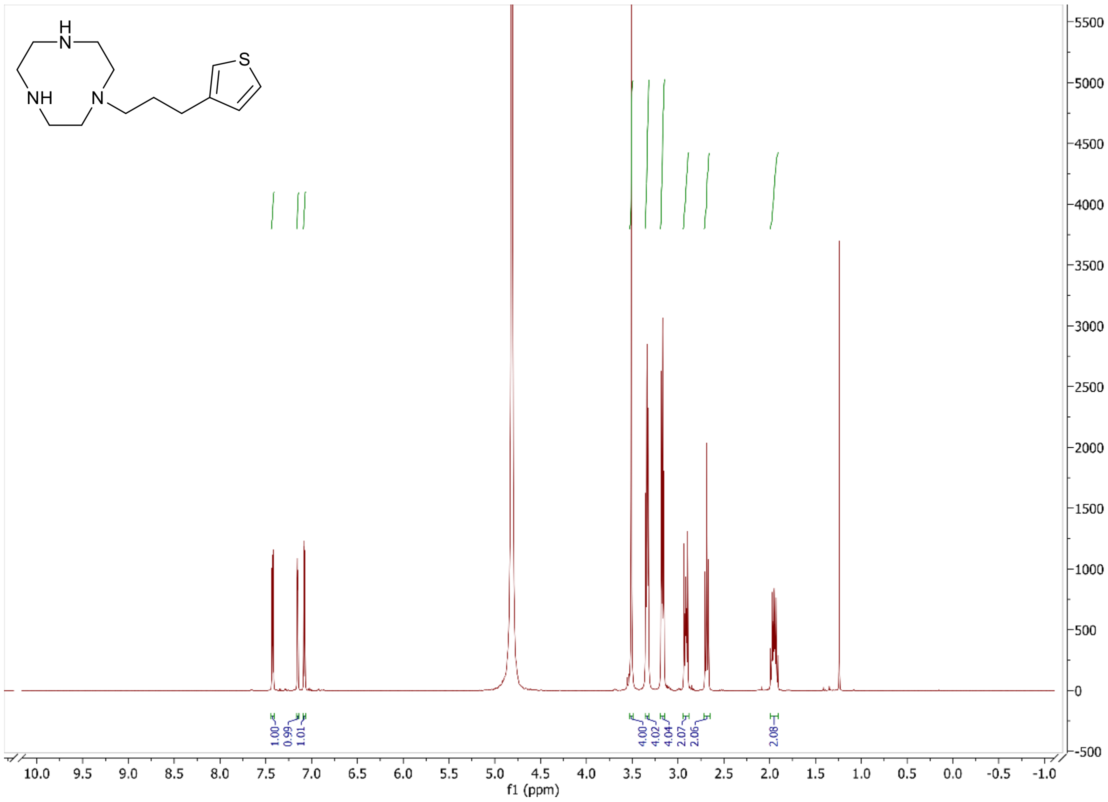

^{1}H (D_2O)

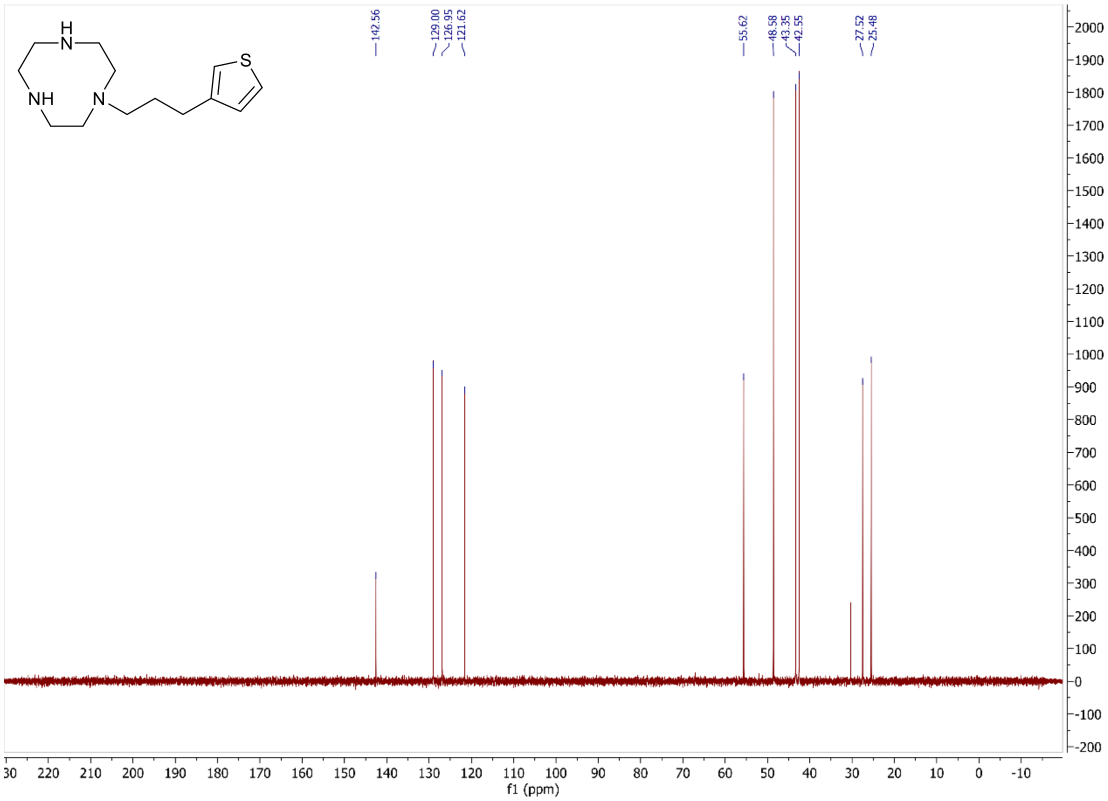

$^{13}C\{^{1}H\}$ (D_2O)

11b

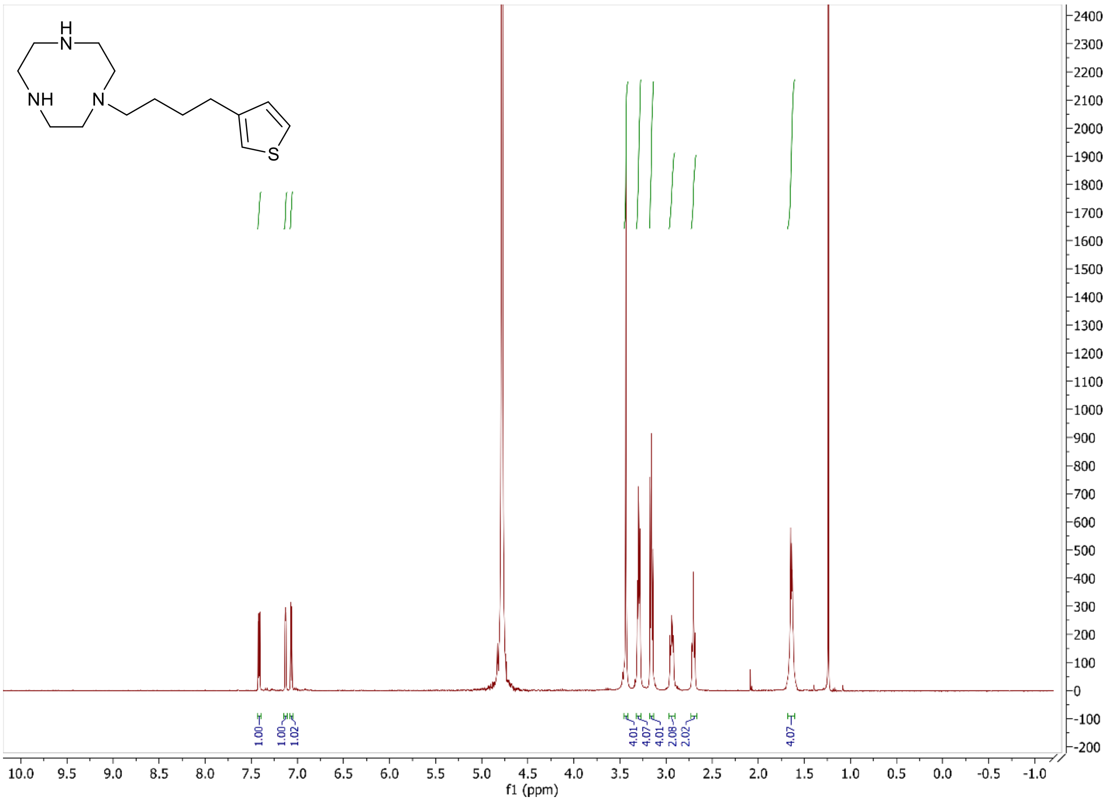

^{1}H (D_2O)

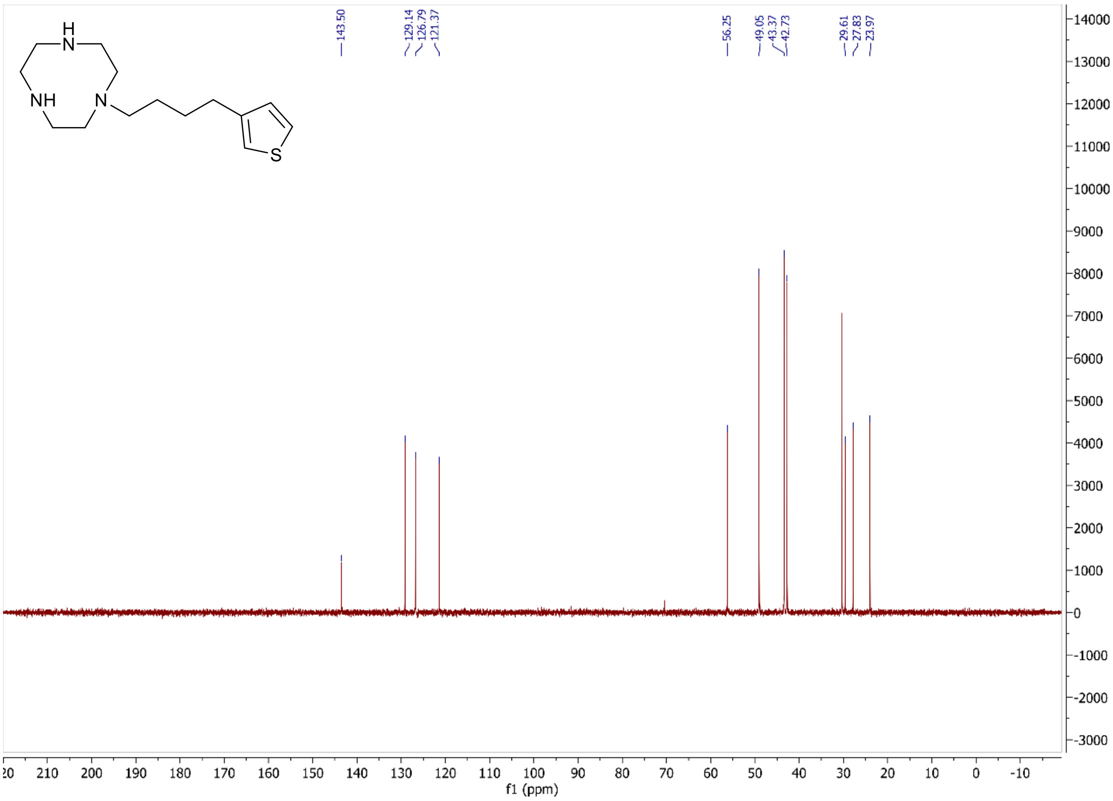

$^{13}C\{^{1}H\}$ (D_2O)

11c

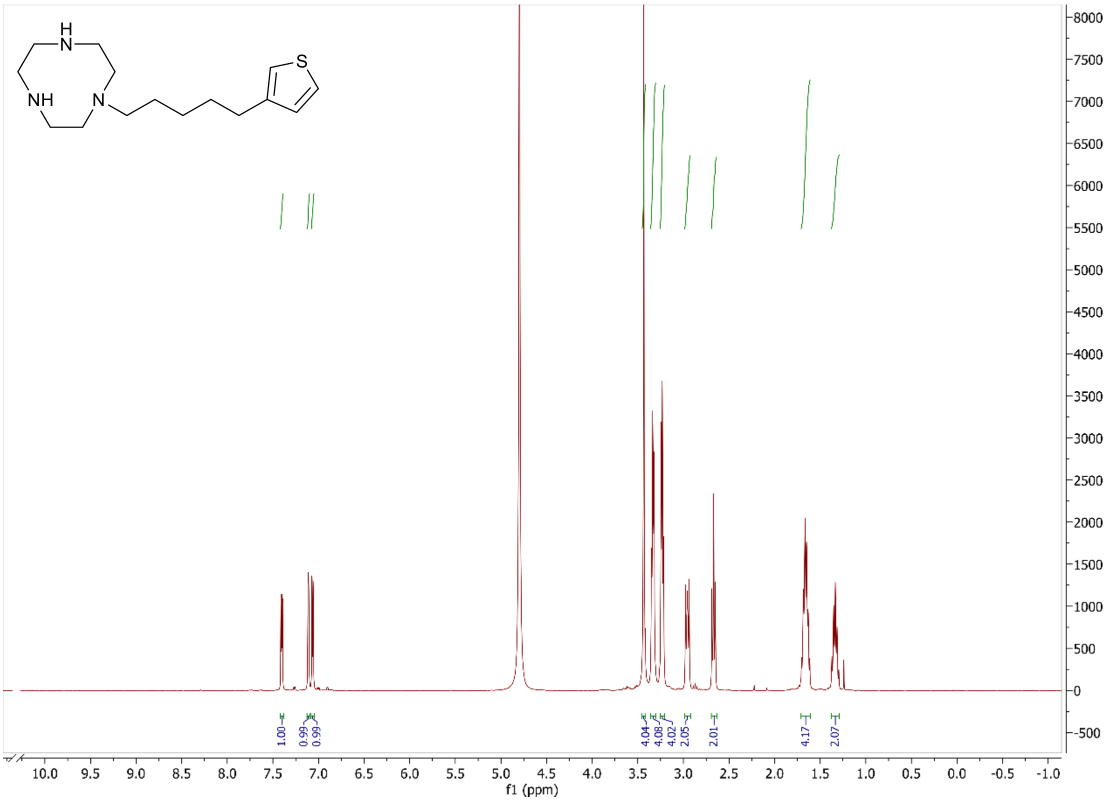

^{1}H (D_2O)

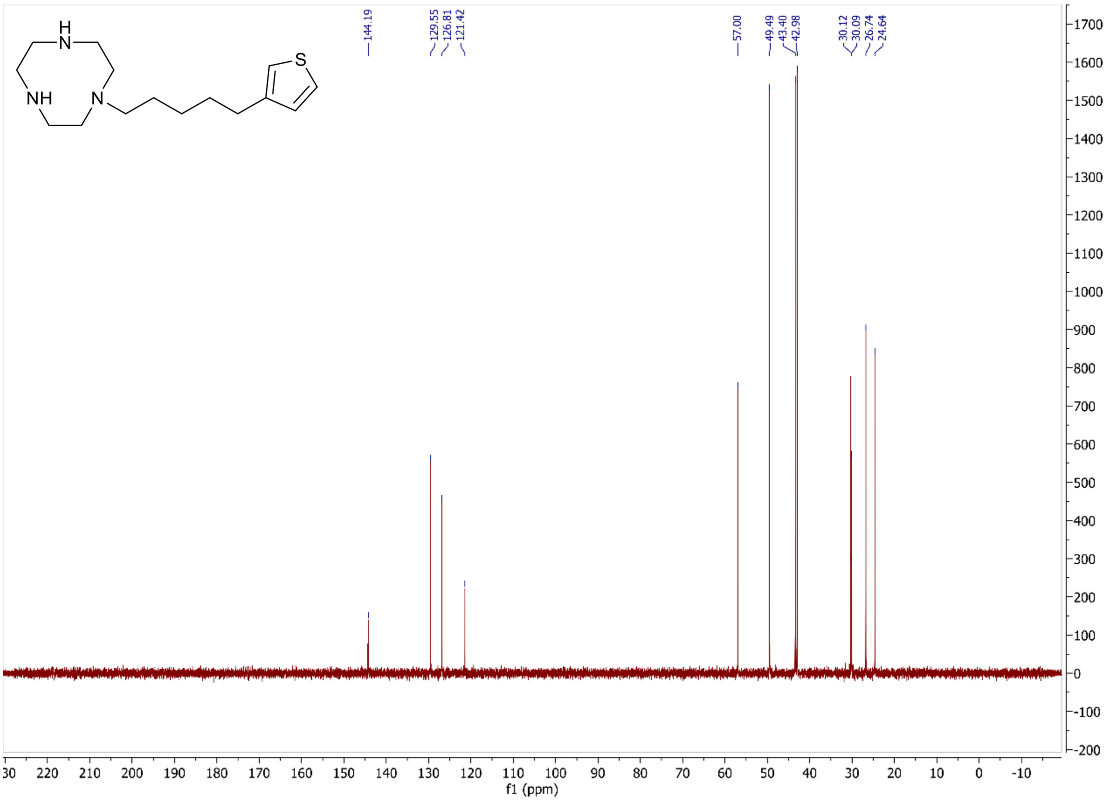

$^{13}C\{^{1}H\}$ (D_2O)

11a

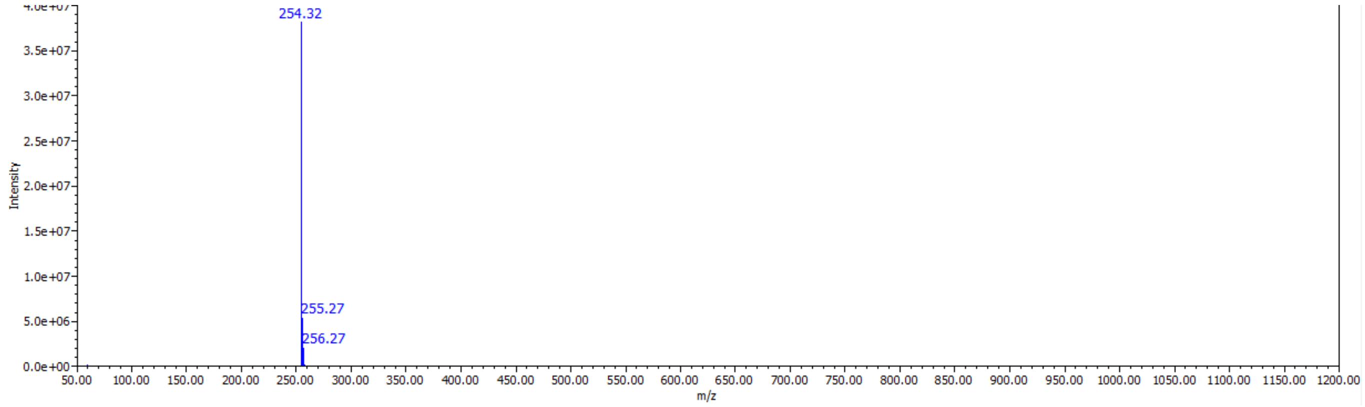

MS-ESI

11b

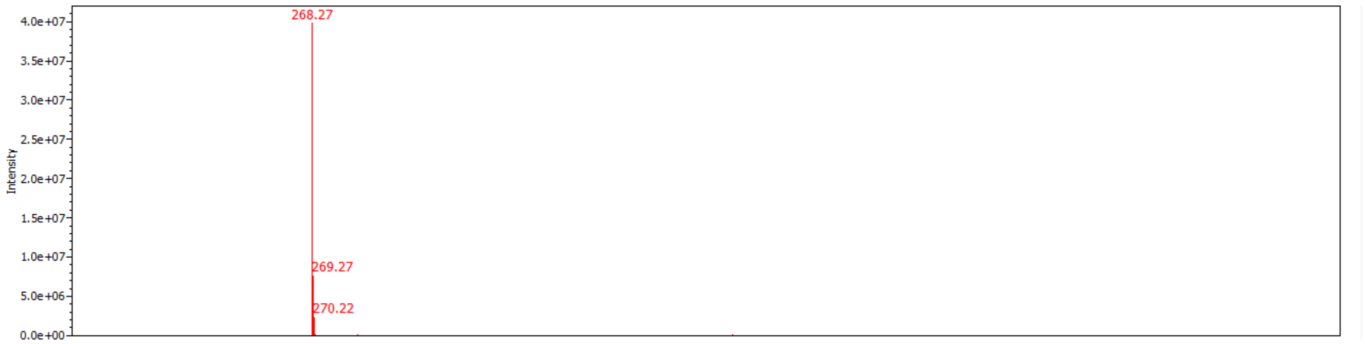

MS-ESI

11c

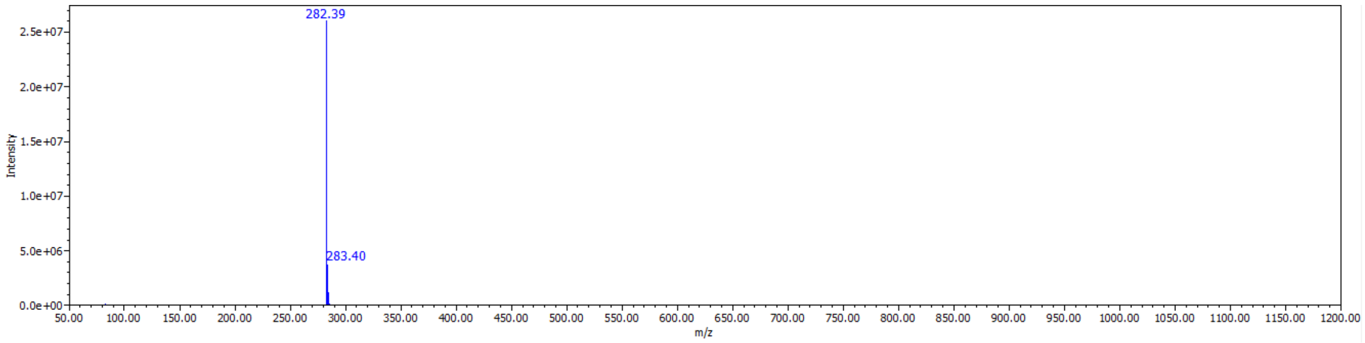

MS-ESI

L14a

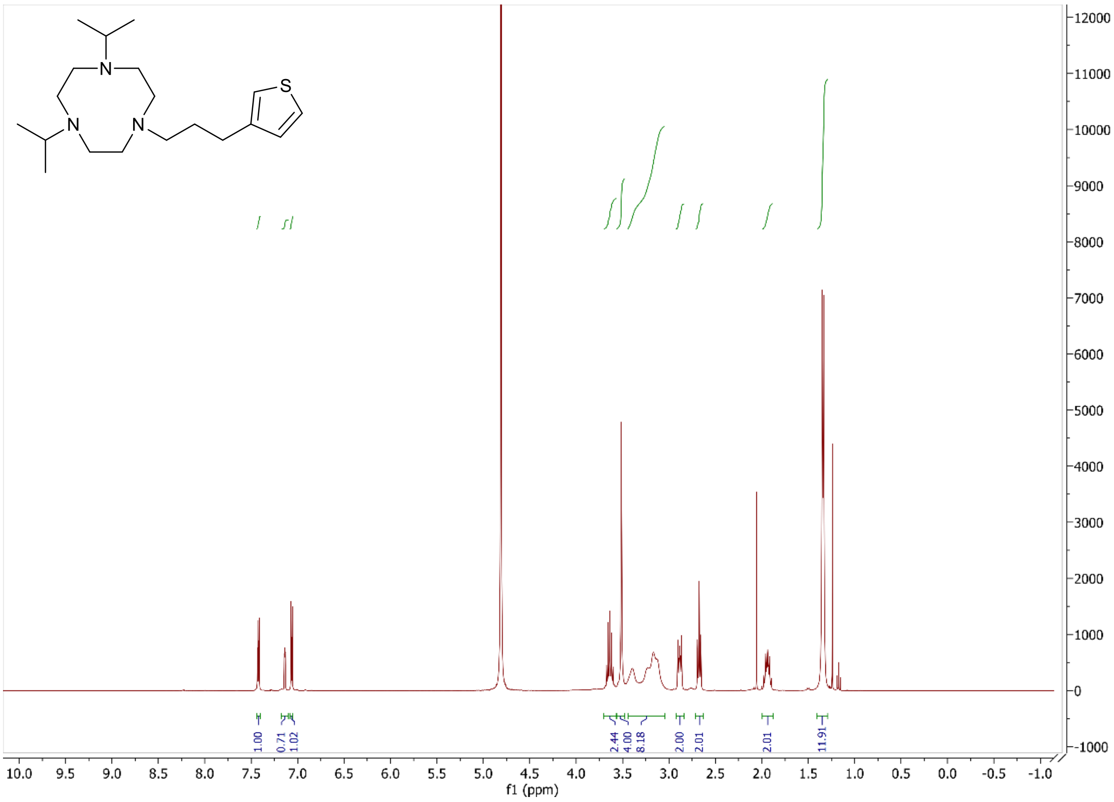

^{1}H (D_2O)

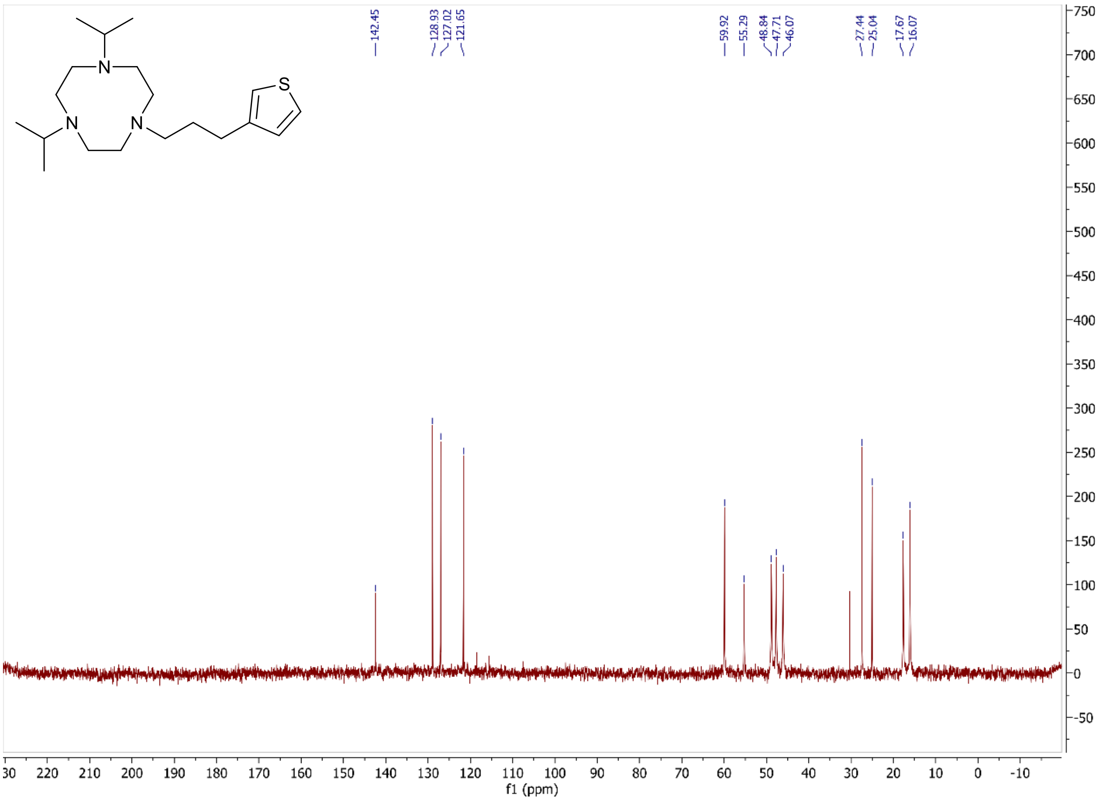

$^{13}C\{^{1}H\}$ (D_2O)

L14b

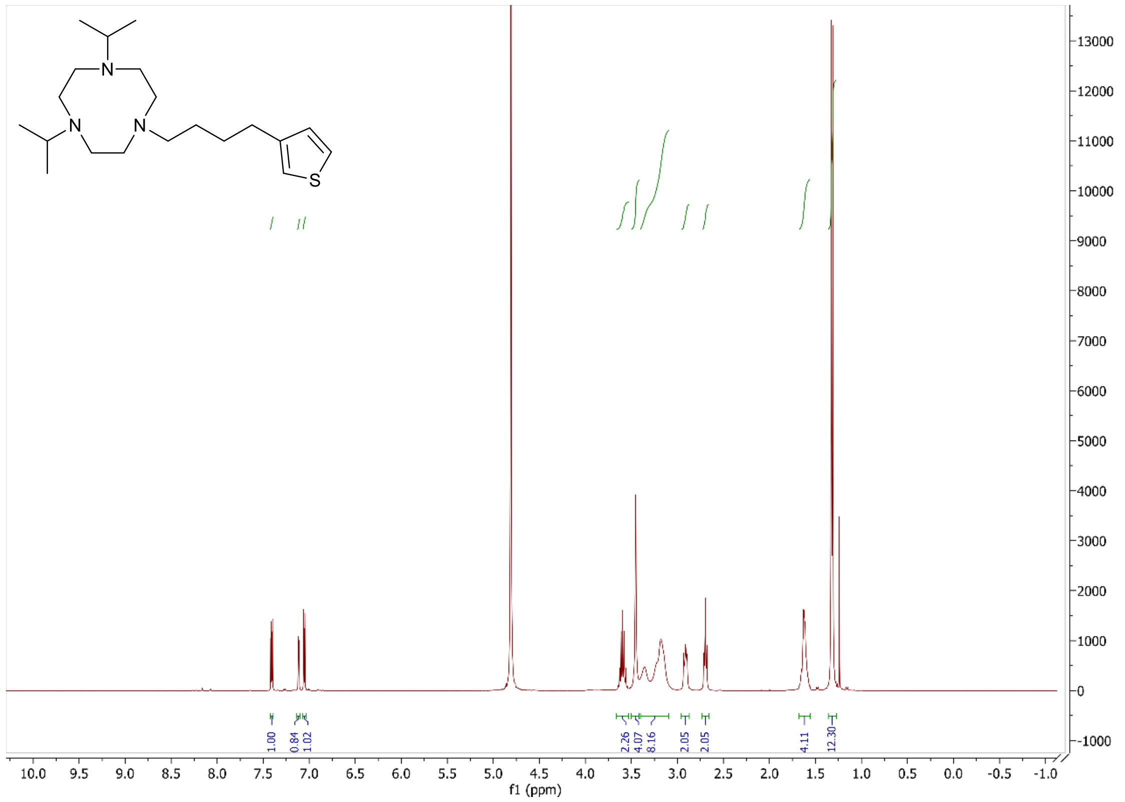

^1H (D_2O)

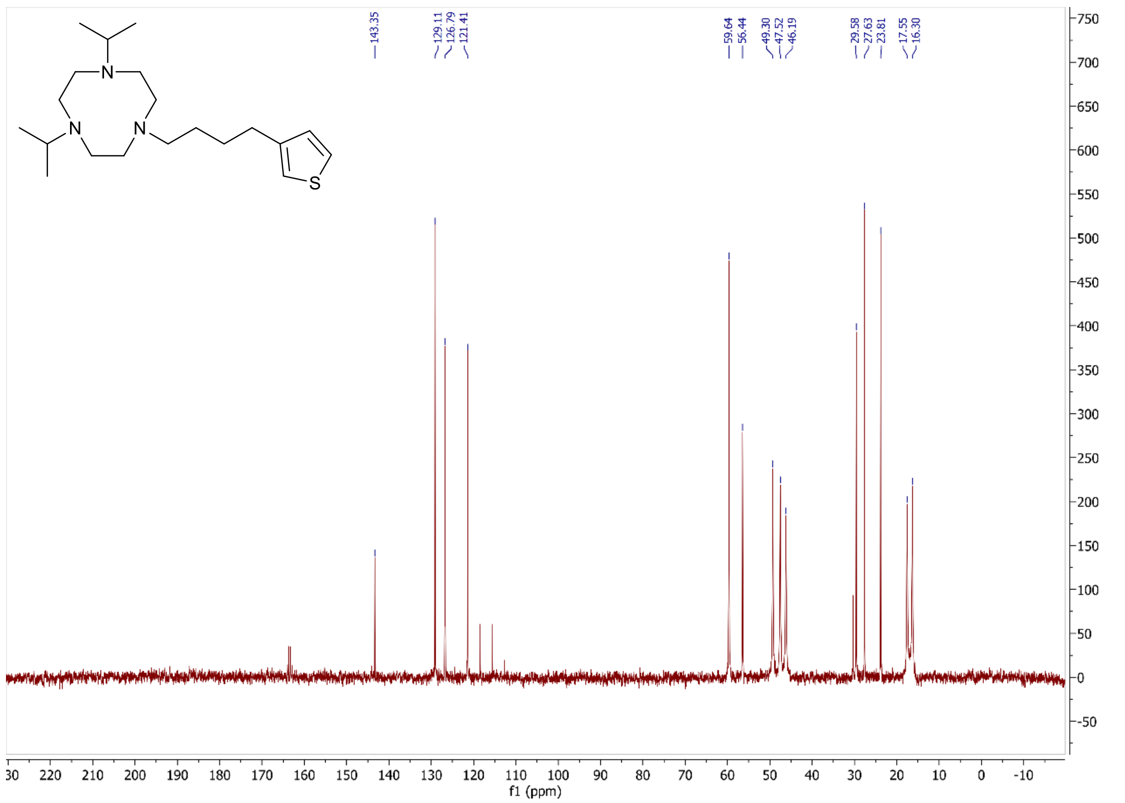

$^{13}C\{^1H\}$ (D_2O)

L14c

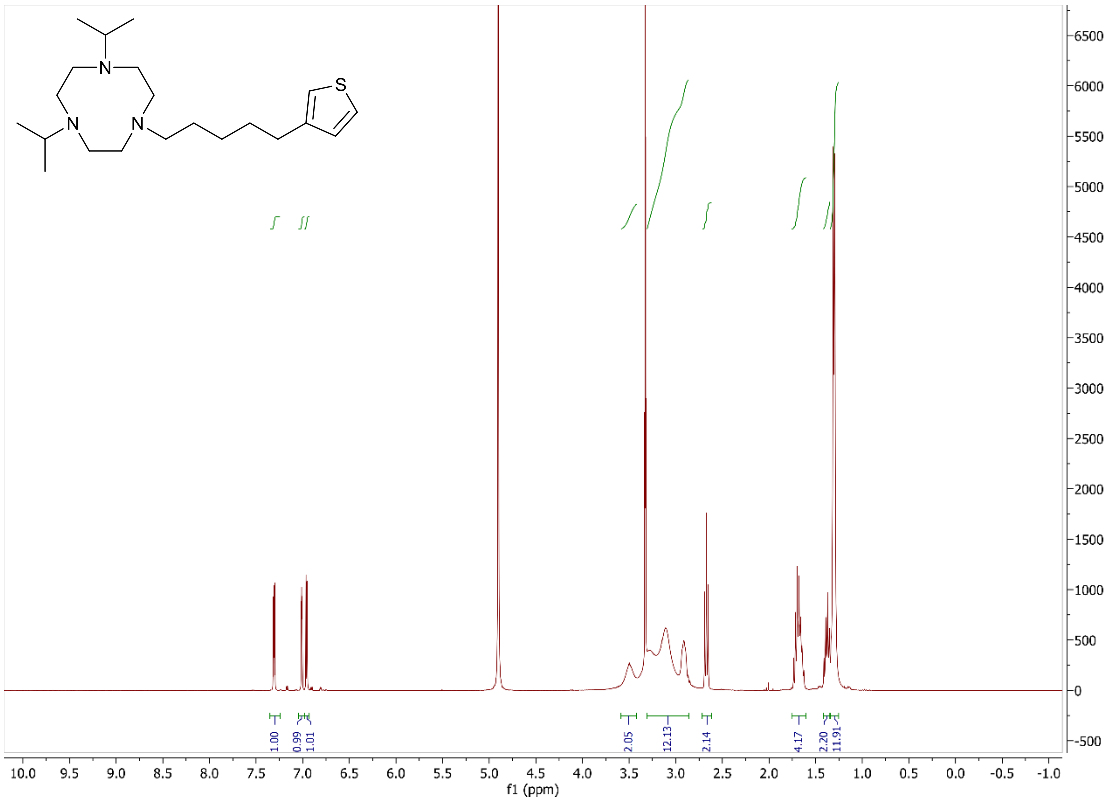

^{1}H (CD_3OD)

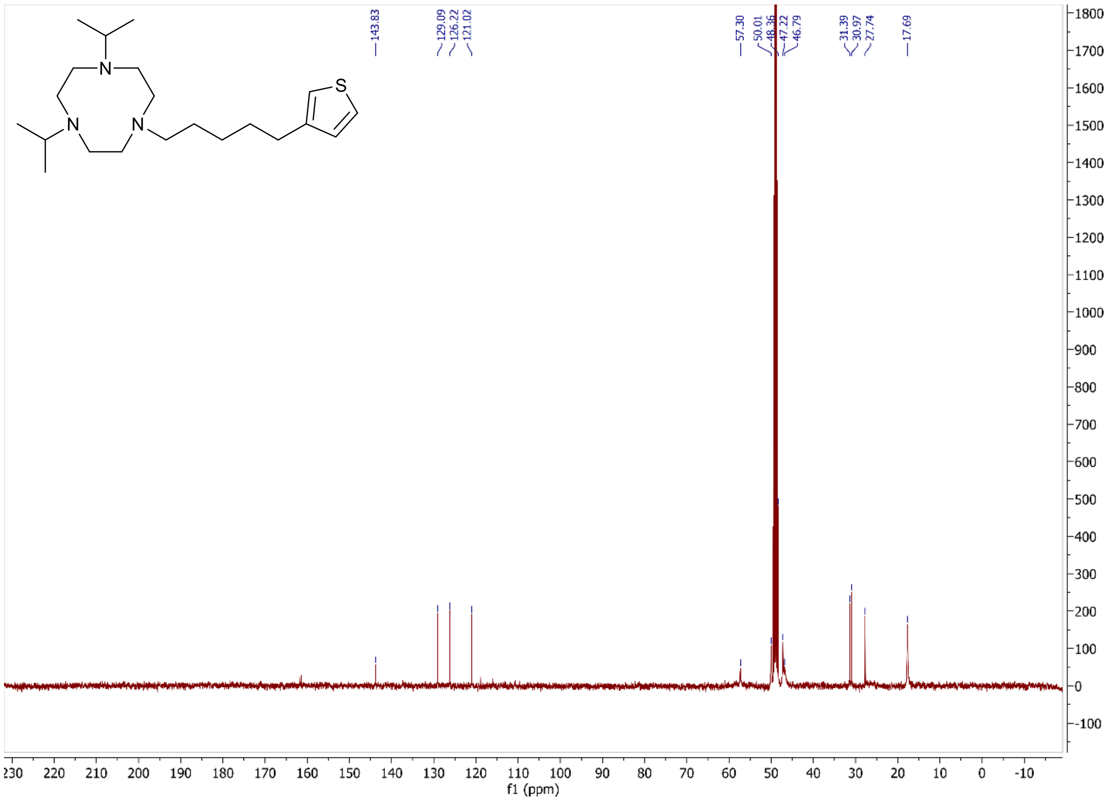

$^{13}C\{^{1}H\}$ (CD_3OD)

L14a

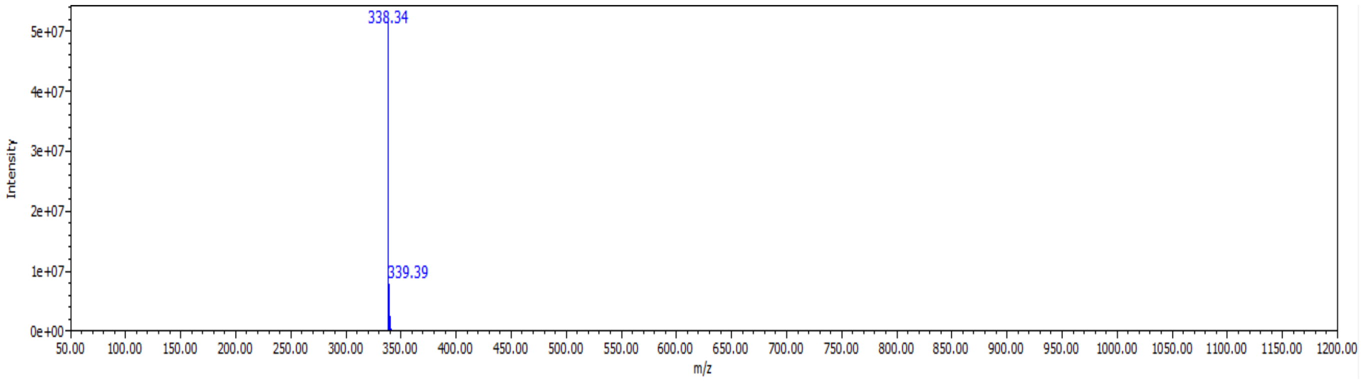

MS-ESI

L14b

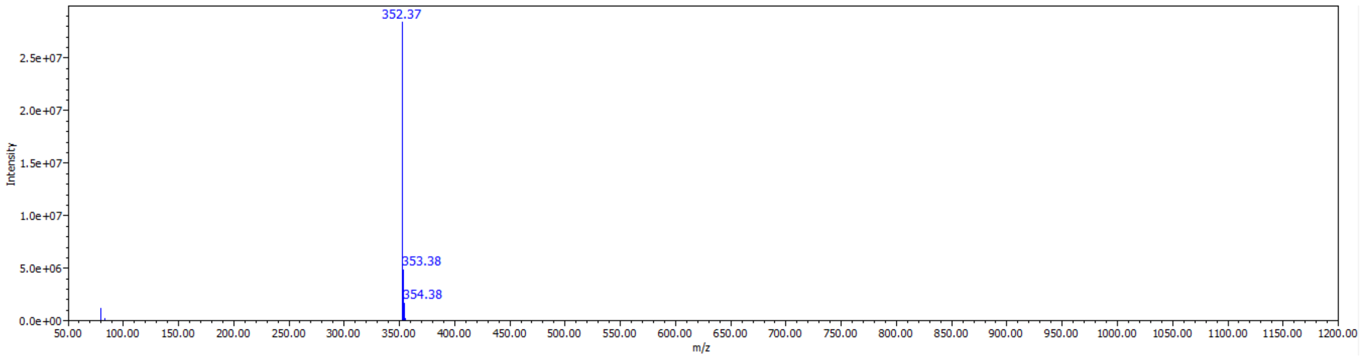

MS-ESI

L14c

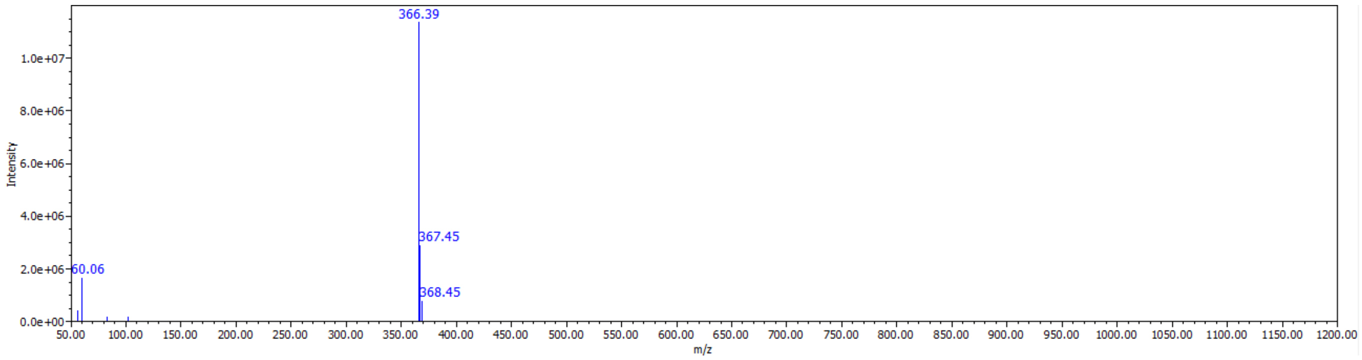

MS-ESI

HPLC chromatograms of ligands L13a–c

There are several peaks in the UV-Vis spectrum which have the same mass spectra. This is probably caused by separation of different conformers (or ionic pairs with trifluoroacetic acid) due to freezing of the amide group.

L13a

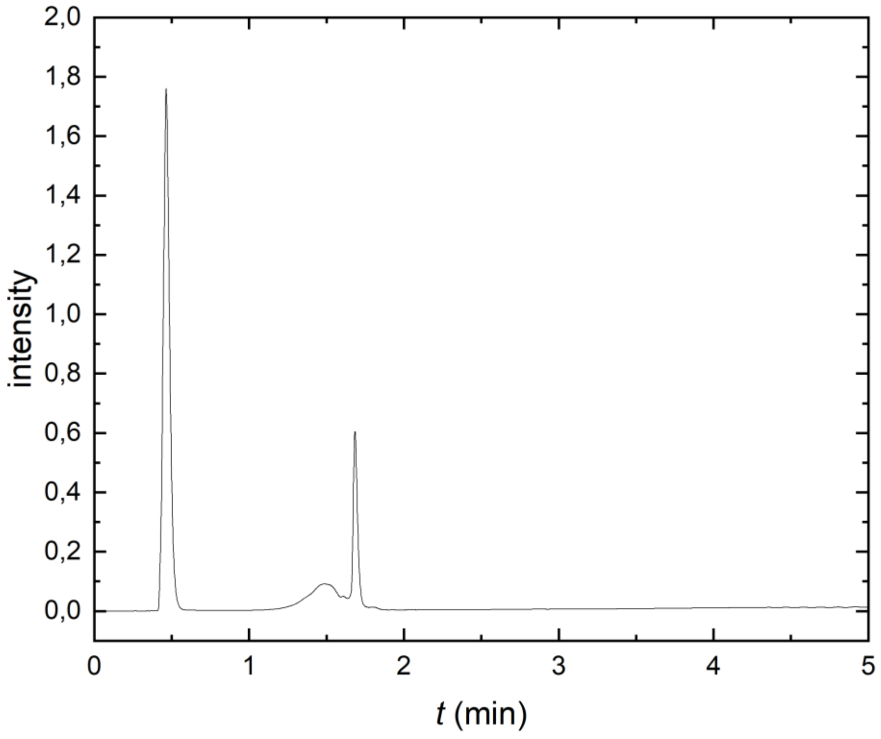

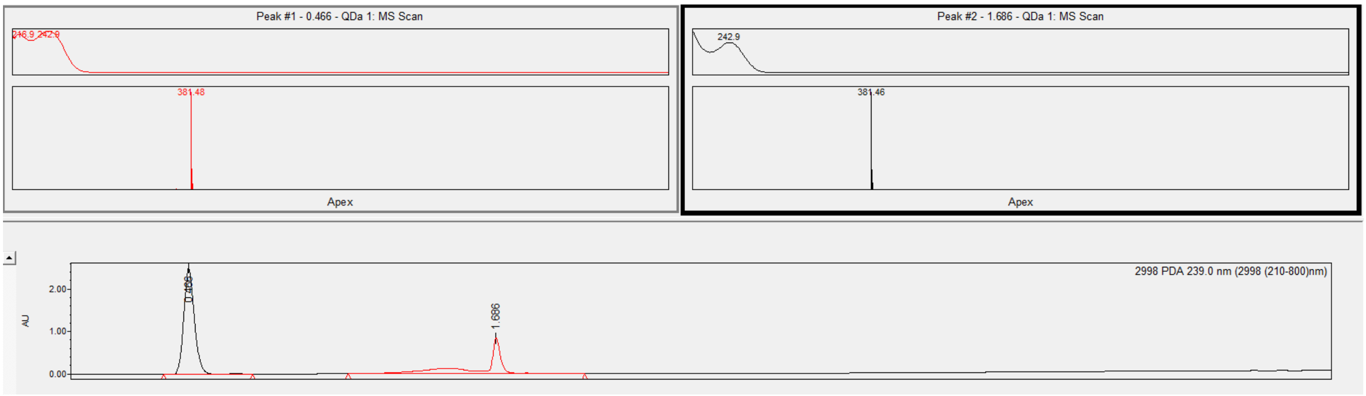

L13b

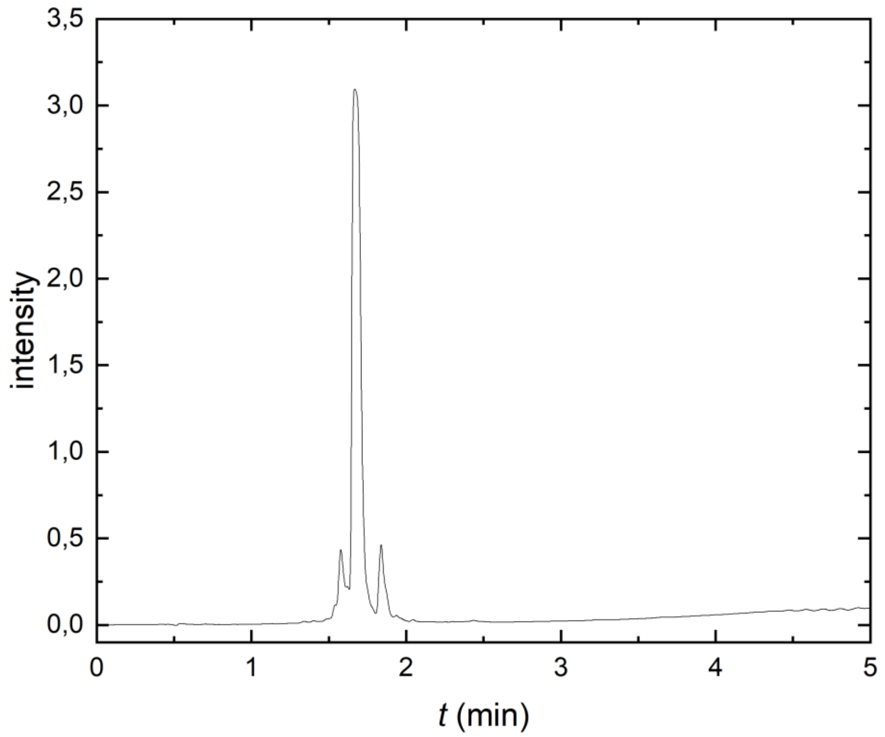

intensity
3,5
3,0
2,5
2,0
1,5
1,0
0,5
0,0
0
1
2
3
4
5
t (min)

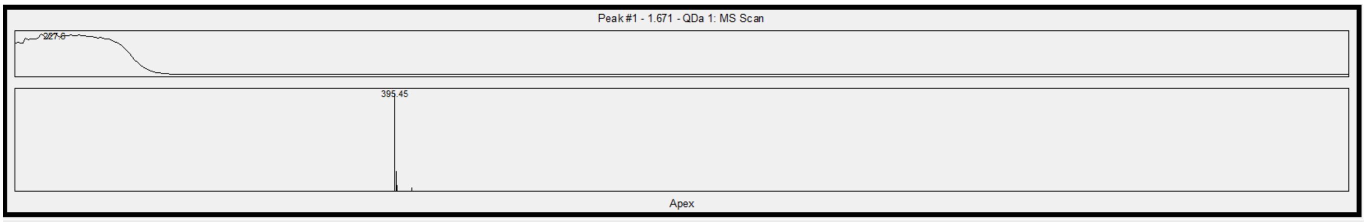

Peak #1 - 1.671 - QDa 1: MS Scan
395.45
Apex

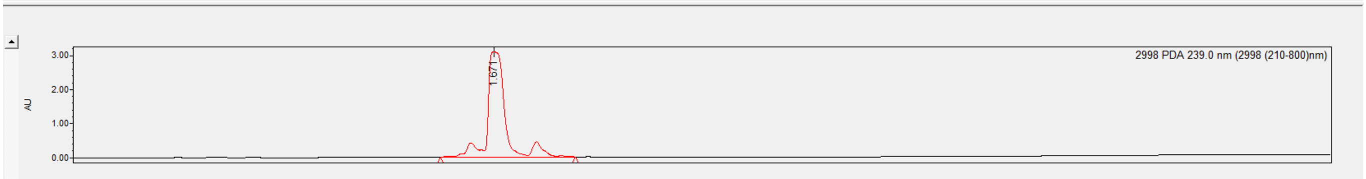

2998 PDA 239.0 nm (2998 (210-800)nm)
AU
3.00
2.00
1.00
0.00
1.671

L13c

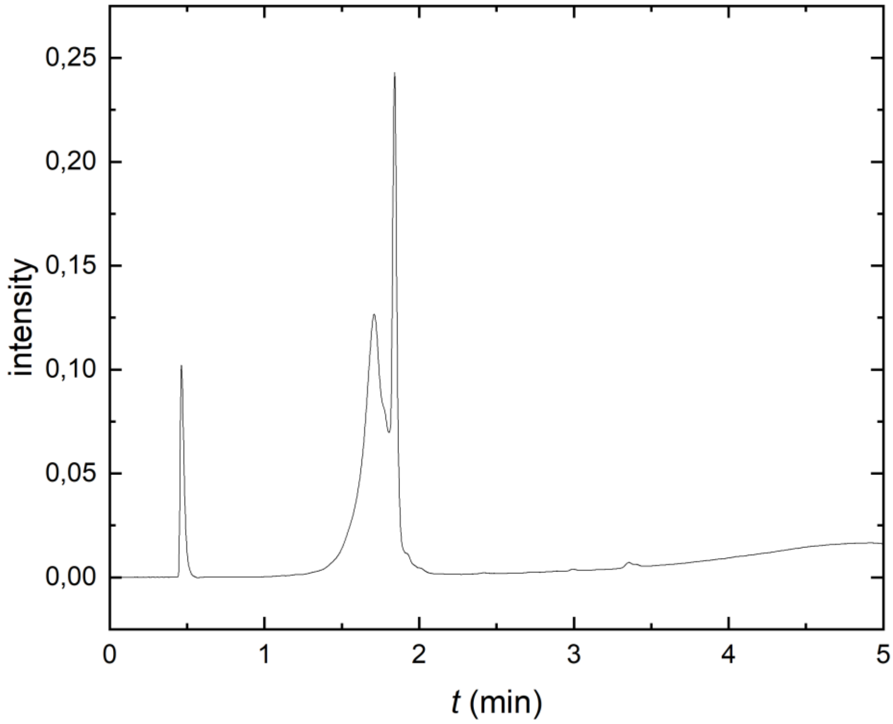

intensity
0,25
0,20
0,15
0,10
0,05
0,00
0
1
2
3
4
5
t (min)

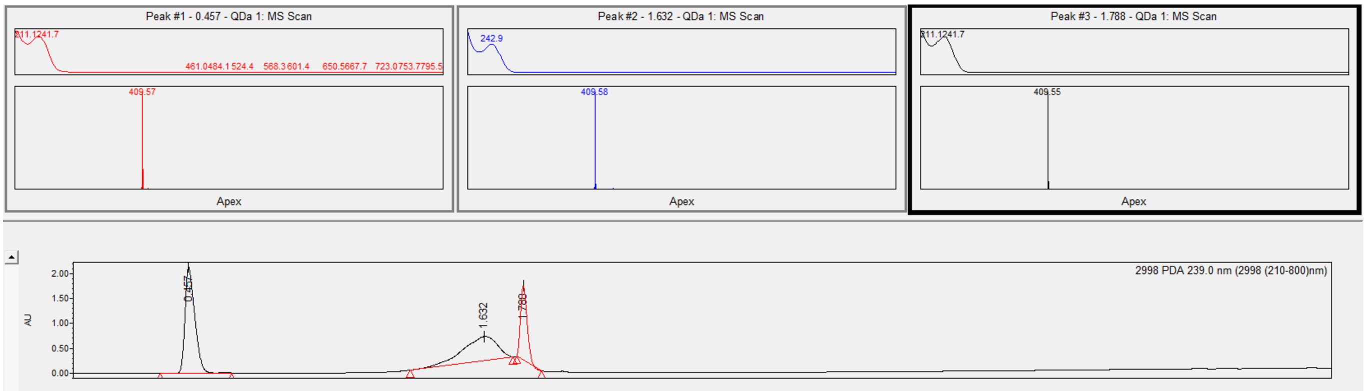

Peak #1 - 0.457 - QDa 1: MS Scan
409.57
Apex
Peak #2 - 1.632 - QDa 1: MS Scan
242.9
409.58
Apex
Peak #3 - 1.788 - QDa 1: MS Scan
409.55
Apex
AU
2.00
1.50
1.00
0.50
0.00
0.457
1.632
1.788
2998 PDA 239.0 nm (2998 (210-800)nm)

HPLC chromatograms of L14a–c

L14a

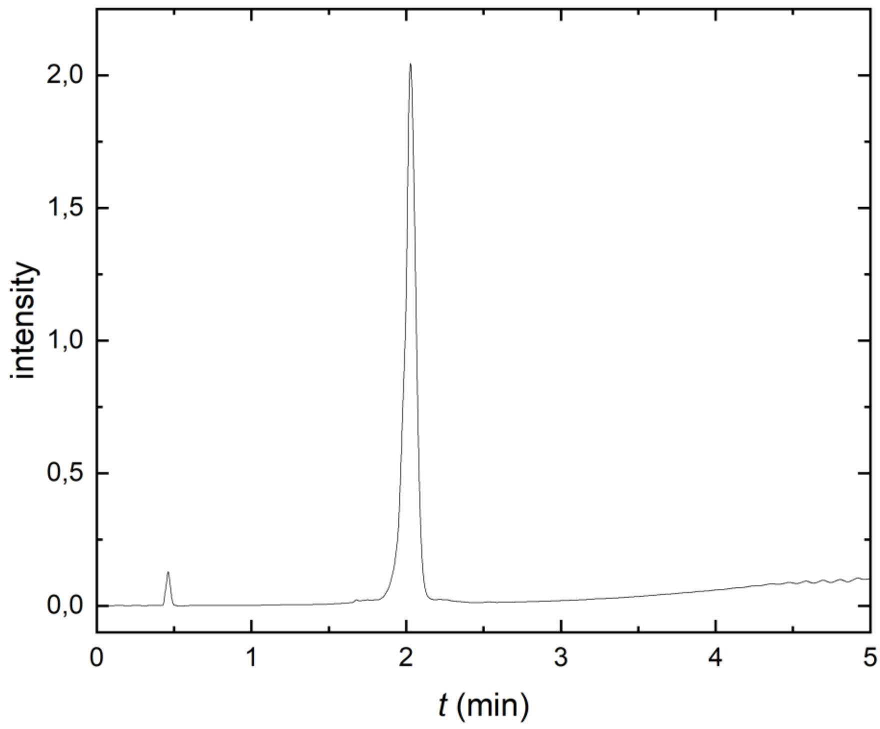

L14b

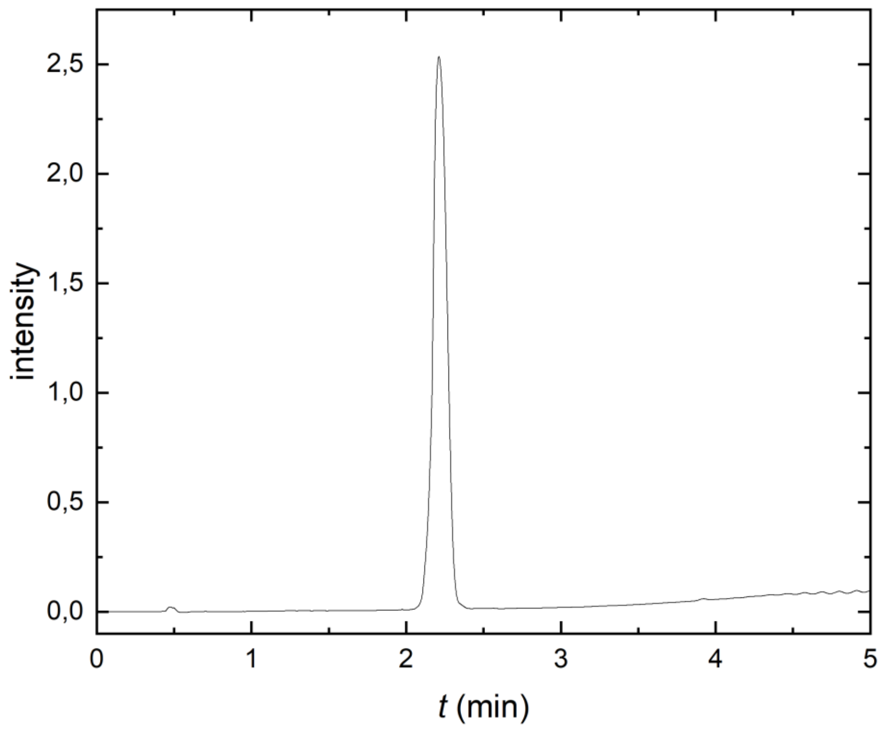

L14c

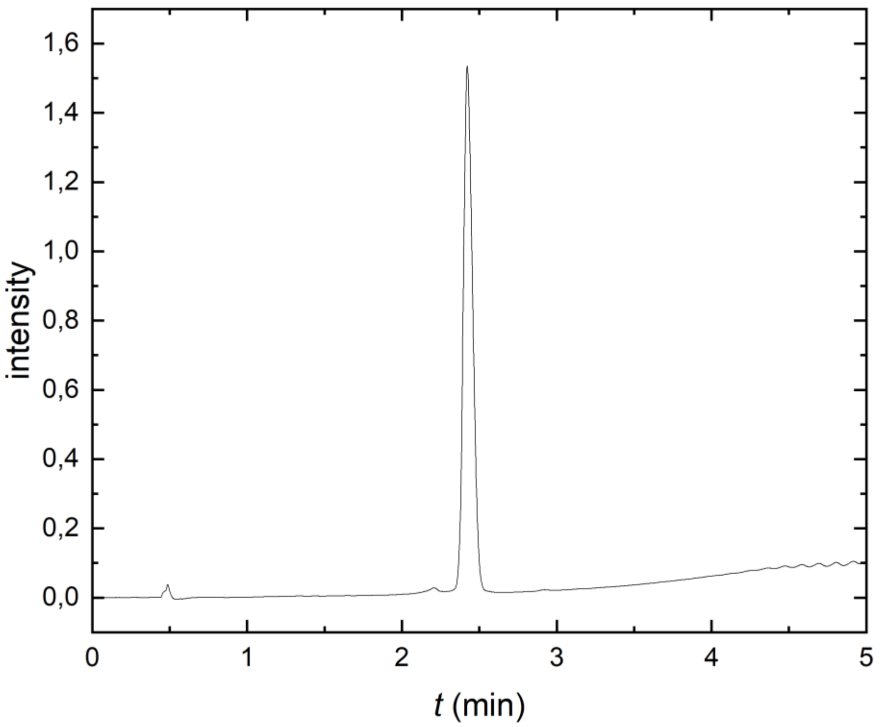

Kinetic of hydrolysis of BNPP by Cu(II)–L complexes

Cu(II)–L1

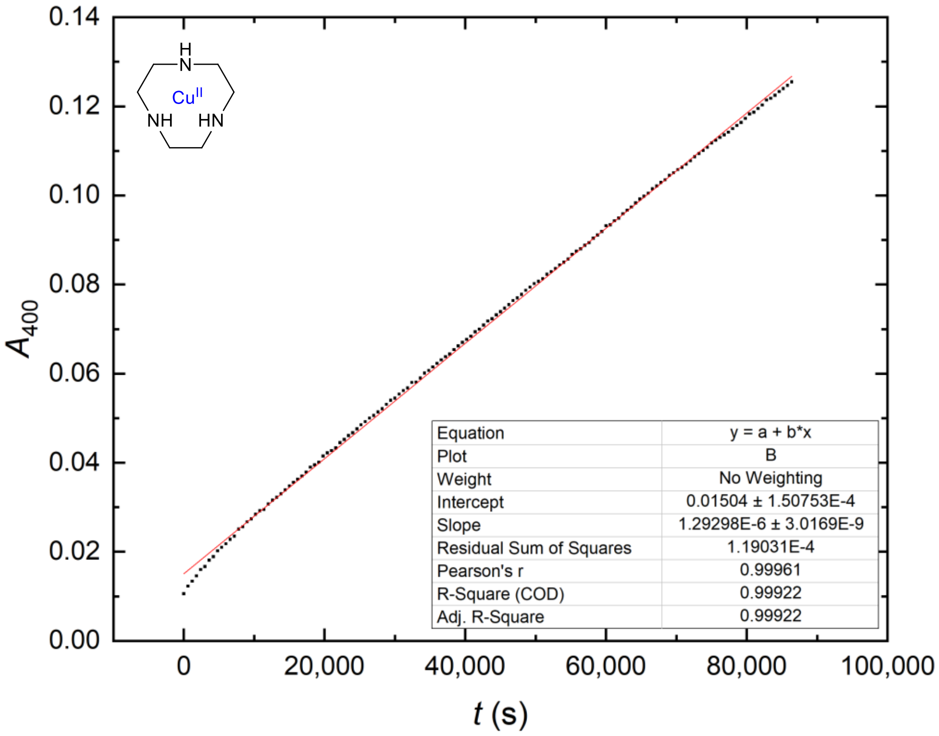

Cu(II)–L2

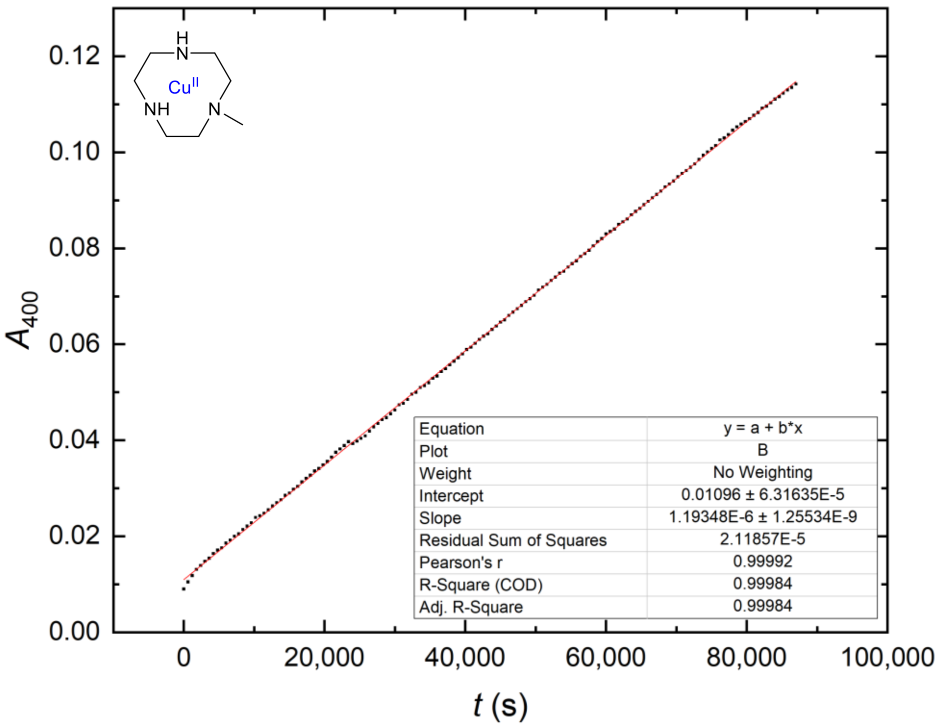

Cu(II)–L3

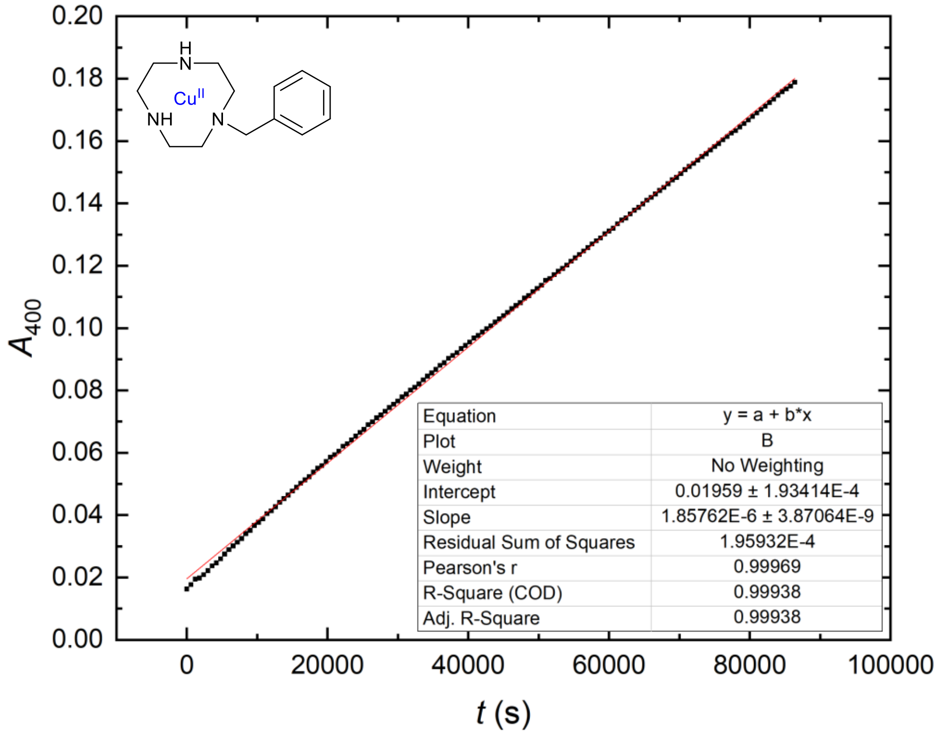

Cu(II)–L4

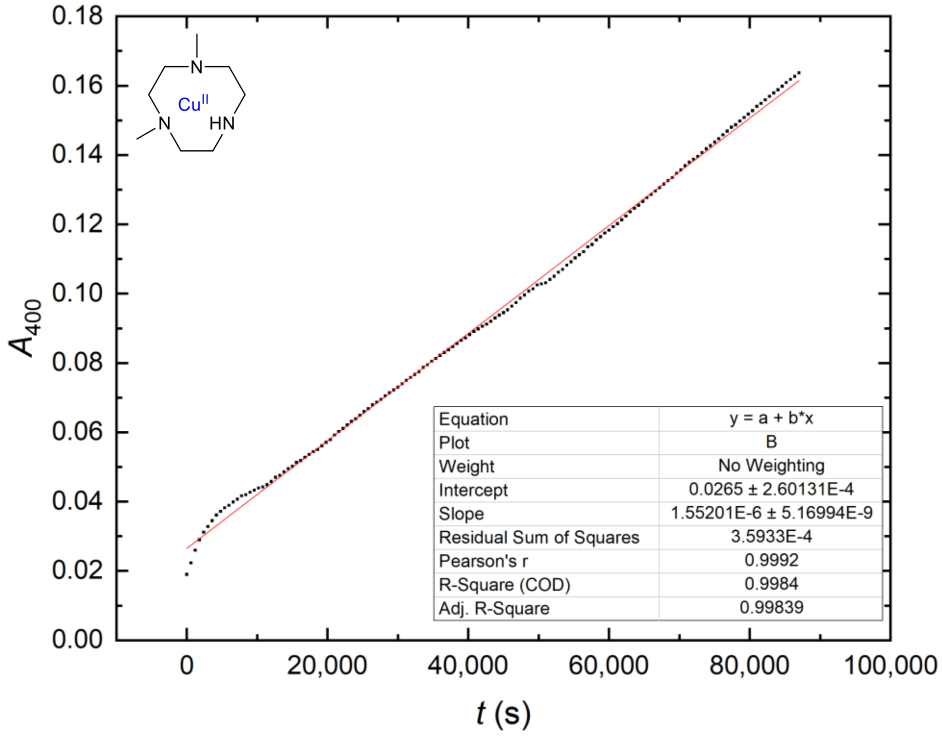

Cu(II)–L5

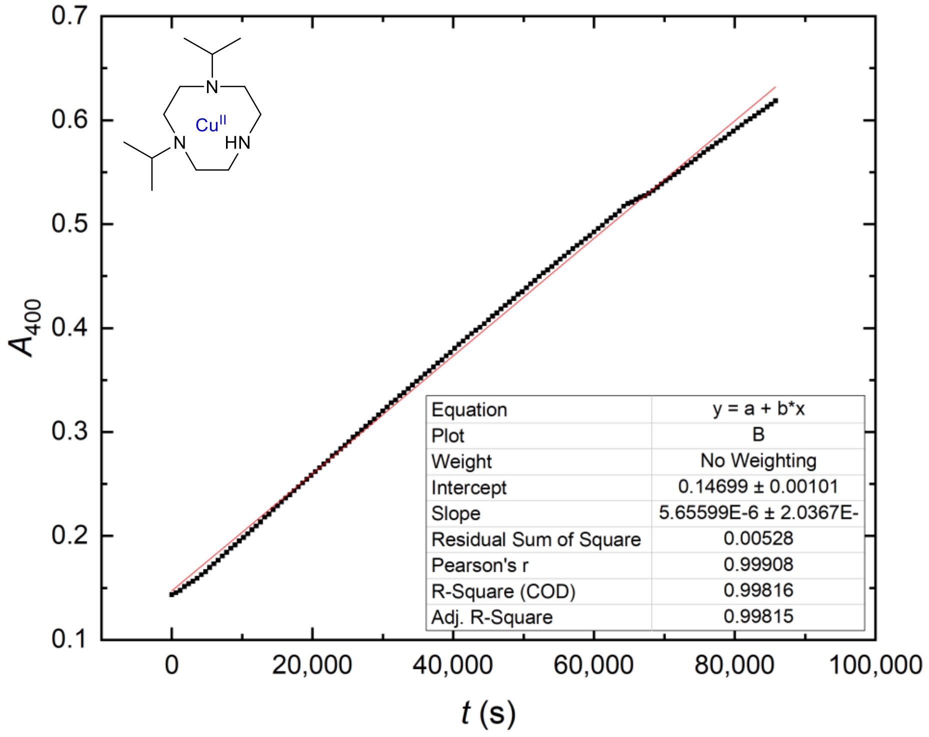

Cu(II)–L6

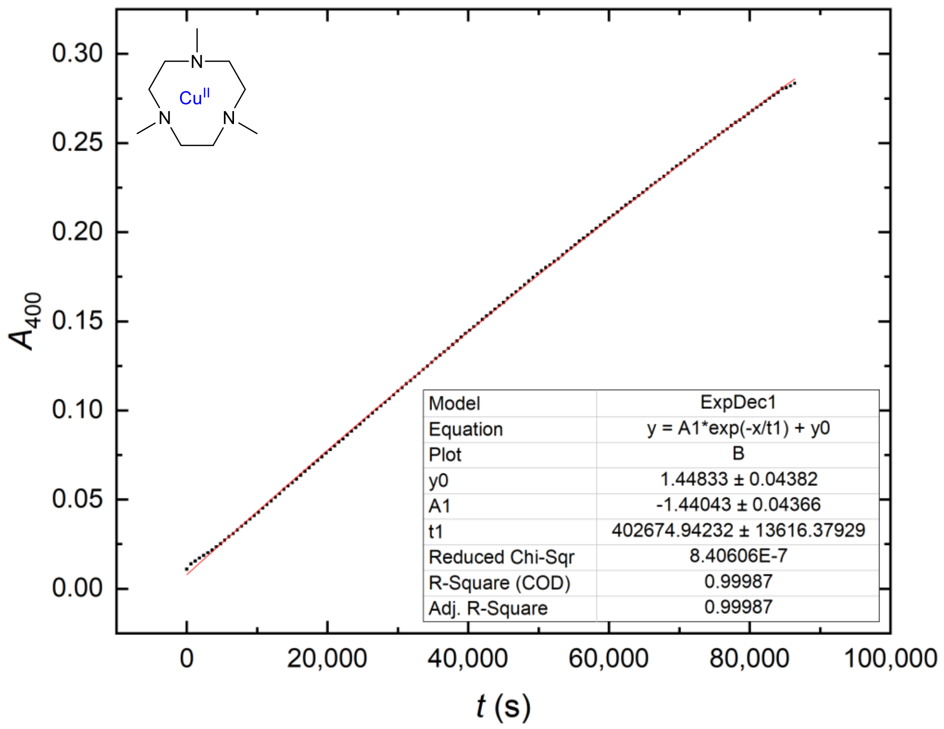

Cu(II)–L8

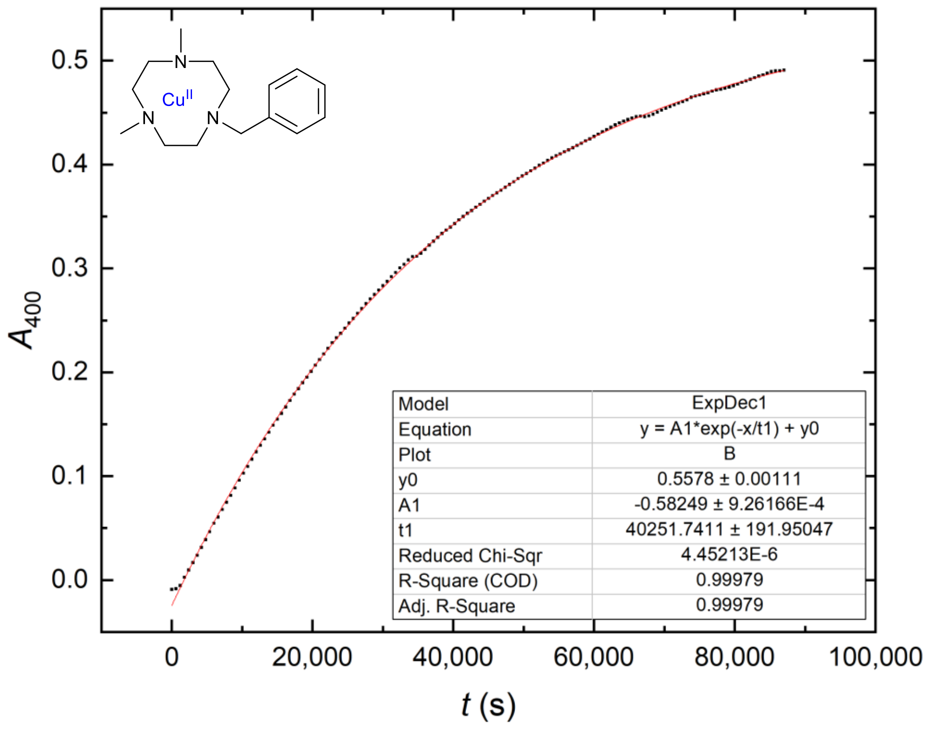

Cu(II)–L9

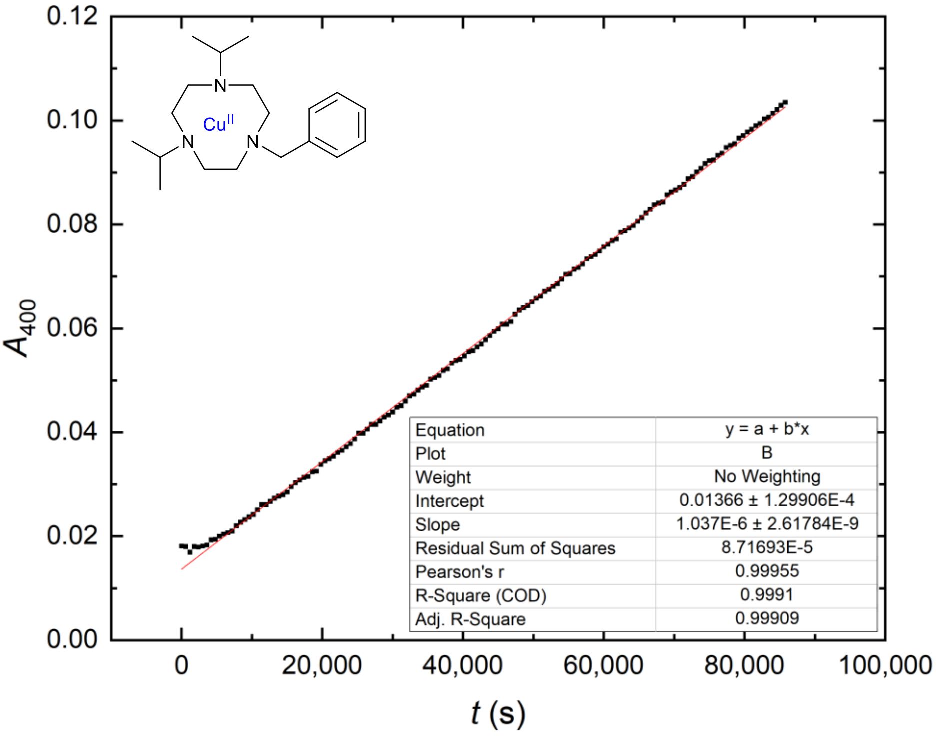

Cu(II)–L10b

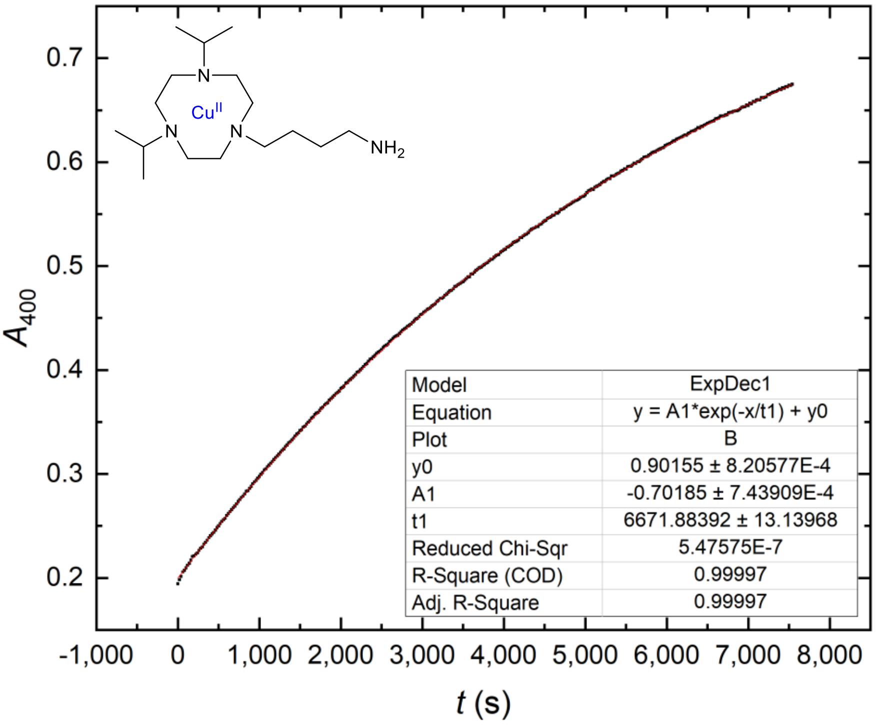

Cu(II)–L10c

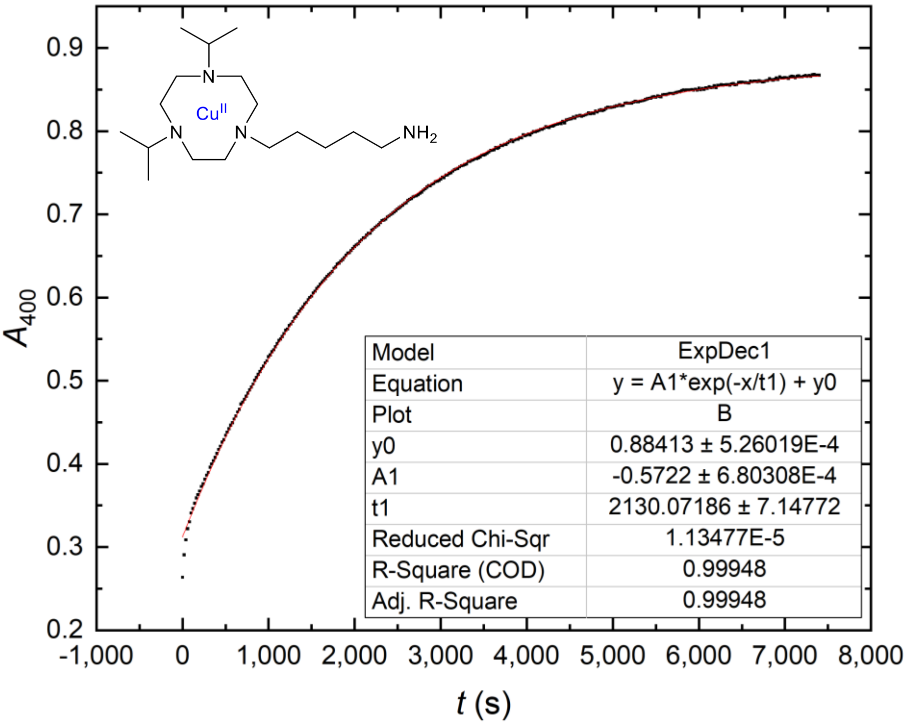

Cu(II)–L11b

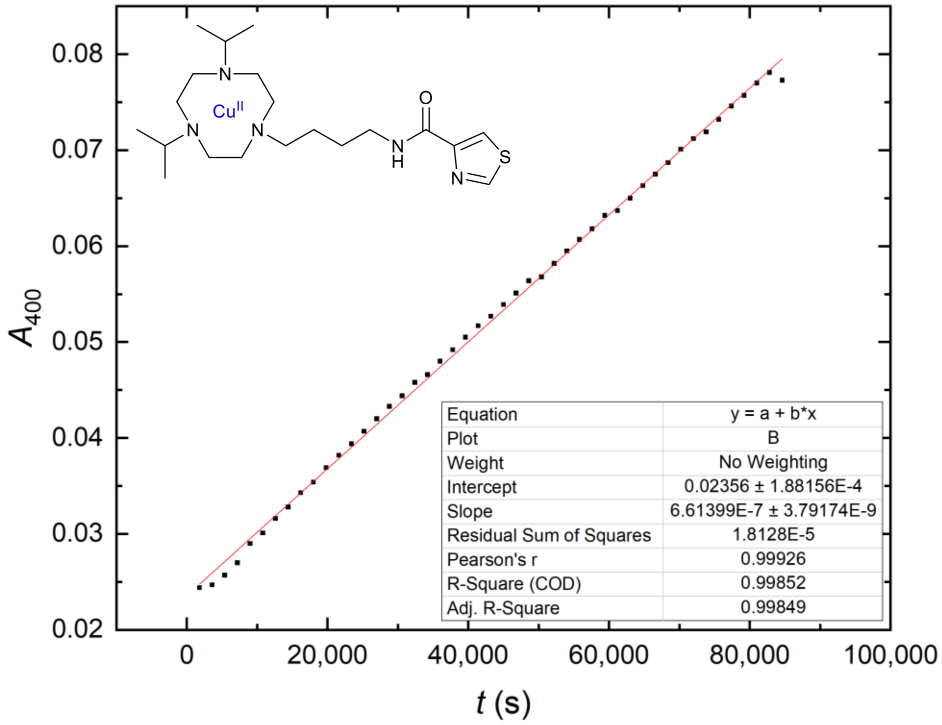

Cu(II)–L11c

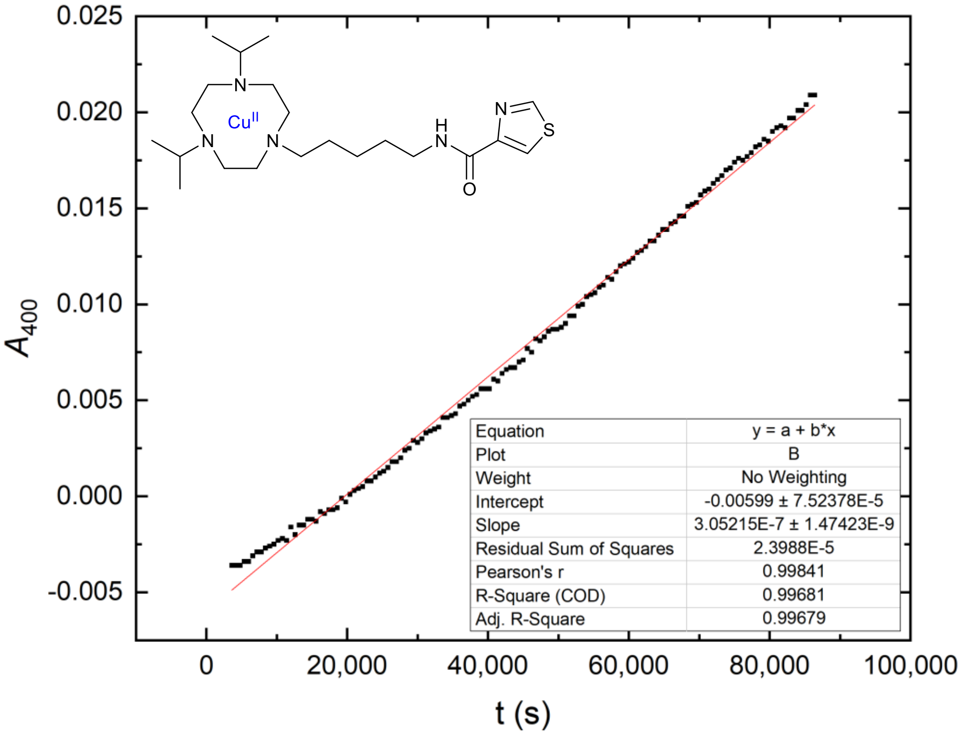

Cu(II)–L13a

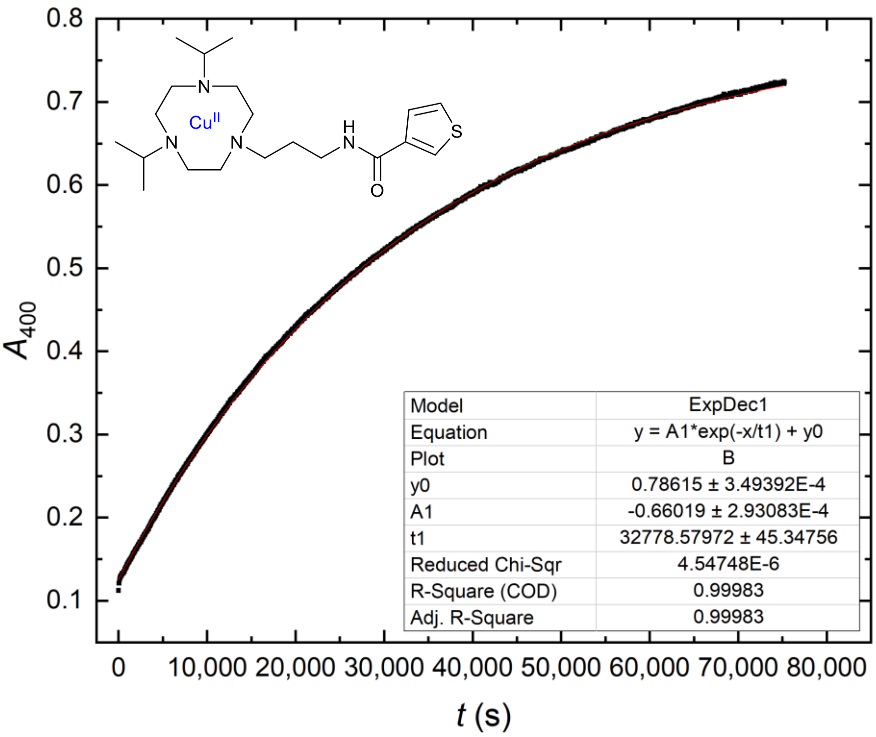

Cu(II)–L13b

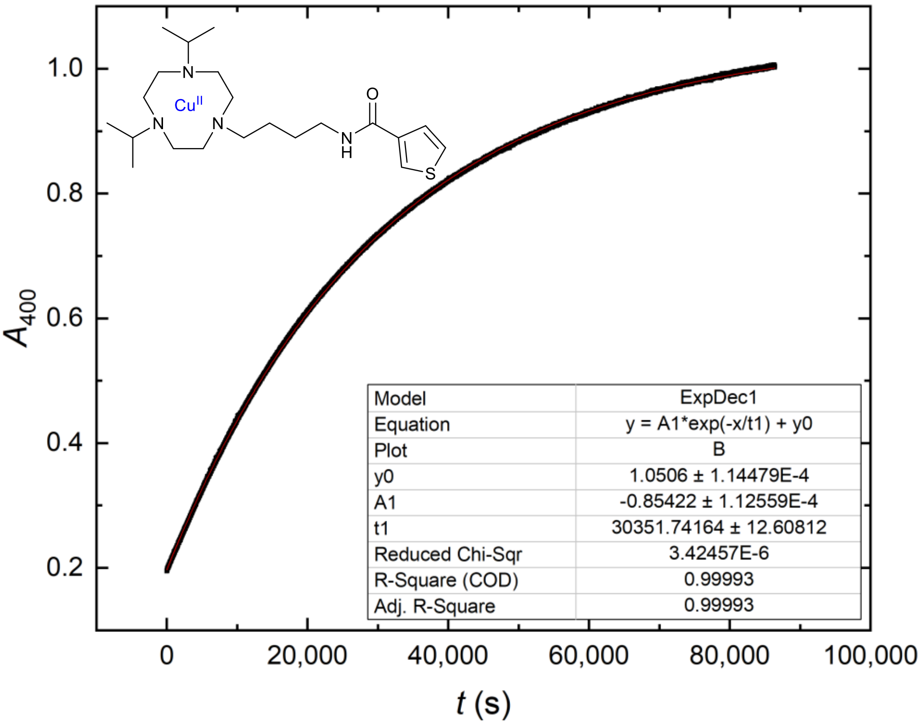

Cu(II)–L13c

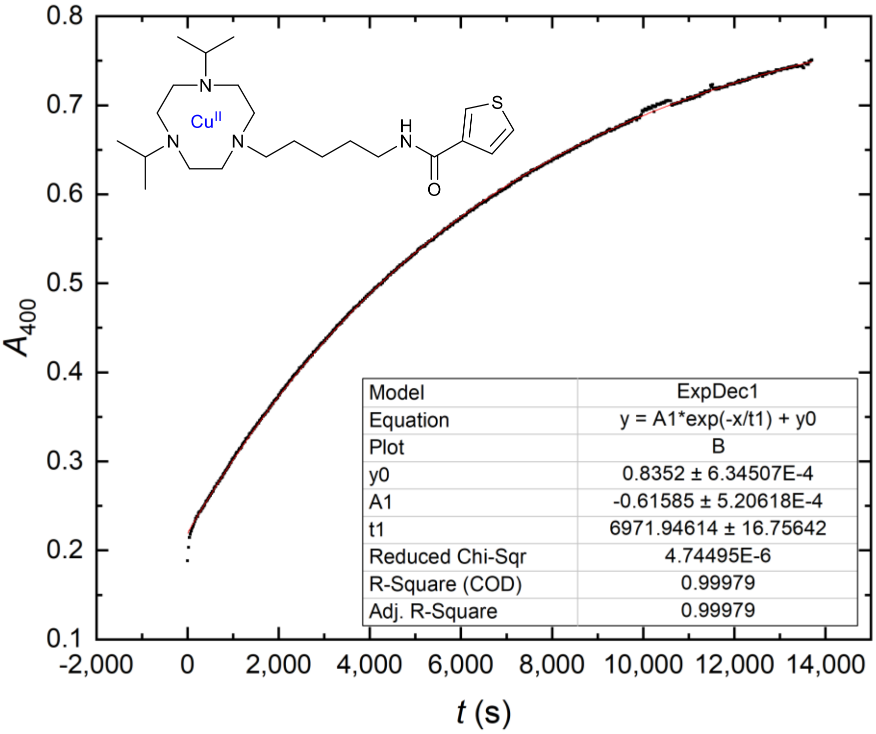

Cu(II)–L14a

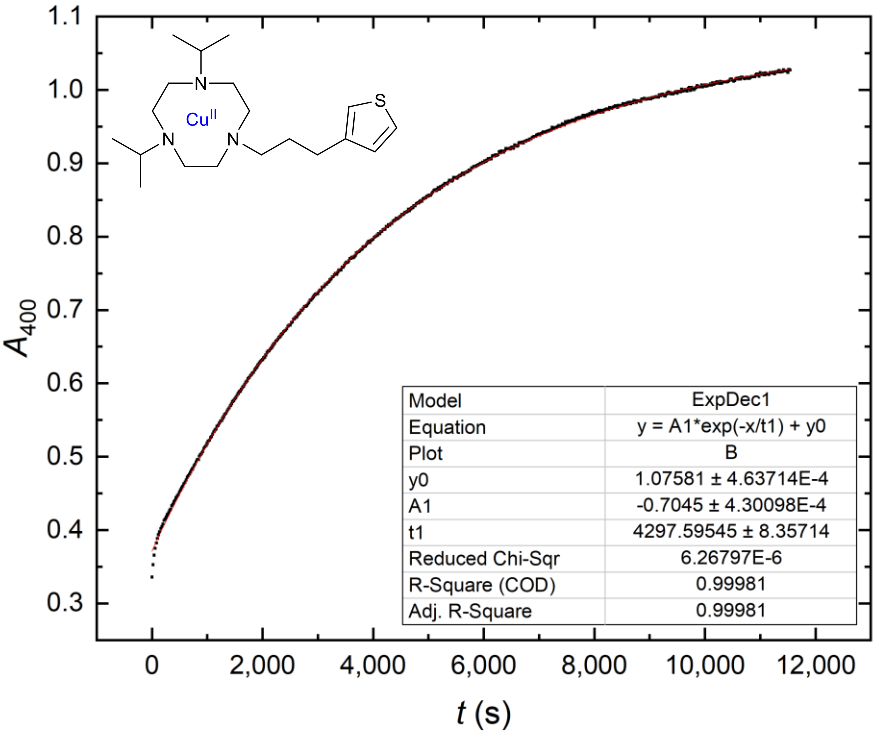

Cu(II)–L14b

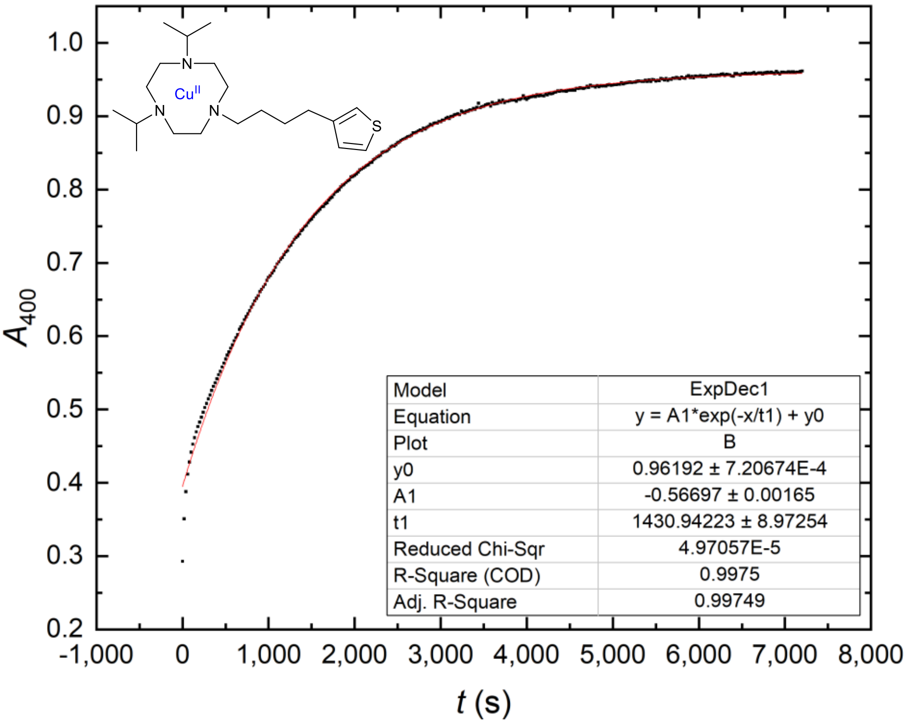

Cu(II)–L14c

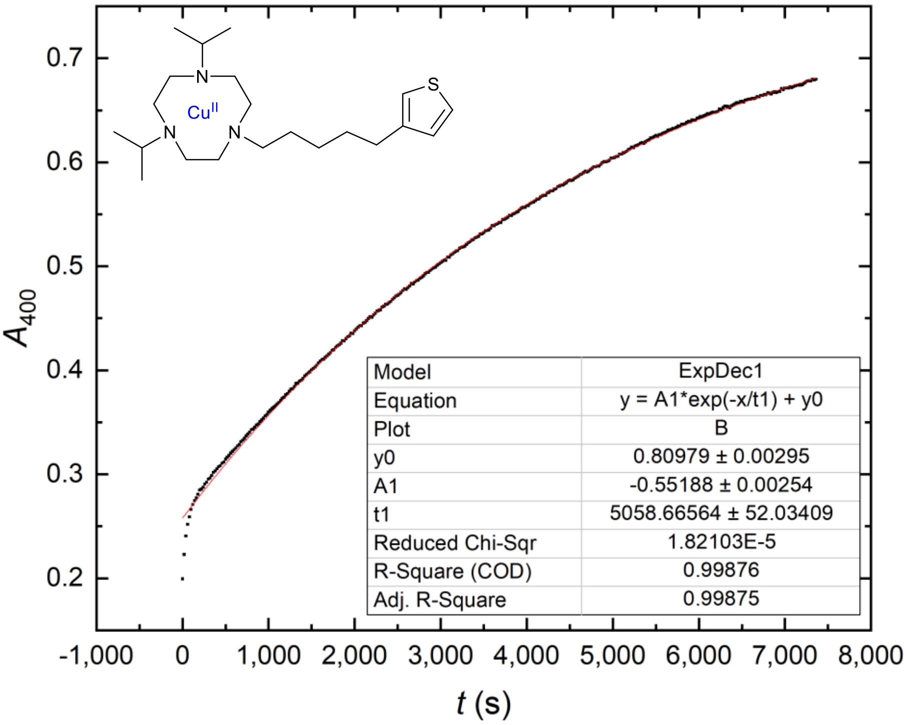

Calibration curve of para nitrophenol at pH 7.50 and 25 °C

Absorption extinction coefficient obtained from the experiment: 12644 mol·dm^{-3}·cm^{-1}.

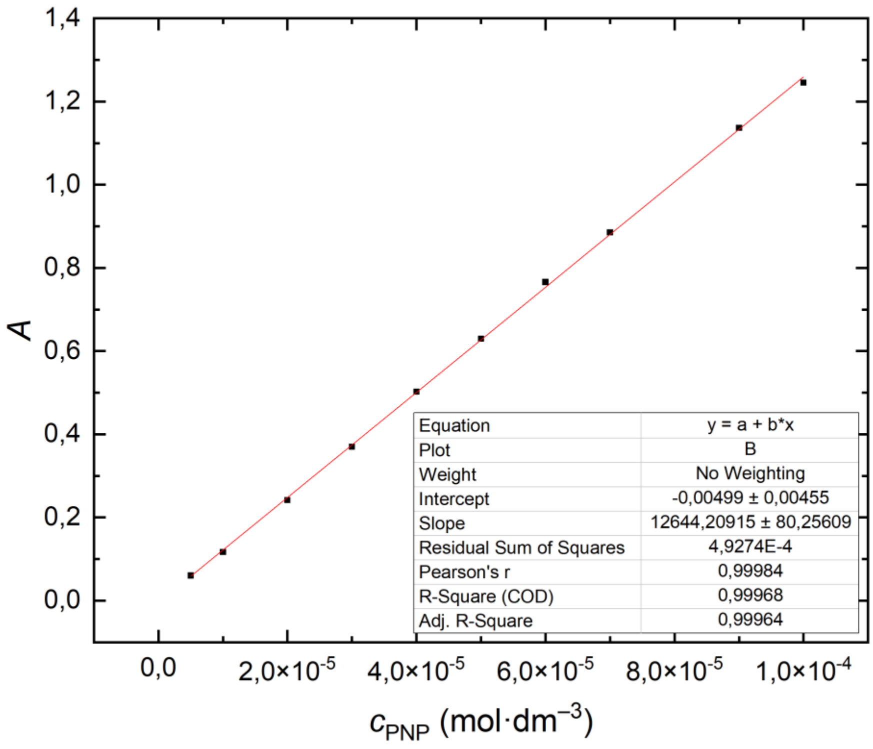

Supplement: Supplementary file 1 [file molecules-28-07542-s001.zip › molecules-2694795-supplementary.pdf]
